# Higher Dispersion Measures of Conduction and Repolarization in Type 1 Compared to Non-type 1 Brugada Syndrome Patients: An Electrocardiographic Study From a Single Center

**DOI:** 10.3389/fcvm.2018.00132

**Published:** 2018-10-04

**Authors:** Gary Tse, Ka Hou Christien Li, Guangping Li, Tong Liu, George Bazoukis, Wing Tak Wong, Matthew T. V. Chan, Martin C. S. Wong, Yunlong Xia, Konstantinos P. Letsas, Gary Chin Pang Chan, Yat Sun Chan, William K. K. Wu

**Affiliations:** ^1^Department of Medicine and Therapeutics, Faculty of Medicine, Chinese University of Hong Kong, Hong Kong, China; ^2^Li Ka Shing Institute of Health Sciences, Faculty of Medicine, Chinese University of Hong Kong, Hong Kong, China; ^3^Faculty of Medicine, Newcastle University, Newcastle upon Tyne, United Kingdom; ^4^Tianjin Key Laboratory of Ionic-Molecular Function of Cardiovascular Disease, Department of Cardiology, Tianjin Institute of Cardiology, Second Hospital of Tianjin Medical University, Tianjin, China; ^5^Laboratory of Cardiac Electrophysiology, Second Department of Cardiology, Evangelismos General Hospital of Athens, Athens, Greece; ^6^School of Life Sciences, The Chinese University of Hong Kong, Hong Kong, China; ^7^Department of Anaesthesia and Intensive Care, The Chinese University of Hong Kong, Hong Kong, China; ^8^The Jockey Club School of Public Health and Primary Care, The Chinese University of Hong Kong, Hong Kong, China; ^9^Department of Cardiology, First Affiliated Hospital of Dalian Medical University, Dalian, China

**Keywords:** electrocardiography, conduction, repolarization, wavelength, Brugada syndrome

## Abstract

**Background:** Brugada syndrome (BrS) is a cardiac ion channelopathy that predisposes affected individuals to sudden cardiac death (SCD). Type 1 BrS is thought to take a more malignant clinical course than non-type 1 BrS. We hypothesized that the degrees of abnormal repolarization and conduction are greater in type 1 subjects and these differences can be detected by electrocardiography (ECG).

**Methods:** Electrocardiographic data from spontaneous type 1 and non-type 1 BrS patients were analyzed. ECG parameters were measured from leads V1 to V3. Values were expressed as median [lower quartile-upper quartile] and compared using Kruskal-Wallis ANOVA.

**Results:** Compared to non-type 1 BrS patients (*n* = 29), patients with spontaneous type 1 patterns (*n* = 22) showed similar (*P* > 0.05) heart rate (73 [64–77] vs. 68 [62–80] bpm), QRS duration (136 [124–161] vs. 127 [117–144] ms), uncorrected QT (418 [393–443] vs. 402 [386–424] ms) and corrected QT intervals (457 [414–474] vs. 430 [417–457] ms), JT_peak_ intervals (174 [144–183] vs. 174 [150–188] ms), T_peak−_ T_end_ intervals (101 [93–120] vs. 99 [90–105] ms), T_peak−_ T_end_/QT ratios (0.25 [0.23–0.27] vs. 0.24 [0.22–0.27]), T_peak−_ T_end_/QRS (0.77 [0.62–0.87] vs. 0.77 [0.69–0.86]), T_peak−_ T_end_/(QRS × QT) (0.00074 [0.00034–0.00096] vs. 0.00073 [0.00048–0.00012] ms^−1^), index of Cardiac Electrophysiological Balance (iCEB, QT/QRS, marker of wavelength: 3.14 [2.56–3.35] vs. 3.21 [2.85–3.46]) and corrected iCEB (QTc/QRS: 3.25 [2.91–3.73] vs. 3.49 [2.99–3.78]). Higher QRS dispersion was seen in type 1 subjects (QRSd: 34 [24–66] vs. 24 [12–34] ms) but QT dispersion (QTd: 48 [39–71] vs. 43 [22–94] ms), QTc dispersion (QTcd: 52 [41–79] vs. 46 [23–104] ms), JT_peak_ dispersion (44 [23–62] vs. 45 [30–62] ms), T_peak−_ T_end_ dispersion (28 [15–34] vs. 29 [22–53] ms) or T_peak−_ T_end_/QT dispersion (0.06 [0.03–0.08] vs. 0.08 [0.04–0.12]) did not differ between the two groups. Type 1 subjects showed higher (QRSd × T_peak−_ T_end_)/QRS (25 [19–44] vs. 19 [9–30] ms) but similar iCEB dispersion (0.83 [0.49–1.14] vs. 0.61 [0.34–0.92]) and iCEBc dispersion (0.93 [0.51–1.15] vs. 0.65 [0.39–0.96]).

**Conclusion:** Higher levels of dispersion in conduction and repolarization are found in type 1 than non-type 1 BrS patients, potentially explaining the higher incidence of ventricular arrhythmias in the former group.

## Introduction

Brugada syndrome (BrS) is a cardiac ion channelopathy that predisposes affected individuals to ventricular tachyarrhythmias and sudden cardiac death (SCD). Type 1 BrS is thought to take a more malignant clinical course than non-type 1 BrS (1). Abnormalities in both conduction and repolarization processes contribute to ventricular tachyarrhythmias in BrS (2). For instance, slow and discontinuous conduction of action potentials through working myocardium, due to reduced sodium channel activity, may lead to higher degrees of spatial and temporal dispersion in conduction (3). These could potentially be detected as prolonged QRS intervals (4) and higher QRS dispersion (5). Moreover, heterogeneous time-course in full repolarization between the different myocardial layers, due to regional difference in transient outward potassium channel activity, leads to increased transmural repolarization gradients that can be measured electrographically using QT dispersion (QT_d_) (6, 7), interval from the peak to the end of the T-wave (8) [T_peak_ – T_end_, reflecting transmural dispersion of repolarization, TDR (9)], (T_peak_ – T_end_)/QT ratio (10, 11) and T_peak_ – T_end_ dispersion. However, the present electrocardiographic indices do not incorporate parameters on dispersion and these may play important roles in producing the reentrant substrate for arrhythmogenesis (12). In this study, we hypothesized that the degree of abnormal repolarization and conduction is greater in spontaneous type 1 subjects and these differences can be detected by electrocardiographic indices incorporating spatial dispersion of conduction and repolarization.

## Methods

### Study subjects

This retrospective study received ethics approval from the NTEC-CUHK Clinical Research Ethics Committee. Inclusion criteria include subjects diagnosed with Brugada Syndrome presented to the Prince of Wales Hospital, a tertiary level teaching hospital in Hong Kong, China. Age, sex, type of Brugada pattern (spontaneous type 1 or otherwise), syncopal symptoms and spontaneous VT or VF were recorded.

### Electrocardiographic measurements

The following parameters were obtained from 12-lead electrocardiograms of spontaneous type 1 (Data Sheet 1) and non-type 1 (Data Sheet 2) Brugada subjects. Measurements were made from the right precordial leads (V1–V3) with mean values calculated. They were measured together by GT and CL using Phillips ECGVue (Standard Edition). The first ten measurements were validated by clinical electrophysiologists of our centers (KPL and JC). The end of the T-wave was determined using the return to the baseline method. Dispersion was defined as the difference between the maximum and minimum value detected from V1 to V3.

Repolarization parameters including QT interval (onset of the QRS complex to the end of the T wave at T-P baseline; If U waves are present, the QT interval will be taken to the nadir of the curve between the T and U waves), QTc (correction using Bazett's formula), QT dispersion, T_peak_ – T_end_ (peak of T-wave to end of T-wave), T_peak_ – T_end_ dispersion, T_peak_ – T_end_/QT ratio, T_peak_ – T_end_/QT dispersion, and JT_peak_ (J point to peak of T-wave), and JT_peak_ dispersion. Conduction parameters include QRS duration (onset of Q-wave to the terminal portion of S-wave) and QRS dispersion. Conduction-repolarization indices include index of Cardiac Electrophysiological Balance (iCEB, QT/QRS, a surrogate marker of excitation wavelength), iCEBc (QTc/QRS), their dispersion parameters, (T_peak_ – T_end_)/QRS, T_peak_ – T_end_/(QT × QRS) and QRS_d_ × (T_peak_ – T_end_)/QRS.

### Statistical analysis

Data were expressed as median [lower quartile to upper quartile]. Categorical data were analyzed by Fisher's exact test. Differences between study groups were tested using Kruskal-Wallis ANOVA. *P* < 0.05 was considered statistically significant.

## Results

### Clinical characteristics

**Figure 1:**
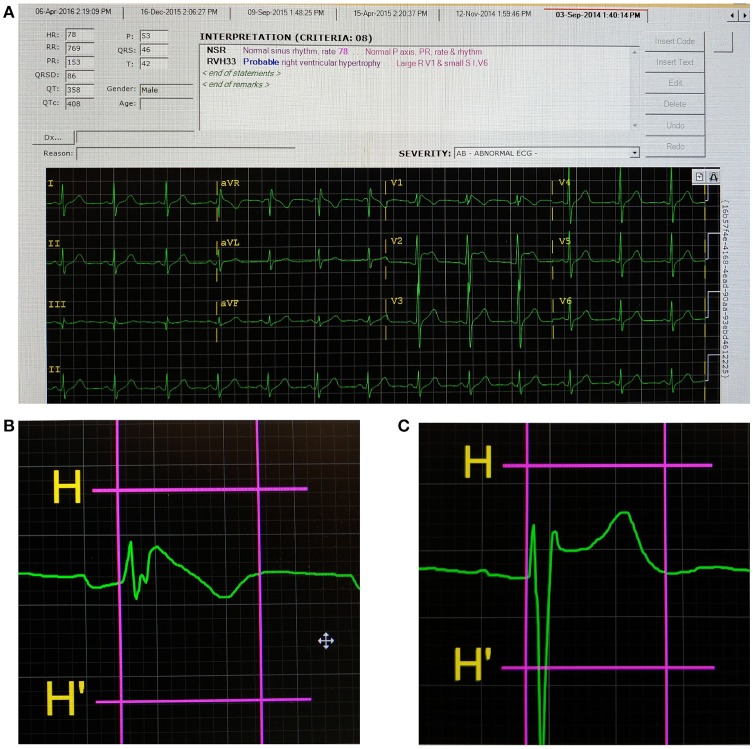
Screenshot of the ECG analysis program **(A)**, a Type 1 Brugada pattern **(B)**, and non-Type 1 Brugada pattern **(C)**. The first and second vertical lines indicate the onset of the QRS complex and end of the T-wave, respectively, for **(B,C)**.

This study included a total of 51 Brugada syndrome patients. The baseline demographic and clinical characteristics are shown in Table 1. The mean age was 56 ± 2 years and 90% of the subjects were male. A type 1 pattern was observed in 22 patients (43%) and a non-type 1 pattern was observed in 29 patients (57%). Implantable cardioverter-defibrillators were inserted in 21 (71%) subjects. 25 (49%) subjects had syncope, and spontaneous VT was observed in 7 patients. Compared to non-type 1 subjects, type 1 subjects were more likely to have ICD implanted (68 vs. 21%, *P* = 0.0005) and suffer from syncope (68 vs. 34%, *P* = 0.02). However, no difference in age, appropriate ICD shocks or spontaneous VT was observed between the groups (*P* > 0.05). Resting heart rate was similar between type 1 and non-type 1 subjects (73 [64–77] vs. 68 [62–80] bpm, respectively; *P* = 0.78). The different electrocardiographic parameters were measured from the precordial leads V1–V3 and mean values were calculated. Dispersion was defined as the difference in the maximum and minimum values observed in leads V1–V3. Example screenshots of the ECG measurement system, a spontaneous Type 1 Brugada pattern and non-Type 1 Brugada pattern are shown in Figures 1A–C, respectively. The positions of the onset of the QRS complex and the end of the T-wave are represented by the vertical lines.

**Table 1:** Demographic and clinical characteristics of Brugada syndrome patients included in this study (*n* = 51).

**Characteristics**	**Type 1 BrS (*n* = 22)**	**Non-type 1 BrS (*n* = 29)**	***P*-value**
Male sex	20 (91%)	26 (92%)	0.6298
Age (years)^ψ^	58.5 (51.5–67.0)	57.0 (36.0–70.0)	0.6343
ICD insertion	15 (68%)	6 (21%)	0.0005
Appropriate ICD shocks	3 (14%)	1 (3%)	0.2966
Syncope	15 (68%)	10 (34%)	0.0245
Spontaneous VT	5 (23%)	2 (17%)	0.2163

*Data were presented as number (%), ^ψ^median (lower quartile to upper quartile). P-value were obtained from Fisher's exact test (for frequency data) or Kruskal-Wallis ANOVA (for continuous data)*.

### Traditional conduction or repolarization markers: QRS, QT, QTc, and JT_peak_ intervals

**Figure 2:**
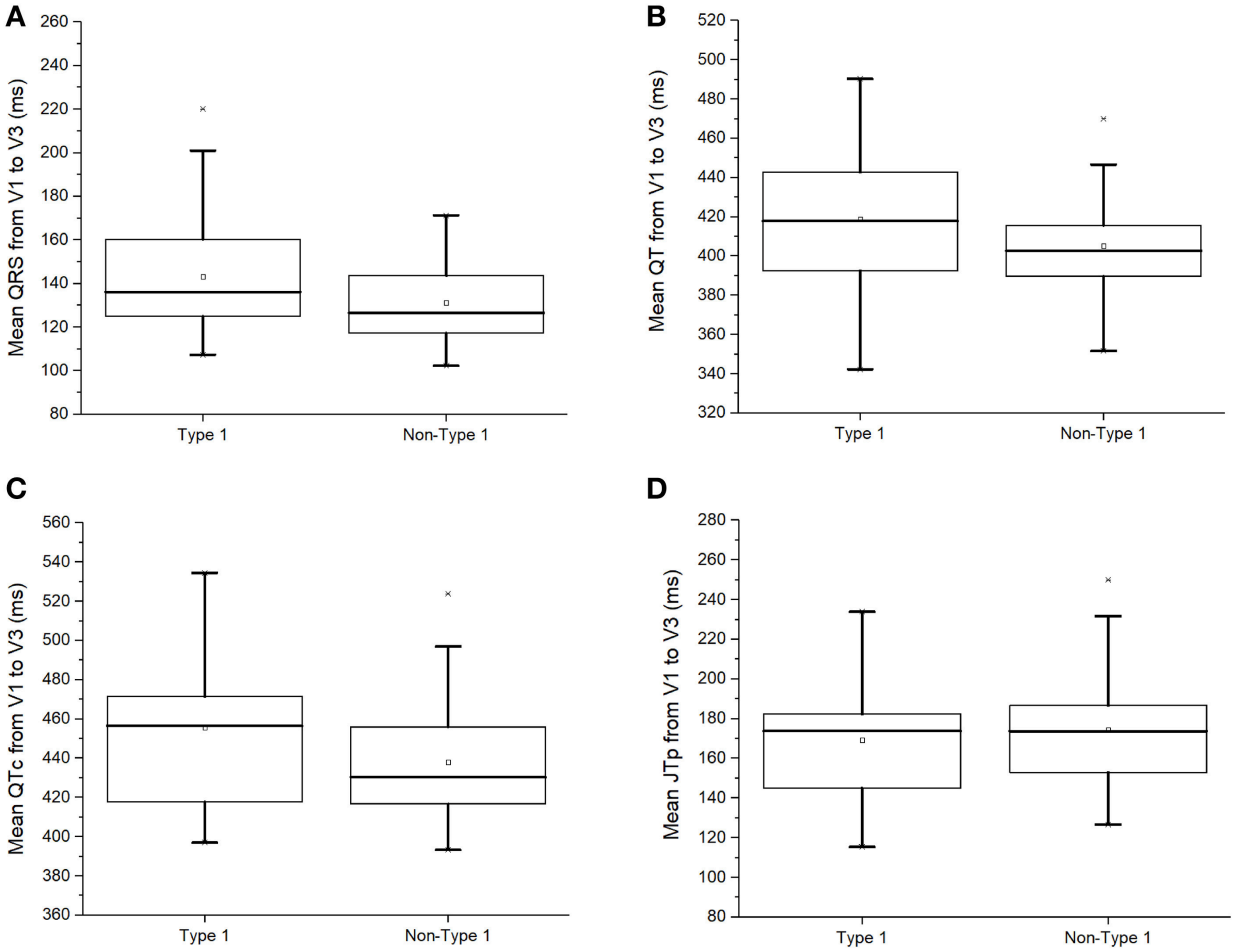
QRS duration **(A)**, uncorrected QT interval **(B)**, corrected QT interval **(C)**, or JT_peak_ interval **(D)** in type 1 and non-type 1 Brugada syndrome patients.

Compared to non-type 1 BrS subjects, those with type 1 BrS had statistically indistinguishable QRS duration (136 [124–161] vs. 127 [117–144] ms; *P* = 0.14; Figure 2A), uncorrected QT (418 [393–443] vs. 402 [386–424] ms; *P* = 0.17; Figure 2B) and corrected QT intervals using Bazett's formula (457 [414–474] vs. 430 [417–457] ms; *P* = 0.15; Figure 2C). Moreover, JT_peak_ intervals, which are useful for assessing repolarization duration in the context of slowed ventricular conduction (13), were not significantly different between type 1 and non-type 1 BrS patients (174 [144–183] vs. 174 [150–188] ms; *P* = 0.52; Figure 2D).

### Markers of repolarization or conduction dispersion

**Figure 3:**
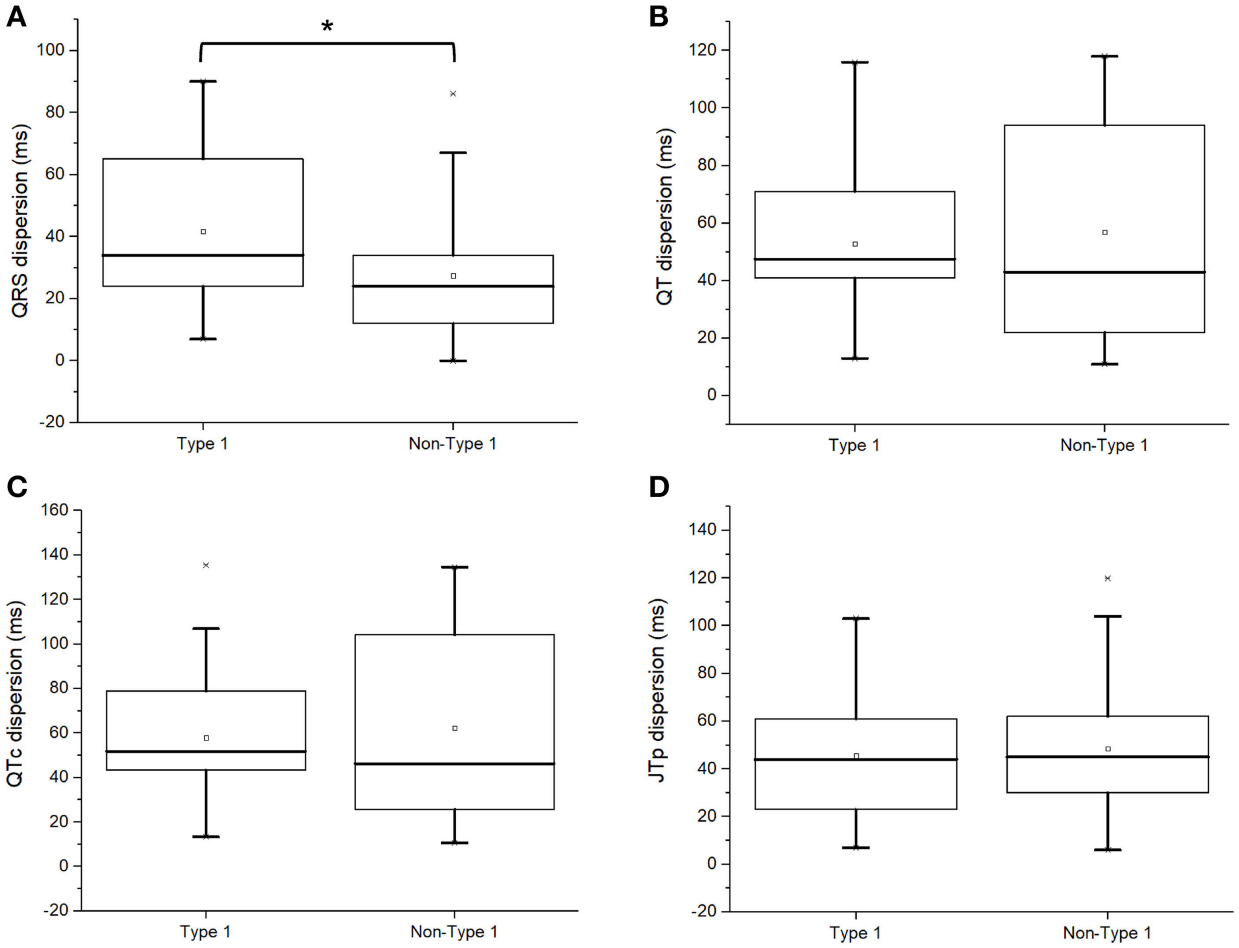
QRS dispersion **(A)**, uncorrected QT dispersion **(B)**, corrected QT dispersion **(C)**, or JT_peak_ dispersion **(D)** in type 1 and non-type 1 Brugada syndrome patients. *Denotes significant difference between the two groups.

The conduction dispersion marker, QRS dispersion, was significantly higher in type 1 subjects (QRSd: 34 [24–66] vs. 24 [12–34] ms; *P* = 0.03; Figure 3A). By contrast, the repolarization dispersion markers, QT dispersion (QTd: 48 [39–71] vs. 43 [22–94] ms; *P* = 0.98 Figure 3B), QTc dispersion (QTcd: 52 [41–79] vs. 46 [23–104] ms; *P* = 0.98; Figure 3C), JT_peak_ dispersion (44 [23–62] vs. 45 [30–62] ms; *P* = 0.77; Figure 3D) were statistically indistinguishable between both groups.

**Figure 4:**
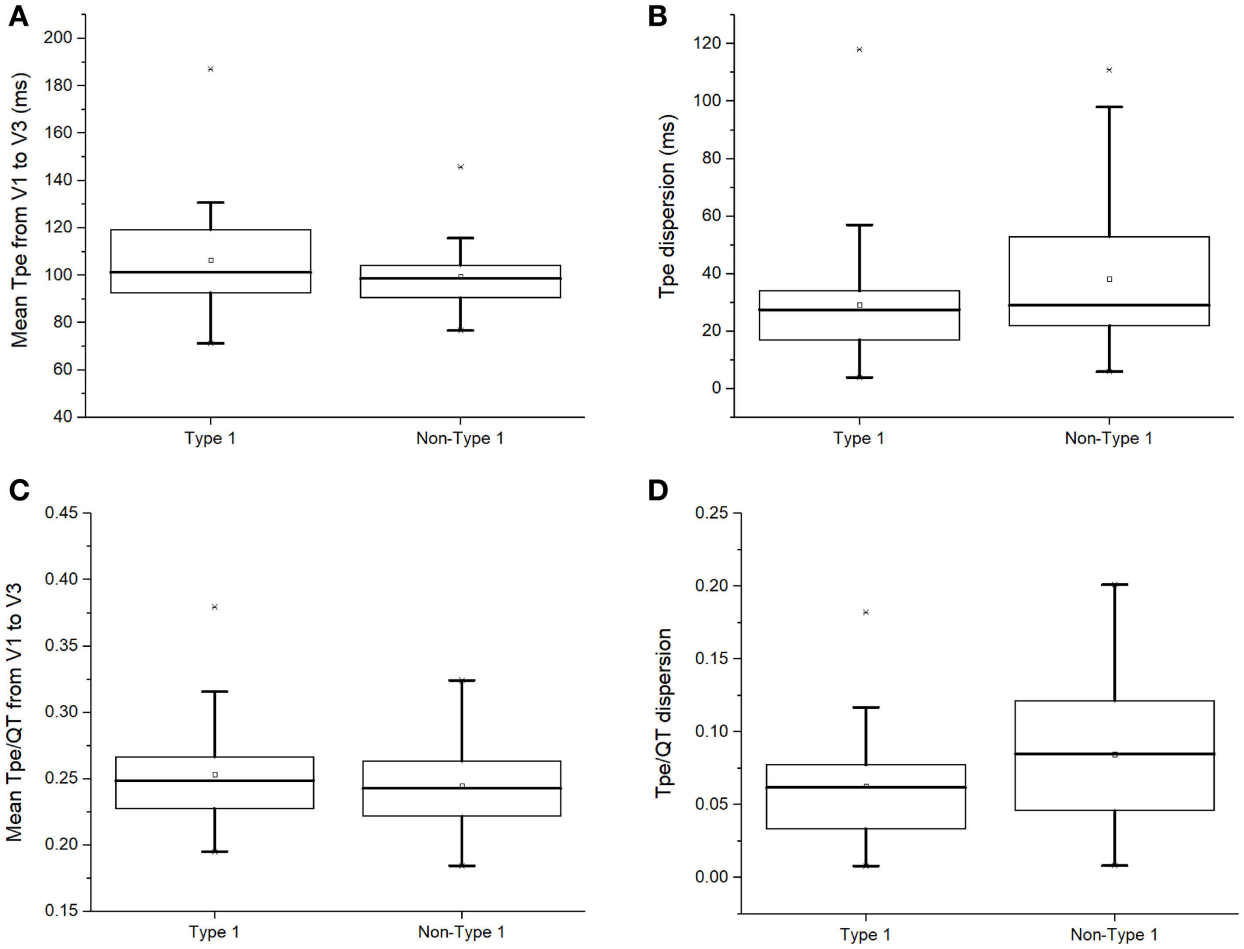
T_peak−_ T_end_ intervals **(A)**, T_peak−_ T_end_ dispersion **(B)**, T_peak−_ T_end_/QT ratios **(C)**, or T_peak−_ T_end_/QT dispersion **(D)** in type 1 and non-type 1 Brugada syndrome patients.

Moreover, T_peak−_ T_end_ indices reflecting global or transmural dispersion of repolarization were studied. T_peak−_ T_end_ intervals (101 [93–120] vs. 99 [90–105] ms; *P* = 0.28; Figure 4A), T_peak−_ T_end_ dispersion (28 [15–34] vs. 29 [22–53] ms; *P* = 0.18; Figure 4B), T_peak−_ T_end_/QT ratios (0.25 [0.23–0.27] vs. 0.24 [0.22–0.27]; *P* = 0.56; Figure 4C), or T_peak−_ T_end_/QT dispersion (0.06 [0.03–0.08] vs. 0.08 [0.04–0.12]; *P* = 0.09; Figure 4D) did not differ between both groups.

### Markers of excitation wavelength and indices incorporating conduction and repolarization dispersion

**Figure 5:**
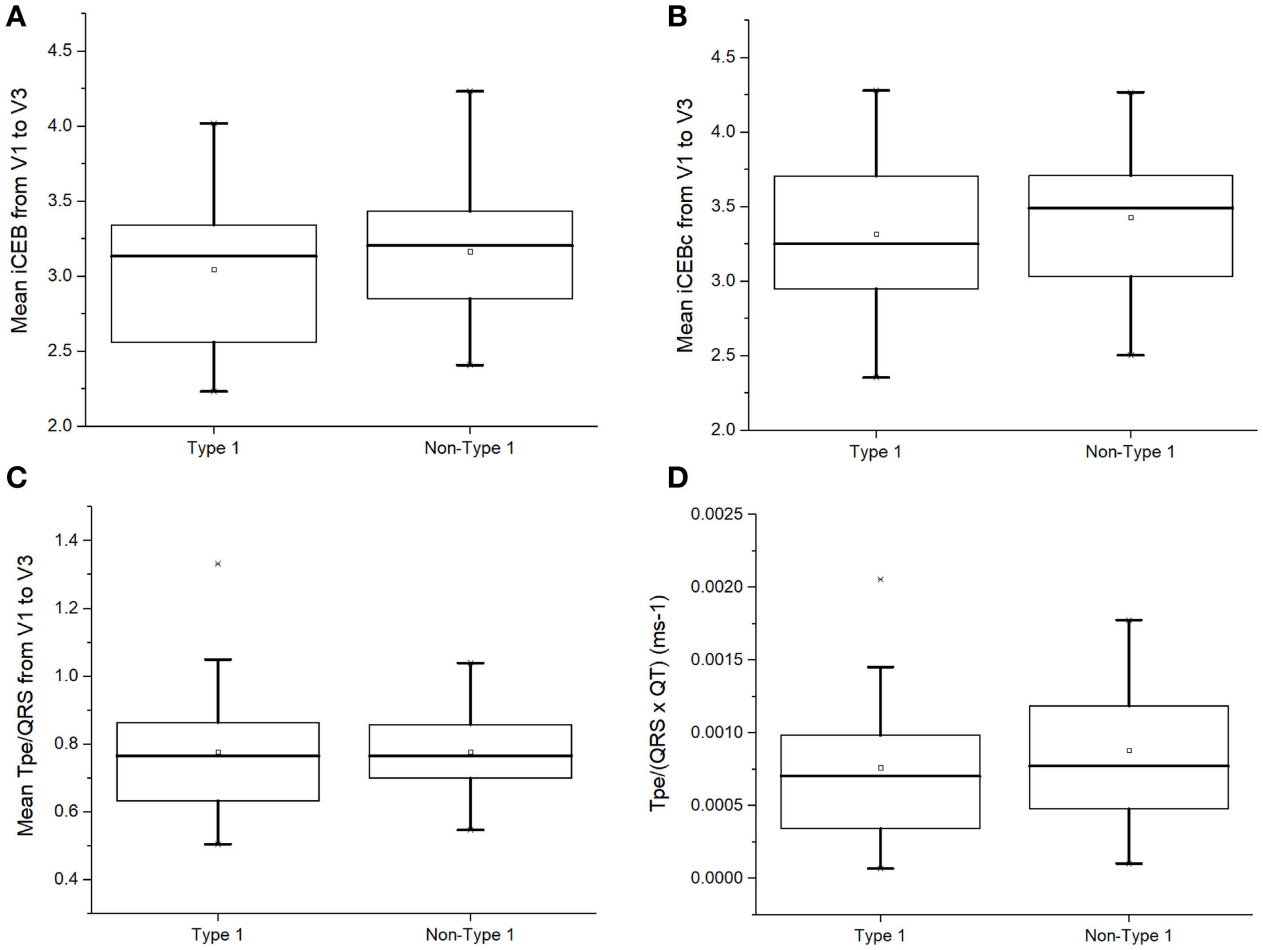
Index of Cardiac Electrophysiological Balance (iCEB, QT/QRS) **(A)**, iCEB corrected for heart rate (QTc/QRS) **(B)**, T_peak−_ T_end_/QRS **(C)**, or T_peak−_ T_end_/(QRS × QT) **(D)** in type 1 and non-type 1 Brugada syndrome patients.

Recently, the index of Cardiac Electrophysiological Balance (iCEB, QT/QRS) was proposed as a marker of excitation wavelength (14, 15). However, iCEB (3.14 [2.56–3.35] vs. 3.21 [2.85–3.46]; *P* = 0.45; Figure 5A) or iCEB corrected for heart rate (QTc/QRS: 3.25 [2.91–3.73] vs. 3.49 [2.99–3.78]; *P* = 0.48; Figure 5B) did not significantly differ between type 1 and non-type 1 BrS patients. Moreover, markers incorporating both repolarization and conduction dispersion, such as (T_peak_ – T_end_)/QRS, T_peak_ – T_end_/(QT × QRS) and QRS_d_ × (T_peak_ – T_end_)/QRS were proposed for risk stratification (16, 17). However, type 1 and non-type 1 BrS patients showed similar T_peak−_ T_end_/QRS (0.77 [0.62–0.87] vs. 0.77 [0.69–0.86]; *P* = 0.89; Figure 5C) and T_peak−_ T_end_/(QRS × QT) (0.00074 [0.00034–0.00096] vs. 0.00073 [0.00048–0.00012] ms^−1^; *P* = 0.44; Figure 5D).

**Figure 6:**
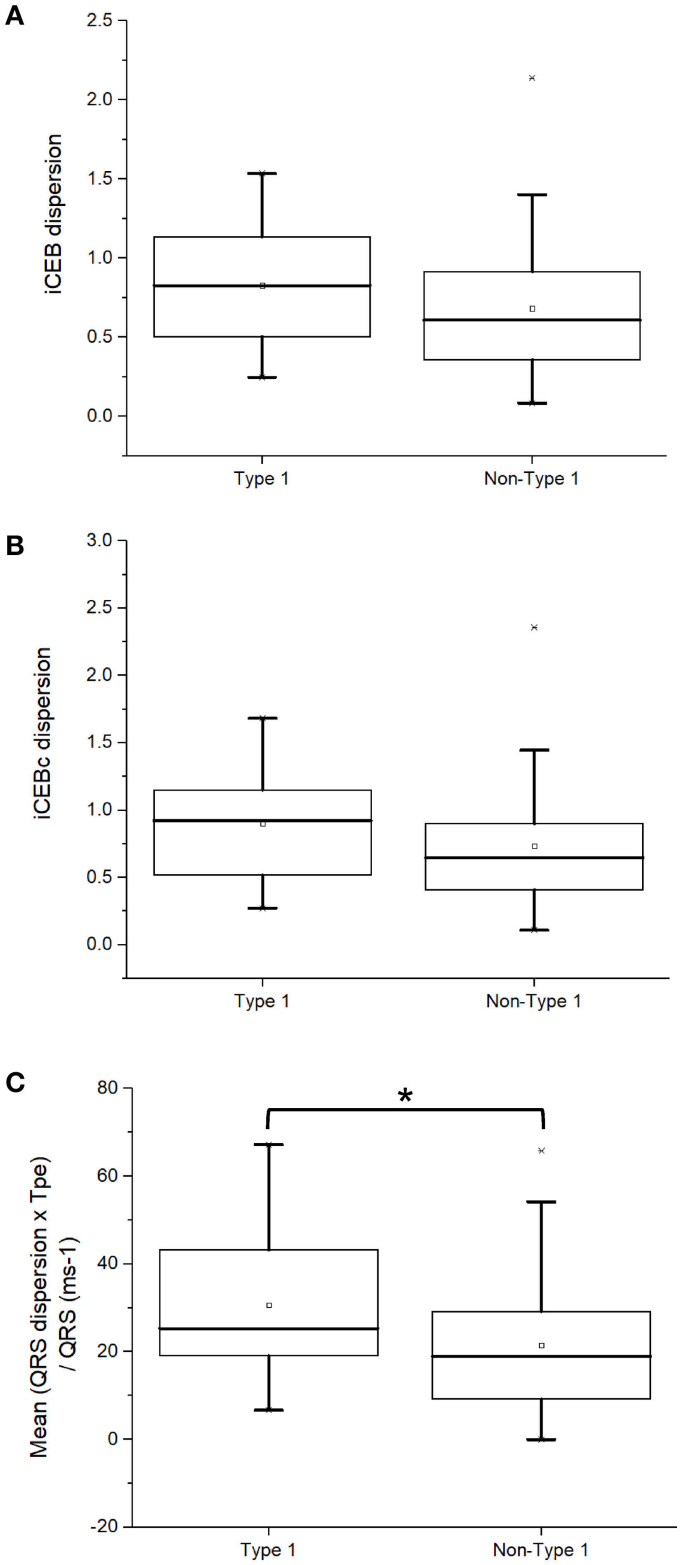
iCEB dispersion **(A)**, iCEBc dispersion **(B)**, or (QRSd × T_peak−_ T_end_)/QRS **(C)** in type 1 and non-type 1 Brugada syndrome patients. *Denotes significant difference between the two groups.

In this study, we calculated dispersion of iCEB and iCEBc for the first time. This is based on the physiological findings that reentrant tachycardia may be due to higher spatial dispersion of excitation wavelength, which can predispose to unidirectional conduction block and reentry (18). Moreover, we quantified (QRSd × T_peak−_ T_end_)/QRS for the first time, a parameter combining both dispersion of conduction and of repolarization. The present analysis found that type 1 BrS patients showed statistically indistinguishable iCEB dispersion (0.83 [0.49–1.14] vs. 0.61 [0.34–0.92]; *P* = 0.09; Figure 6A), iCEBc dispersion (0.93 [0.51–1.15] vs. 0.65 [0.39–0.96]; *P* = 0.08; Figure 6B) but significantly higher mean (QRSd × T_peak−_ T_end_)/QRS (25 [19–44] vs. 19 [9–30] ms; *P* = 0.03; Figure 6C) compared to non-type 1 subjects.

## Discussion

The most important findings of this study are that parameters that measured the dispersion of conduction, repolarization or both processes across the three precordial leads, V1–V3, can distinguish patients with spontaneous type 1 Brugada from those with non-type 1 Brugada patterns. By contrast, the same parameters measured from a single lead only or their mean values were not significantly different between both groups.

Sudden cardiac death (SCD), frequently due to ventricular tachyarrhythmias, is a significant problem globally (19). Patients with Brugada syndrome (BrS) have an increased risk of developing SCD (20, 21). However, it remains difficult to identify patients at the highest risk for developing these arrhythmias (22). Those with a type 1 pattern are thought to have higher risk of developing such adverse events compared with those with non-type 1 patterns (23–26). However, some investigators have reported that those with non-type 1 patterns, which can be converted to a type 1 pattern using drug challenge, are also at higher risks of ventricular arrhythmias (27).

### Depolarization and repolarization hypotheses and their ECG markers

Generally, the mechanism of arrhythmogenesis in BrS have been broadly divided into the depolarization and repolarization hypotheses (12, 28–31). The depolarization hypothesis posits that delayed propagation of action potentials through the right ventricular outflow tract, can lead to reduction of excitation wavelength to induce reentry. By contrast, the repolarization hypothesis posits that differences in repolarization time-course either locally or across the myocardial wall, can create electrotonic currents during phase 2 of the cardiac action potential, leading to reentry (32, 33). It is likely that both mechanisms co-exist and contribute to arrhythmogenesis in BrS.

These findings provide insights into the different electrocardiographic markers that can be used for risk stratification (34, 35). Traditionally, repolarization markers such as QT interval (corrected, QT_c_) have been widely used for this purpose. However they have a low sensitivity and specificity (36), given that ventricular arrhythmias can occur in the presence of a normal or even reduced QT interval (37). By contrast, depolarization or conduction markers such as QRS duration can also predict arrhythmic outcomes in BrS (4, 38).

### Wavelength and dispersion-based markers: traditional and novel indices

Given the limitations of the above markers, recent interests have focused on the role of dispersion-based indices (18). Other markers include QT dispersion (QT_d_) (6, 7), interval from the peak to the end of the T wave (8, 39, 40) [T_peak_ – T_end_, reflecting transmural dispersion of repolarization, TDR (9)], and (T_peak_ – T_end_)/QT ratio (10). These markers stemmed from pre-clinical findings that higher spatial dispersion of repolarization can predispose to phase 2 reentry (41, 42). Although individual studies have reported the value for risk stratification, a recent study of 448 patients, which is the largest cohort to date, found no difference in this interval between subjects with VF/SCD and those who were asymptomatic (43). By contrast, QRS dispersion reflects spatial dispersion of CVs, increases in which can lead to unidirectional conduction block and reentry (44). Higher QRS dispersion (5) and increased fragmentation of the QRS complex (45, 46), have been associated with pro-arrhythmic outcomes in BrS patients.

Experiments from animal studies have demonstrated the importance of excitation wavelength, λ, given by the product of CV and refractory period, in determining arrhythmogenicity (47, 48). Thus, a decrease in either parameter reduces the length of the excitation wave, meaning that a higher number of re-entrant circuits can be accommodated in a given volume of myocardial tissue. However, λ must be determined by invasively with electrophysiological testing (49). This prompted Lu and colleagues to propose iCEB, the first electrocardiographic marker that serves as a good approximate of λ (14). This was subsequently shown to be decreased in BrS patients (15). Our study extends these findings by demonstrating that iCEB and iCEBc were similar between type 1 and non-type 1 BrS patients.

Given the observations that dispersion-based markers could provide additional value for arrhythmic risk stratification (36, 50), a number of indices incorporating repolarization and conduction dispersion have been proposed, namely T_peak_ – T_end_/QRS, T_peak_ – T_end_/(QT × QRS) and QRS_d_ × T_peak_ – T_end_/QRS (16, 17). Recently, Robyns and colleagues found that T_peak_ – T_end_/QRS or T_peak_ – T_end_/(QT × QRS), like iCEB, were significantly different between control, BrS and long QT syndrome patients (51). However, data from Germany found no difference in either index between asymptomatic and symptomatic BrS patients (52). In our study, we found that both parameters did not significantly differ between type 1 and non-type 1 BrS patients. By contrast, we found significantly higher mean QRS_d_ × T_peak_ – T_end_/QRS but similar iCEB and iCEBc dispersion parameters in type 1 compared to non-type 1 BrS patients. These findings therefore provide the evidence that higher dispersion of repolarization and conduction are found in type 1 BrS patients, which can potentially explain the higher incidence of ventricular arrhythmias and SCD than non-type 1 patients.

### Limitations

Several limitations of this study are recognized. Firstly, this included a small cohort from a single center. These findings therefore need to be explored in larger cohorts. Secondly, this was a retrospective study that did not examine hard endpoints such as arrhythmic or mortality outcomes. It should be noted that our work is hypothesis-generating. Future studies can explore whether these novel dispersion-based electrocardiographic markers are useful for risk stratification in terms of arrhythmic or mortality outcomes.

## Conclusions

This study provides electrocardiographic evidence that higher levels of dispersion in conduction and repolarization are found in type 1 than non-type 1 BrS patients. This may potentially explain the higher incidence of ventricular arrhythmias in the former group. Indices reflecting cumulative conduction and repolarization abnormalities may provide additional value for risk stratification.

## Author contributions

GT: study conception, data acquisition, data analysis, statistical analysis, data interpretation, drafting of manuscript, critical revision of manuscript, creation of figures; response to reviewer comments. KHCL: data acquisition. WKKW and KPL: study conception and supervision. YX: revision of manuscript and response to reviewer comments. All authors: data analysis and interpretation, critical revision of manuscript.

### Conflict of interest statement

The authors declare that the research was conducted in the absence of any commercial or financial relationships that could be construed as a potential conflict of interest.
