# Supplementary material for: Higher Dispersion Measures of Conduction and Repolarization in Type 1 Compared to Non-type 1 Brugada Syndrome Patients: An Electrocardiographic Study From a Single Center
Source: Front Cardiovasc Med. 2018 Oct 4;5:132. doi: 10.3389/fcvm.2018.00132 (PMC6180153; doi:10.3389/fcvm.2018.00132)
Supplement: Supplementary file 1 [file Data_Sheet_1.PDF]

26222

01-Aug-2009 16:26:16

PageWrite

Rate	105	. AGE NOT ENTERED, ASSUMED TO BE 50 YEARS FOR PURPOSE OF ECG INTERPRETATION
PR	205	. SINUS TACHYCARDIA, RATE 105............................normal P axis, rate>=100
QRSD	88	. BORDERLINE AV CONDUCTION DELAY.....................PR>195 age 16-60 rate 91-120
QT	307	
QTc	406	

--Axis--

P	51
QRS	32
T	39

- BORDERLINE ECG -

Unconfirmed diagnosis.

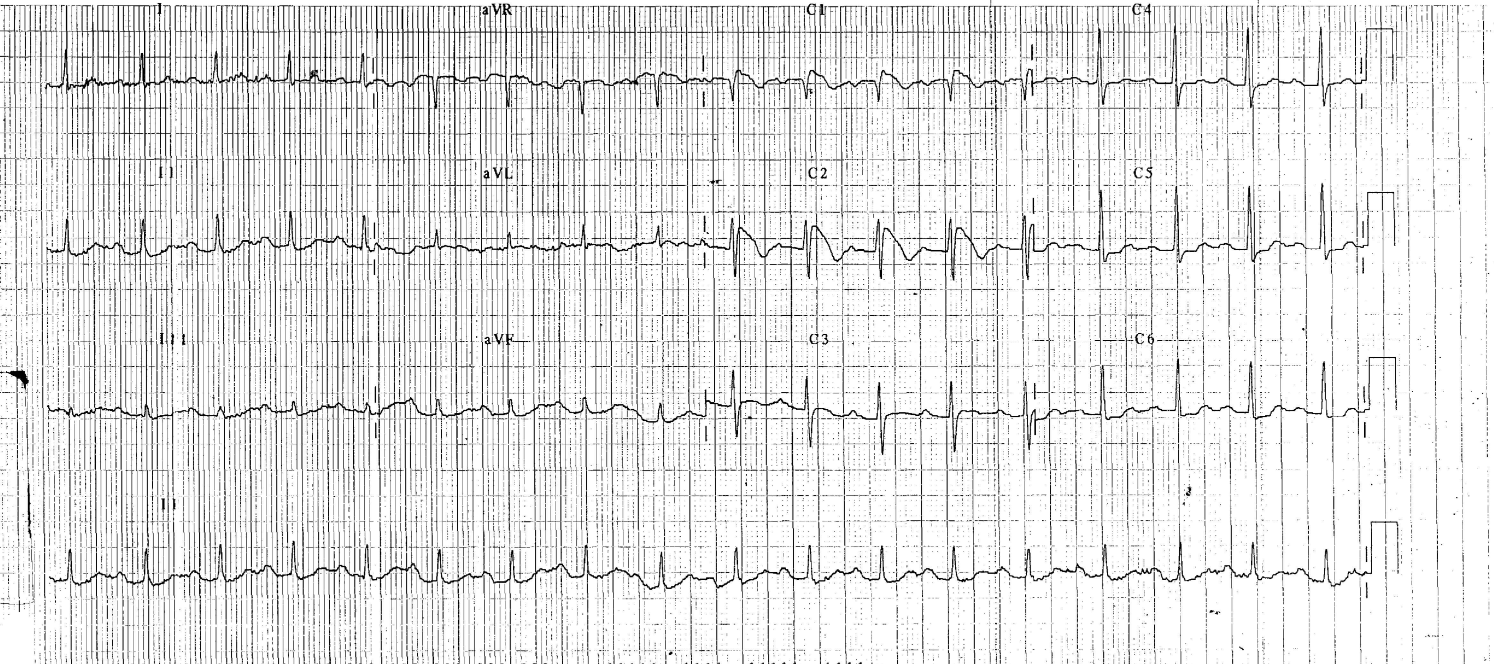

000-000-000 000-000-000-000-000-000 000 11111--1111--11111--1111
065-065-062 055-056-055-055-056-055 2134@64
A 07 07 4350 23.6°C 6.17V 26.17V Battery OK 25 mm/s 10 mm/mV F ∿ 0.5 Hz - 40 Hz W HP709 58877

ID: 1722

3rd Female Years

HR	:	67	bpm
P	:	122	ms
PR	:	201	ms
QRS	:	85	ms
QT/QTc	:	353/374	ms
P/QRS/T	:	70/22/75	°
RV5/SV1	:	0.499/0.488	mV

03/4 10/8/2017

Diagnosis Information:

Sinus Rhythm
Abnormal Q Wave(V6)
Low T Wave(V4)
Inverted T Wave(V2)
Marked ST Depression(V6)
Middle ST Elevation(V2)

Report Confirmed by:

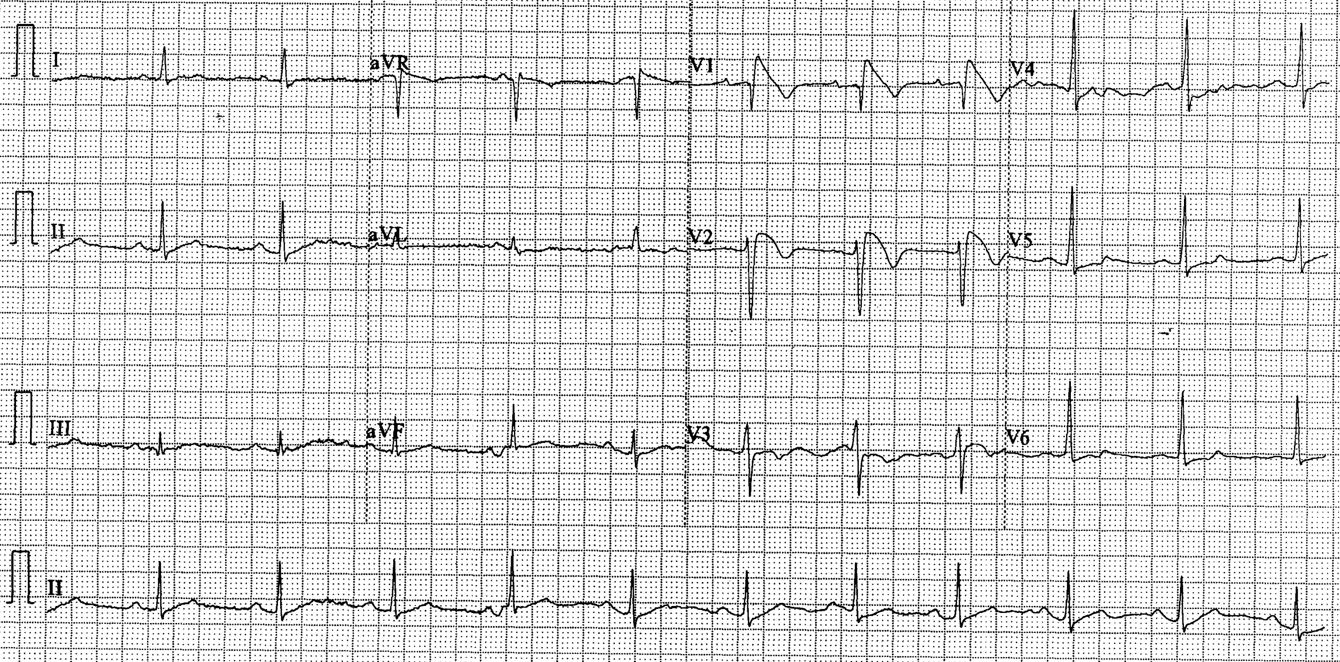

0.25~25Hz AC50 25mm/s 10mm/mV 4*2.5s+1r ♥67 SE-1200Express V2.01 SEMIP V1.7

(A&F

35003

22-05-2016 16:05:20

ID
Name
Age

HR	: 85	BPM
P Dur	: 123	ms
PR int	: 184	ms
QRS Dur	: 108	ms
QT/QTC int	: 355/423	ms
P/QRS/T axis	: 30/115/28	°
RV5/SV1 amp	: 1.236/0.093	mV
RV5+SV1 amp	: 1.329	mV
RV6/SV2 amp	: 0.979/0.796	mV

Diagnosis Information:
800: Sinus Rhythm
711: Abnormal Q Wave(III,aVF)
621: Inverted T Wave(V2)
671: Slight ST Elevation(V1,V2)
203: Right Axis Deviation

Unconfirmed Report.

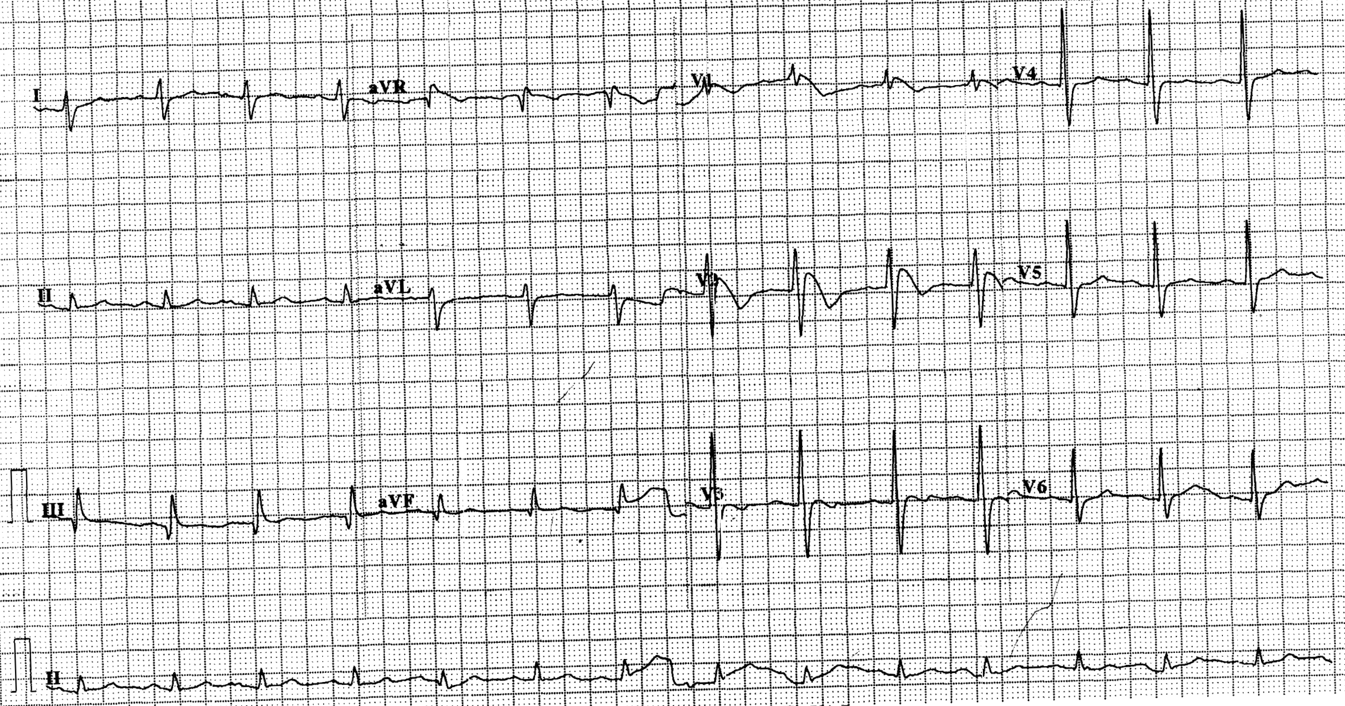

28222

28-Feb-2010 16:15:29

Rate	85	. Normal sinus rhythm, rate 85....................Normal P axis, PR, rate & rhythm
PR	186	. Left anterior fascicular block...................QRS axis -45 deg., I:40 inferior
QRSD	93	. Early transition with RSR' in V1 or V2...........QRS positive and R' in V1 or V2
QT	367	. Left atrial enlargement..............................P' -.10 mV and 40 mS in V1
QTc	436	. Anterolateral ST elevation............................ST > .15 mV I,aVL,V2-V6

--Axis--

P	71
QRS	-85
T	64

- ABNORMAL ECG -

Unconfirmed diagnosis.

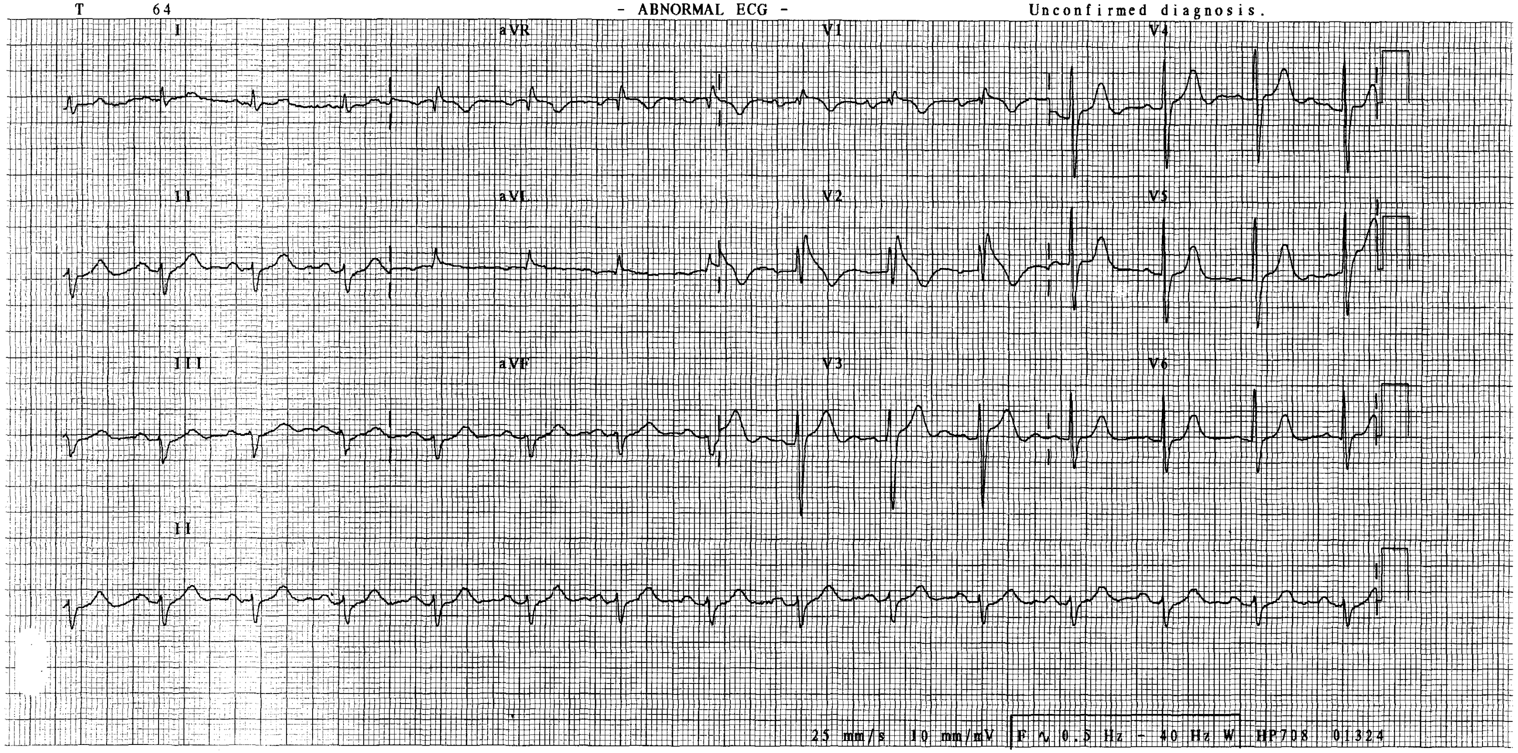

12011

HR	:	74	BPM
P Dur	:	133	ms
PR int	:	181	ms
QRS Dur	:	120	ms
QT/QTC int	:	440/489	ms
P/QRS/T axis	:	46/5/3	°
RV5/SV1 amp	:	1.284/0.420	mV
RV5+SV1 amp	:	1.704	mV
RV6/SV2 amp	:	0.876/0.837	mV

Diagnosis Information:
800: Sinus Rhythm
141: QT Interval Prolongation

Unconfirmed Report.

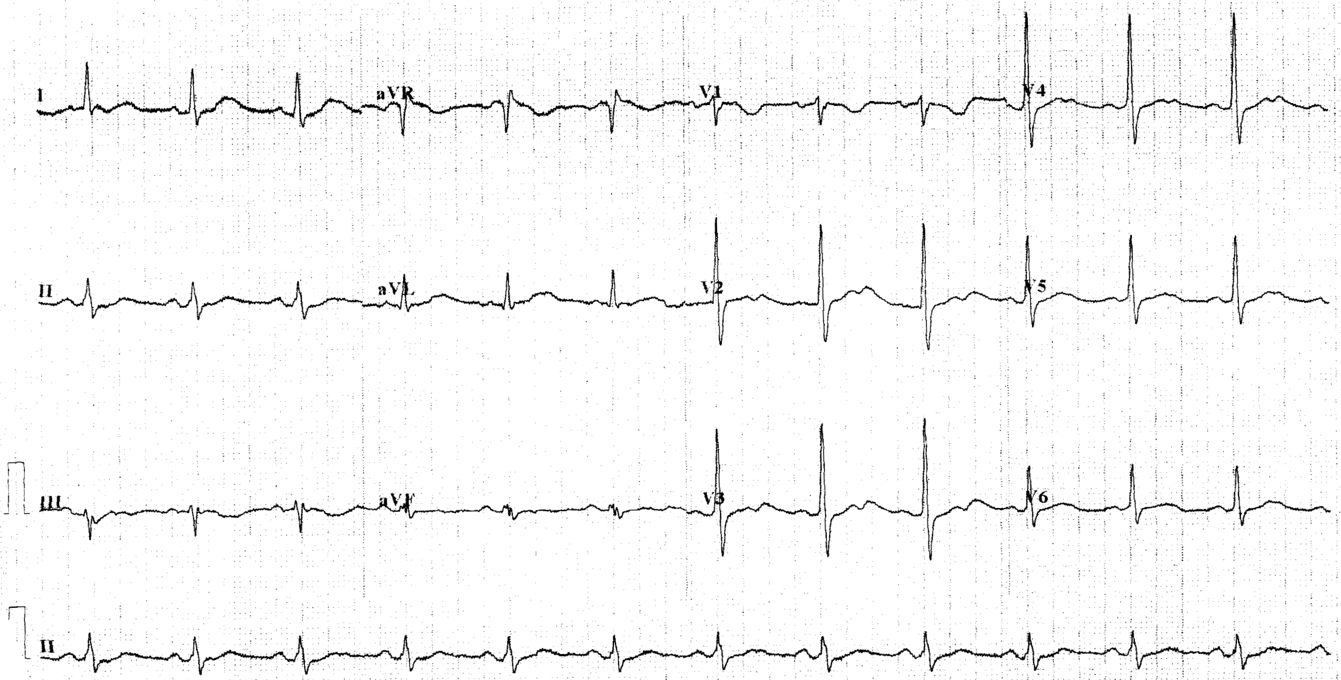

0.67~25Hz AC50 25mm/s 10mm/mV 4*2.5s+1r SE-1200Express V1.824 SEMIP V1.7

61872

ID:
Name:
Sex: Birth Date: Years
cm kg mmHg
Medication:
Symptoms:
History:

Apr-17-2010 3:12 PM

1100 Sinus rhythm
1470 with occasional supraventricular premature complexes
2320 Nonspecific intraventricular conduction delay
4436 Possible septal injury or acute infarct
7102 Moderate right axis deviation
9150 ** abnormal ECG **

Vent rate	91	bpm
PR int	132	ms
QRS dur	112	ms
QT/QTc int	382/ 431	ms
P/QRS/T axis	60/ 93/ 51	°
RV5/SV1 amp	0.730/ 0.760	mV
RV5+SV1 amp	1.490	mV

Unconfirmed Report
Reviewed by:

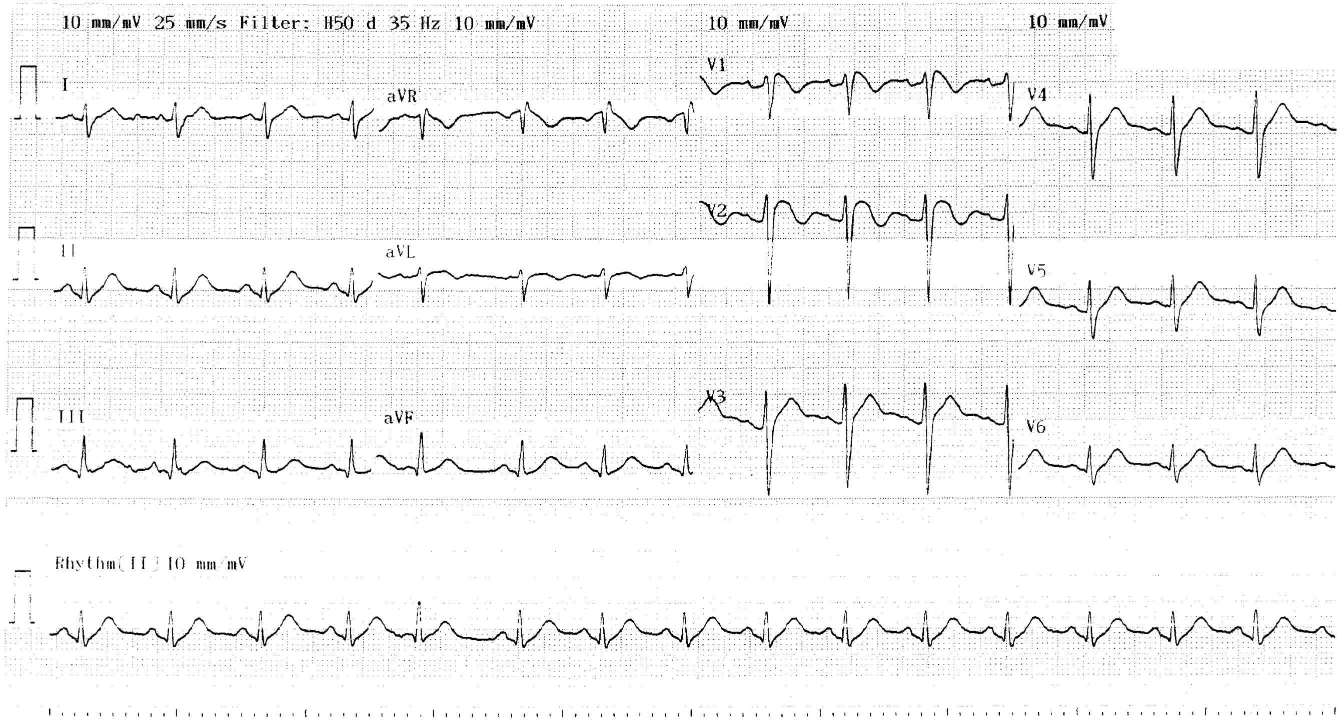

1350K 03-01 02-52 Dept.: Exam:UCH AandE

61872

09-03-2015 23:27:32 For Use On EDAN 01.57.32462 CE

0278120

ID : E3052676
Name :
Age : 65 Years Gender : Male

HR	: 73	BPM
P Dur	: 115	ms
PR int	: 172	ms
QRS Dur	: 112	ms
QT/QTC int	: 400/441	ms
P/QRS/T axis	: 82/100/16	°
RV5/SV1 amp	: 0.955/0.348	mV
RV5+SV1 amp	: 1.303	mV
RV6/SV2 amp	: 0.846/0.605	mV

Report Confirmed by:

Diagnosis Information:
800: Sinus Rhythm
501: Incomplete Right Bundle Branch Block
672: Middle ST Elevation(V1)
203: Right Axis Deviation

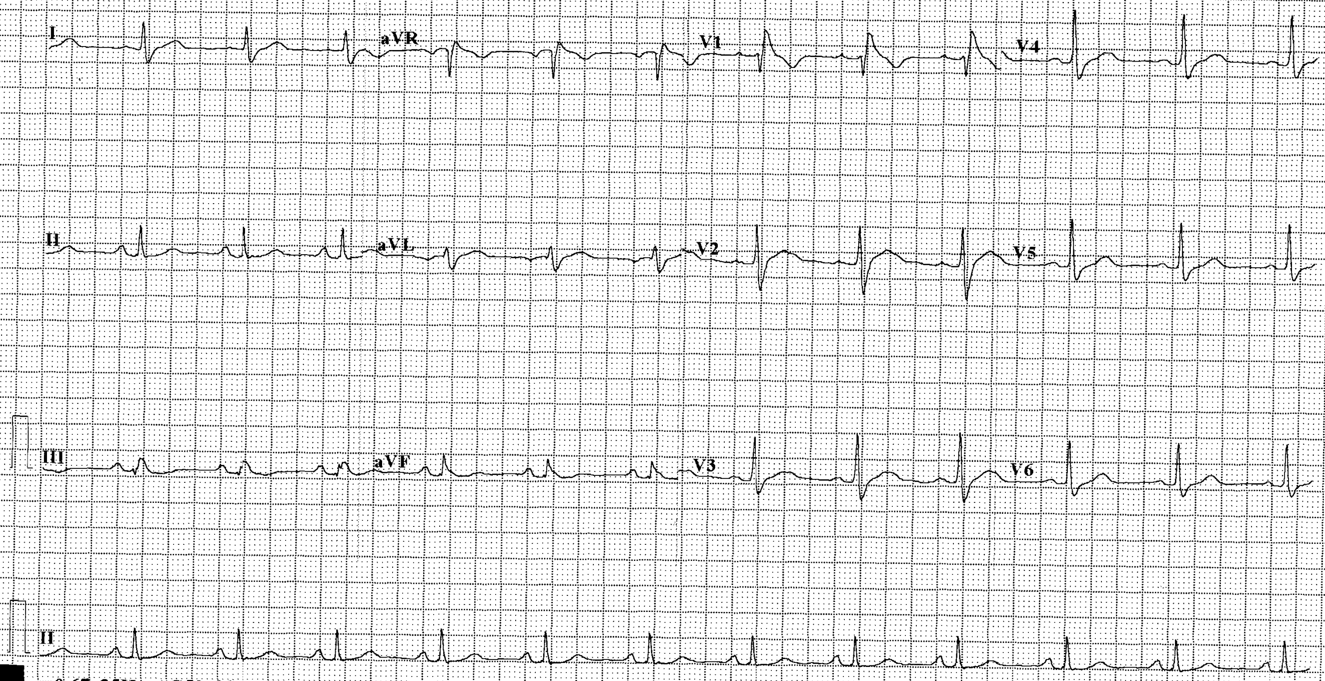

0.67~25Hz AC50 25mm/s 10mm/mV 4*2.5s+1r SE-1200Express V1.823 SEMIP V1.7

64006

22-Nov-2010 08:40:05

Rate	67	. AGE NOT ENTERED, ASSUMED TO BE 50 YEARS FOR PURPOSE OF ECG INTERPRETATION
PR	221	. SINUS RHYTHM, RATE 67......................................normal P axis, rate
QRSD	130	. FIRST DEGREE AV BLOCK..................................PR>210 age 16-60 rate 51- 90
QT	404	. RBBB AND LAFB...RBBB, QRS axis(-40,240)
QTc	426	

--Axis--

P	50
QRS	-57
T	23

- ABNORMAL ECG -

Unconfirmed diagnosis.

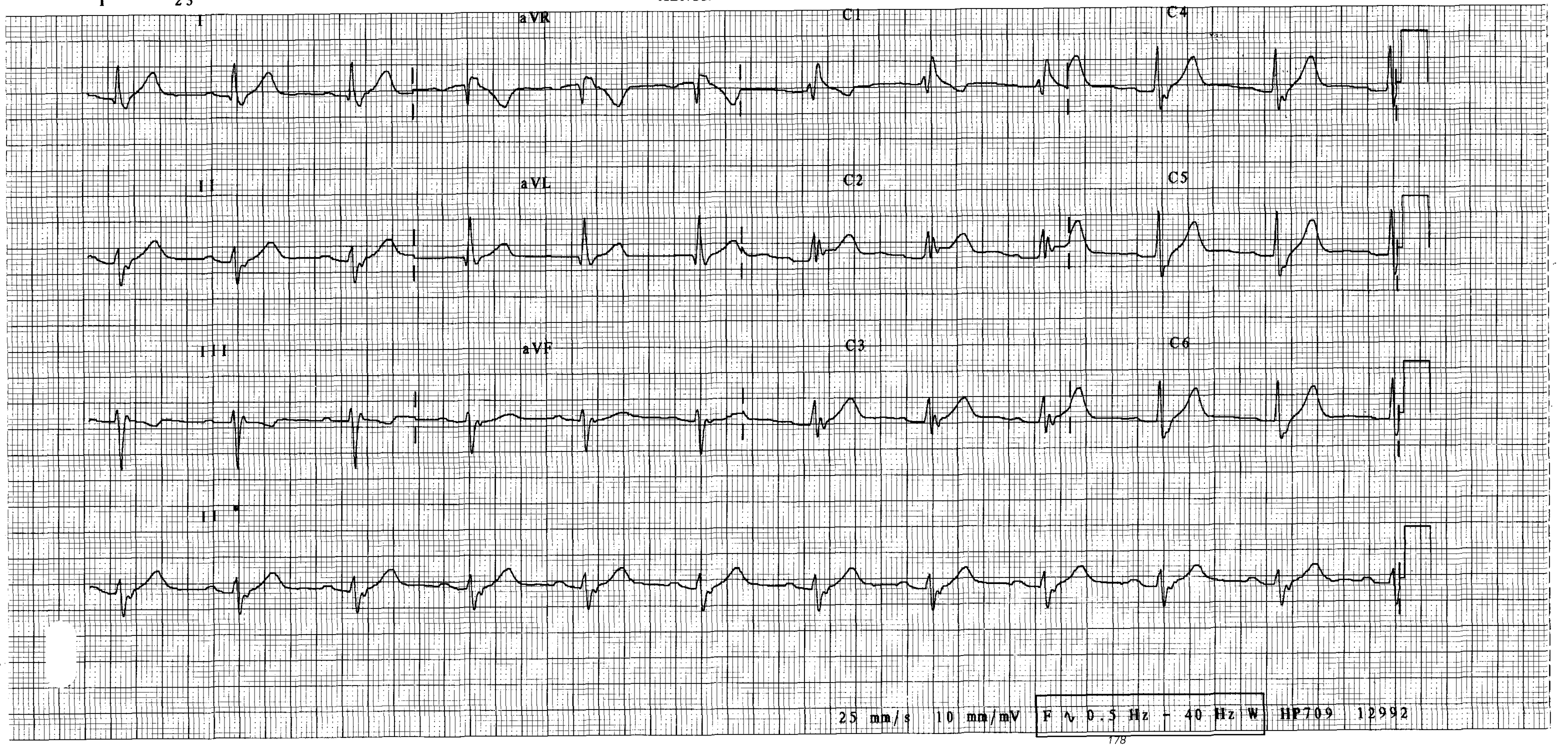

26222

22-04-2018 17:54:06
ID :
Name :

HR	: 88	BPM	
P Dur	: 143	ms	
PR int	: 204	ms	
QRS Dur	: 139	ms	
QT/QTC int	: 388/470		ms
P/QRS/T axis	: 74/177/66		°
RV5/SV1 amp	: 1.088/0.068		mV
RV5+SV1 amp	: 1.156		mV
RV6/SV2 amp	: 0.375/0.110		mV

Diagnosis Information:
800: Sinus Rhythm
504: Complete Right Bundle Branch Block
701: Poor R Wave Progression(V2)
621: Inverted T Wave(V2,V3)
651: High T wave(V5)
661: Slight ST Depression(V3,V4)
671: Slight ST Elevation(II,III)
306: Right Ventricular Hypertrophy

Unconfirmed Report.

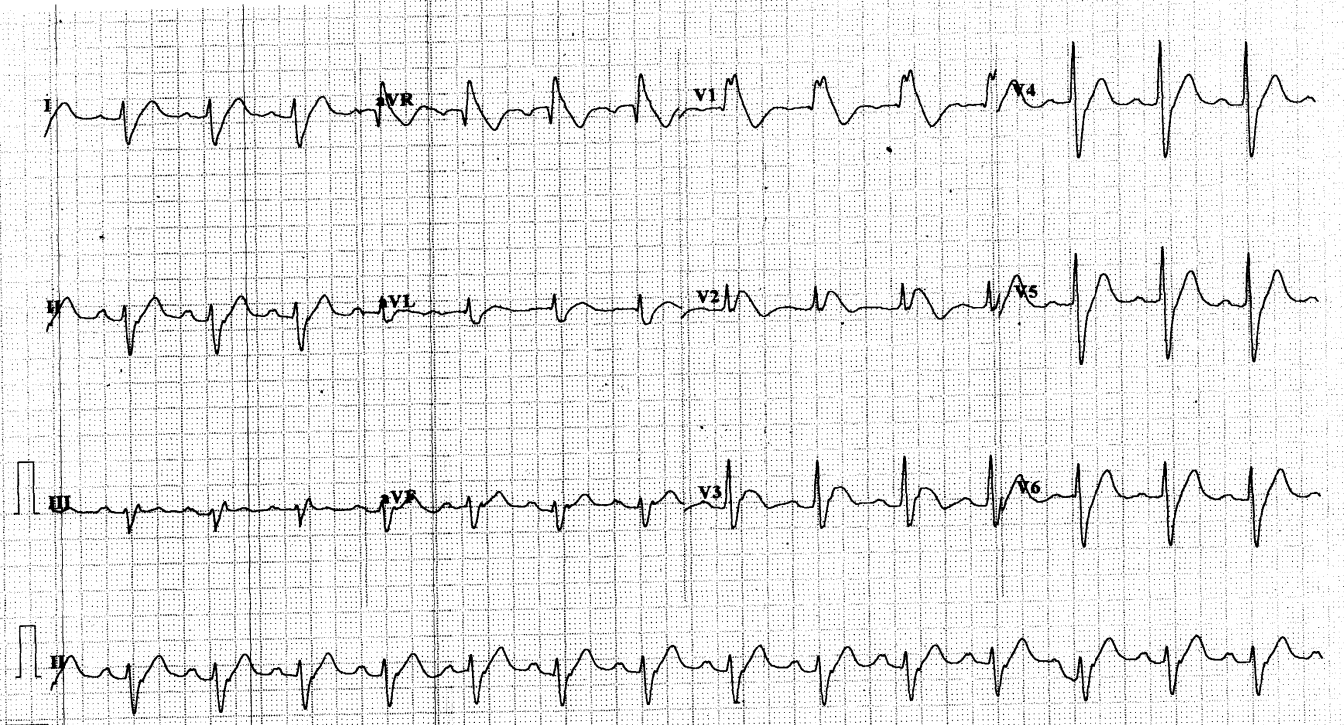

0.67~25Hz AC50 25mm/s 10mm/mV 4*2.5s+1r SE-1200Express V1.824 SEMIP V1.7

61872

ID:
Name:
Sex: Birth Date: Years
cm kg mmHg
Medication:
Symptoms:
History:

Jan-30-2012 9:49 PM

1100 Sinus rhythm
2440 Incomplete right bundle branch block
2630 Left anterior fascicular block
4136 Possible anterior injury or acute infarct
9150 ** abnormal ECG **

Vent rate	78	bpm
PR int	182	ms
QRS dur	116	ms
QT/QTc int	392/ 426	ms
P/QRS/T axis	43/ -78/ 65	°
RV5/SV1 amp	1.380/ 0.205	mV
RV5+SV1 amp	1.585	mV

Unconfirmed Report
Reviewed by:

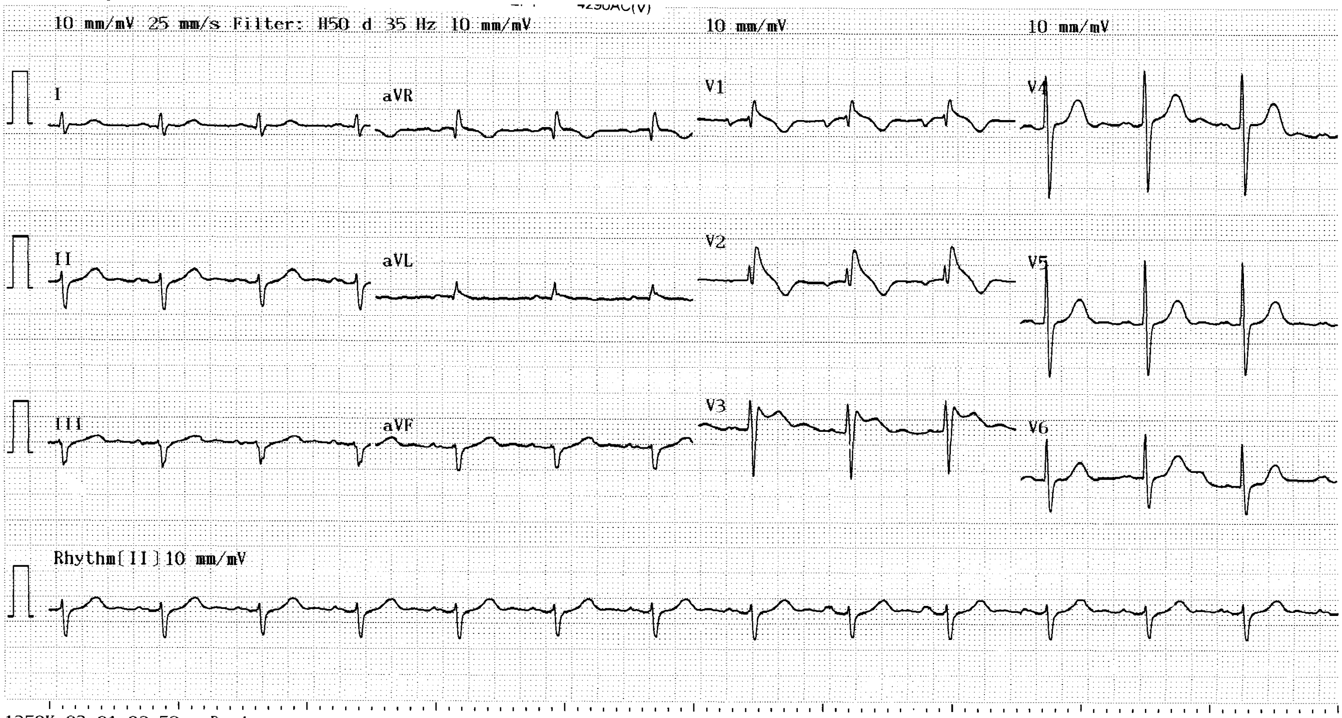

1350K 03-01 02-52 Dept.:

Exam:UCH AandE

61872

09-Apr-2011 22:31:00

Rate	108	. AGE NOT ENTERED, ASSUMED TO BE 50 YEARS FOR PURPOSE OF ECG INTERPRETATION
PR	178	. SINUS ARRHYTHMIA, VARIED RATE 93-143....................varied V-rate, mean>=100
QRSD	101	. BORDERLINE LEFT ATRIAL ABNORMALITY............................P>30mS, <-.10mV V1
QT	306	. INCOMPLETE RIGHT BUNDLE BRANCH BLOCK...............QRS>100, terminal axis(90,270)
QTc	410	

--Axis--

P	70
QRS	69
T	-29

- ABNORMAL ECG -

Unconfirmed diagnosis.

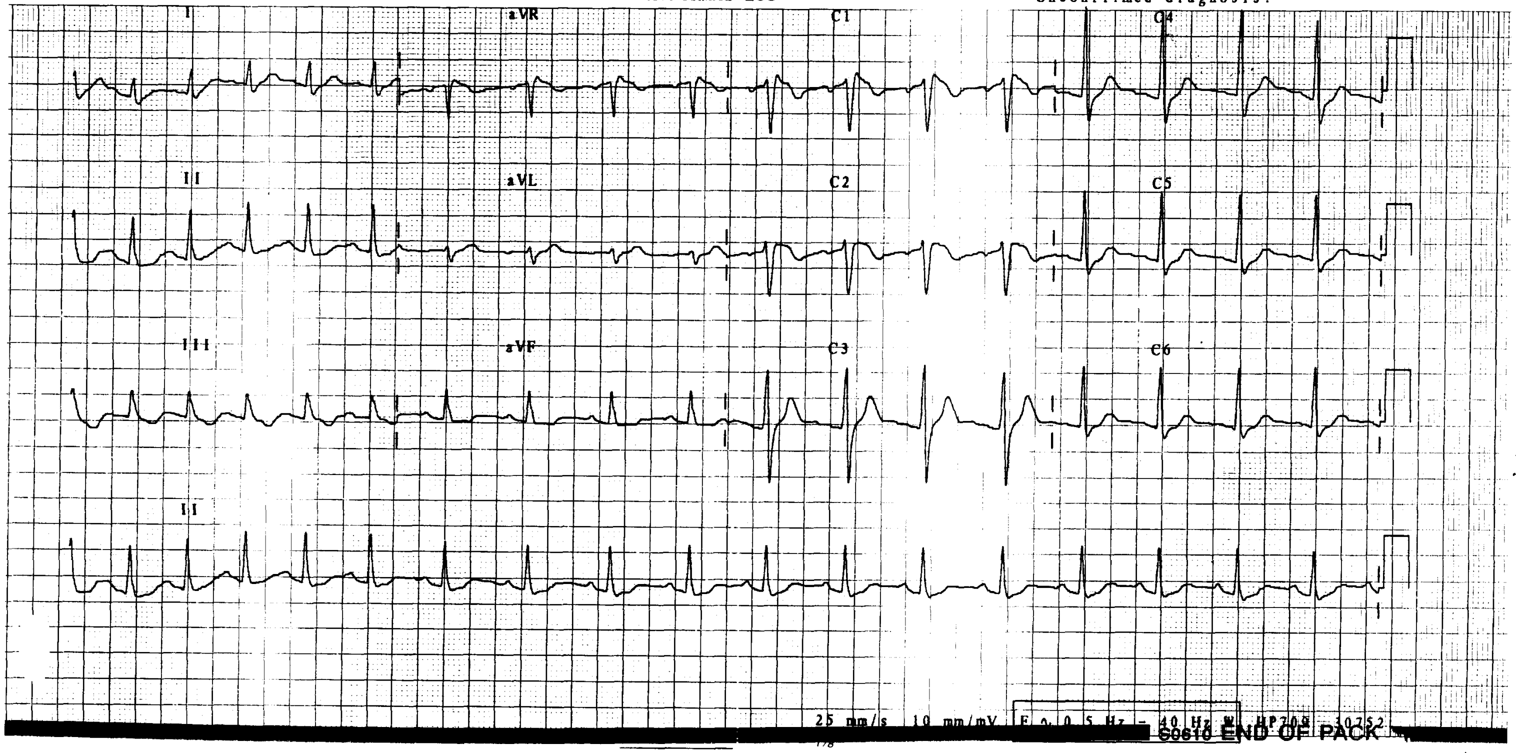

26222

[illegible]

ID:

Years

HR	: 76	bpm
P	: 130	ms
PR	: 194	ms
QRS	: 111	ms
QT/QTc	: 368/416	ms
P/QRS/T	: 64/19/25	°
RV5/SV1	: 1.728/0.312	mV

Diagnosis Information:
Sinus Rhythm
Incomplete Right Bundle Branch Block
Slight ST Elevation(V2,V3,V4)

Unconfirmed Report.

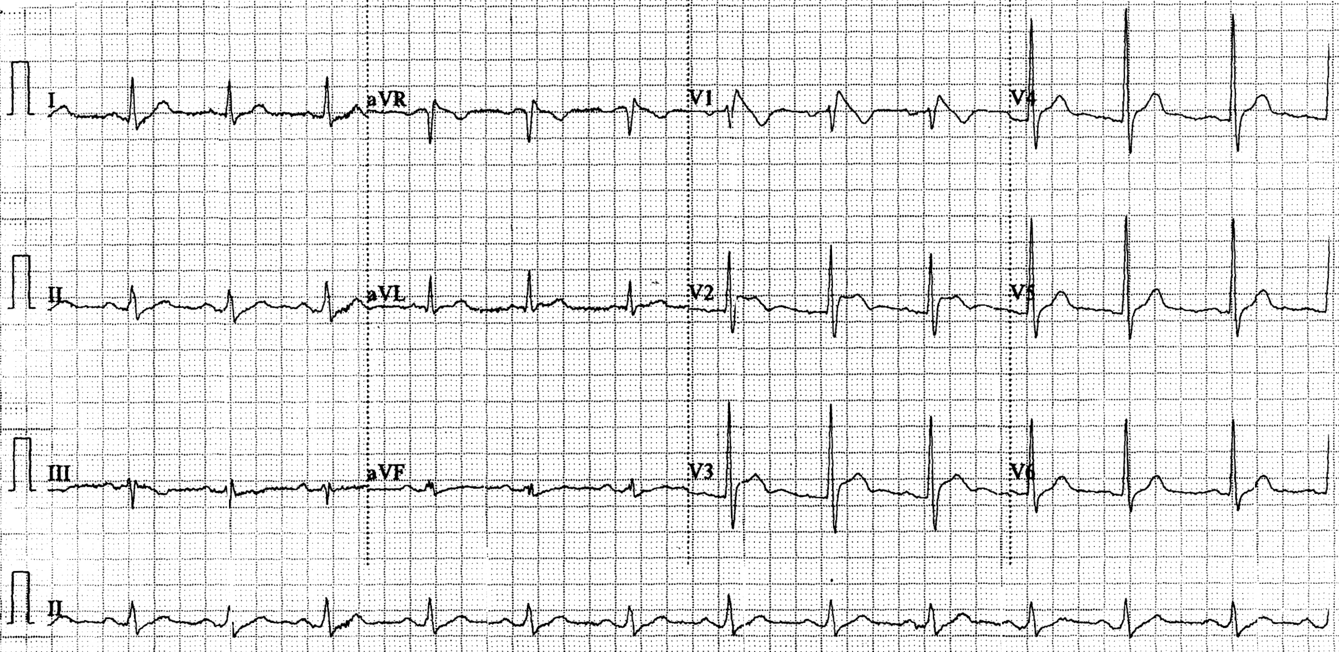

0.67~25Hz AC50 25mm/s 10mm/mV 4*2.5s+1r ♥76 SE-1200Express V2.01 SEMIP V1.7

ID: [illegible] 05.10.2013 19:28:47

D.O.B.:
Meds:
Class:
Dr:
Tech:

Comment:

Vent. Rate:	78 bpm
RR Interval:	768 ms
PR Interval:	138 ms
QRS Duration:	142 ms
QT Interval:	370 ms
QTc Interval:	401 ms
QT Dispersion:	24 ms
P Axis:	52 deg
QRS Axis:	64 deg
T Axis:	60 deg

— Warning: Data quality may affect interpretation —
*** CONSIDER ACUTE STEMI ***
Sinus arrhythmia.
Right bundle branch block
Anteroseptal ST elevation, CONSIDER ACUTE INFARCT

Abnormal ECG

* Unconfirmed Analysis *

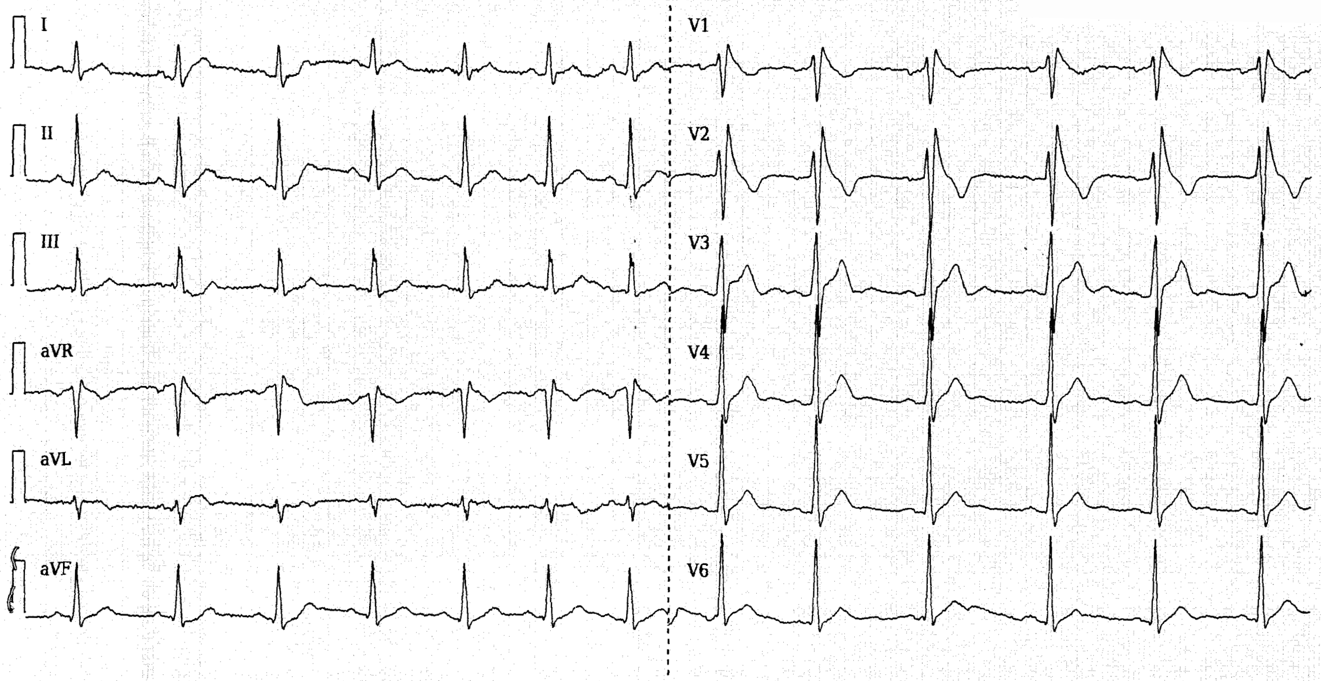

L: 10 mm/mV
C: 10 mm/mV
QTc: Hodges
KWH A&E1
Serial #:E8500-002335
25 mm/s
~STABLE 40 Hz

25005

08-05-2016 10:51:35

ID
Name
Age

HR	:	79	BPM
P Dur	:	109	ms
PR int	:	163	ms
QRS Dur	:	111	ms
QT/QTC int	:	357/410	ms
P/QRS/T axis	:	52/39/38	°
RV5/SV1 amp	:	1.539/0.246	mV
RV5+SV1 amp	:	1.785	mV
RV6/SV2 amp	:	1.281/0.547	mV

Unconfirmed Report.

Diagnosis Information:
800: Sinus Rhythm
501: Incomplete Right Bundle Branch Block
671: Slight ST Elevation(V4)

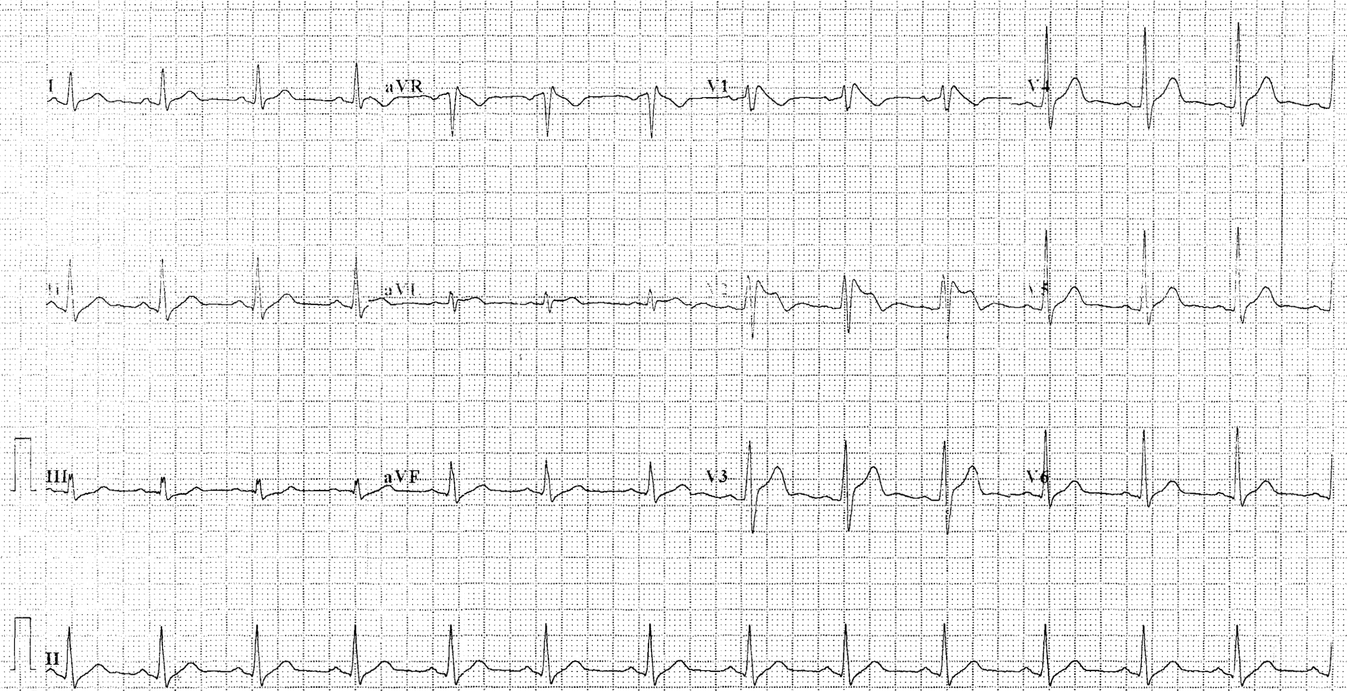

[redacted] 0.67~25Hz AC50 25mm/s 10mm/mV 4*2.5s+1r SE-1200Express V1.824 SEMIP V1.7

23006

0049109

Name :
Age : 56 Years Gender : Male

HR	:	75	BPM
P Dur	:	107	ms
PR int	:	144	ms
QRS Dur	:	105	ms
QT/QTC int	:	367/410	ms
P/QRS/T axis	:	77/78/70	°
RV5/SV1 amp	:	2.812/0.931	mV
RV5+SV1 amp	:	3.743	mV
RV6/SV2 amp	:	1.836/1.317	mV

Unconfirmed Report.

Diagnosis Information:
800: Sinus Rhythm
621: Inverted T Wave(V2)
671: Slight ST Elevation(V4)
301: High Voltage(Left Ventricle)

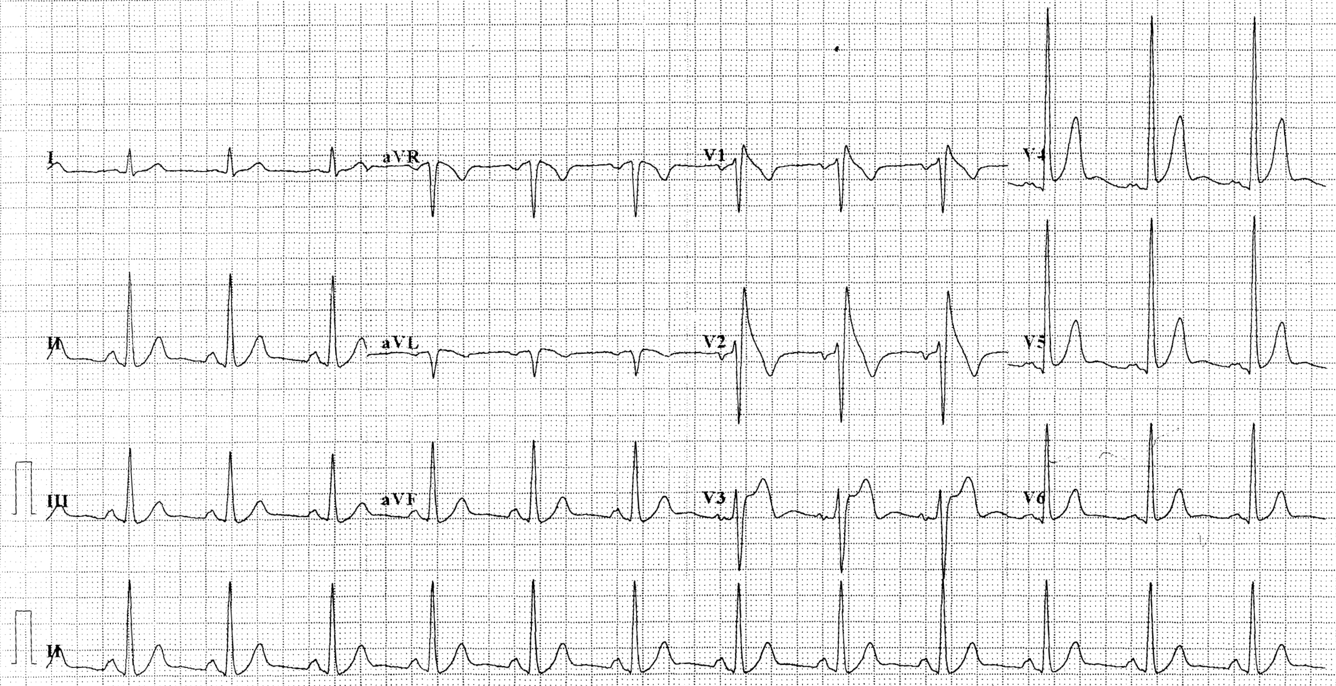

0.67~25Hz AC50 25mm/s 10mm/mV 4*2.5s+1r SE-1200Express V1.824 SEMIP V1.7

64006

02-03-2017 08:29:24
ID:

Years

HR	: 88	bpm
P	: 115	ms
PR	: 174	ms
QRS	: 108	ms
QT/QTc	: 369/448	ms
P/QRS/T	: 52/37/67	°
RV5/SV1	: 0.733/0.290	mV

Diagnosis Information:
Sinus Rhythm
Incomplete Right Bundle Branch Block
Inverted T Wave(V2)
Slight ST Elevation(V1)

Unconfirmed Report.

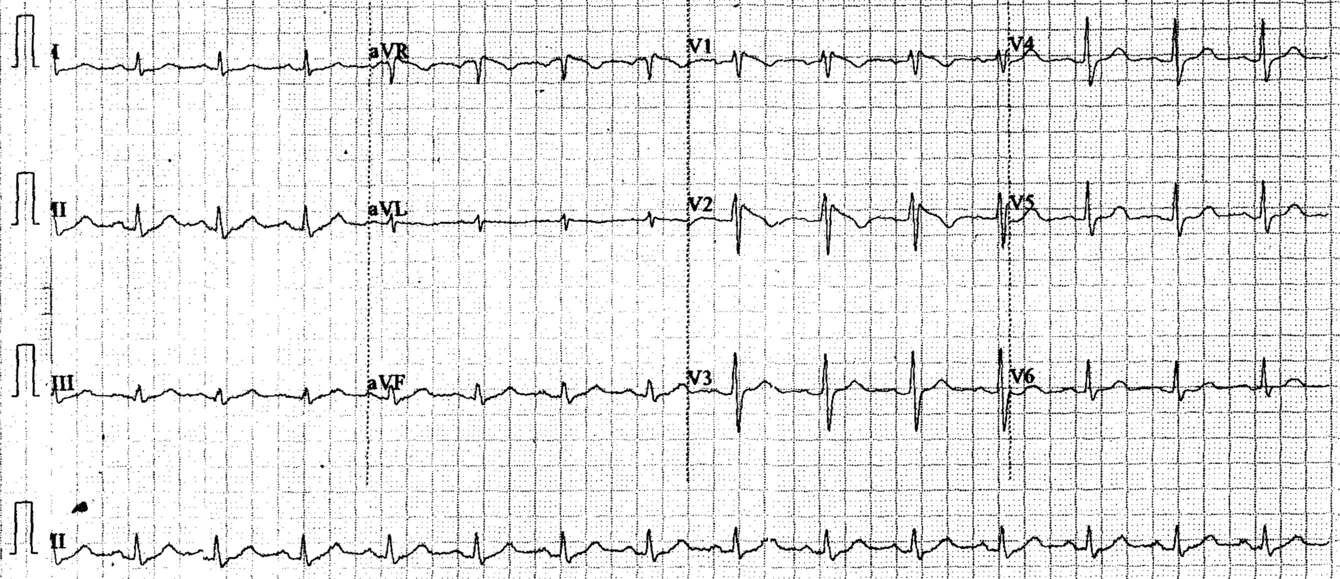

0.67-25Hz AC:0 25mm/s 10mm/mV 4*2.5s+1r ♥88 SE-1200Express V2.01 SEMIP V1.7

26222

1[illegible]-0[illegible]-201[illegible] 1[illegible]:44:2[illegible]

084075

ID :
Name :
Age : Years

HR	:	61	BPM
P Dur	:	117	ms
PR int	:	228	ms
QRS Dur	:	136	ms
QT/QTC int	:	469/475	ms
P/QRS/T axis	:	58/122/30	°
RV5/SV1 amp	:	1.216/0.085	mV
RV5+SV1 amp	:	1.301	mV
RV6/SV2 amp	:	0.921/0.000	mV

Diagnosis Information:
800: Sinus Rhythm
410: First-degree Atrioventricular Block
504: Complete Right Bundle Branch Block
522: Suspect Bifascicular Bundle Block
204: Marked Right Axis Deviation

Report Confirmed by:

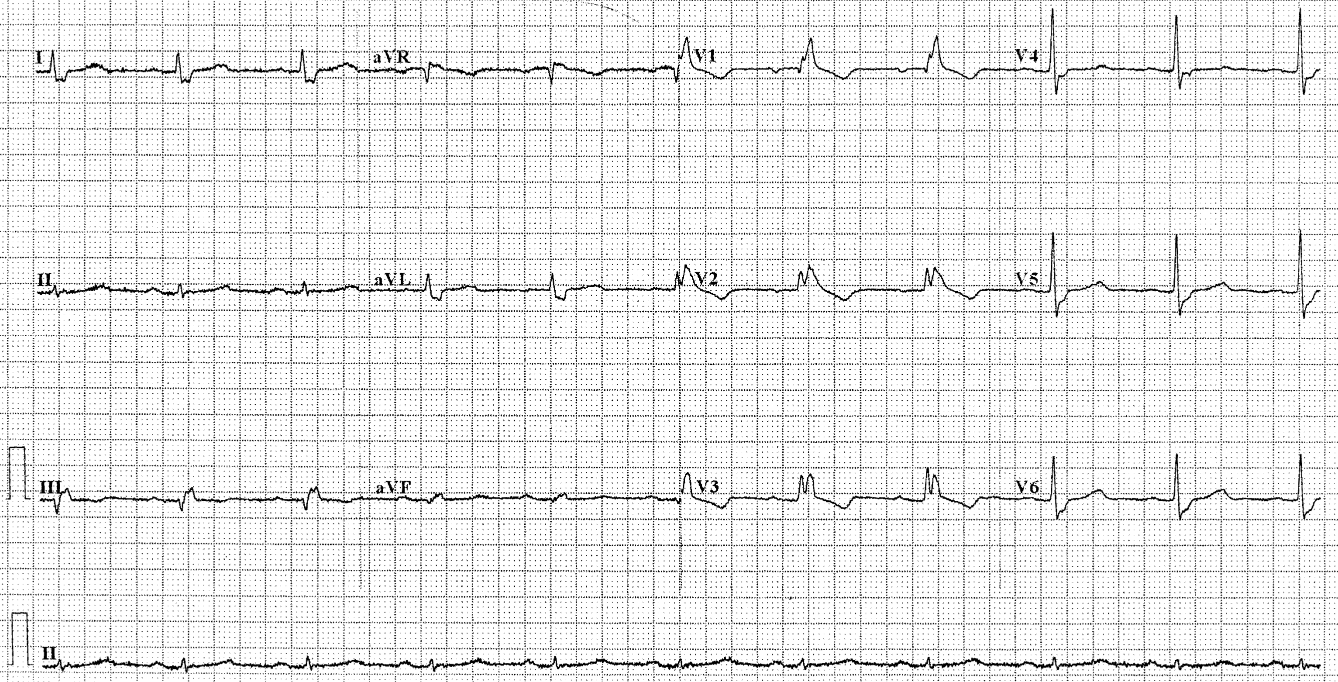

0.67~45Hz AC50 25mm/s 10mm/mV 4*2.5s+1r SE-12Express V1.82 SEMIP V1.5

12011

12-Aug-2011 08:12:49

2

Rate	64	. AGE NOT ENTERED, ASSUMED TO BE 50 YEARS FOR PURPOSE OF ECG INTERPRETATION
PR	154	. NORMAL SINUS RHYTHM, RATE 64....................normal P axis, PR, rate & rhythm
QRSD	110	. INCOMPLETE RIGHT BUNDLE BRANCH BLOCK..............QRS>100, terminal axis(90,270)
QT	406	
QTc	419	

--Axis--

P	81
QRS	87
T	77

- ABNORMAL ECG -

Unconfirmed diagnosis.

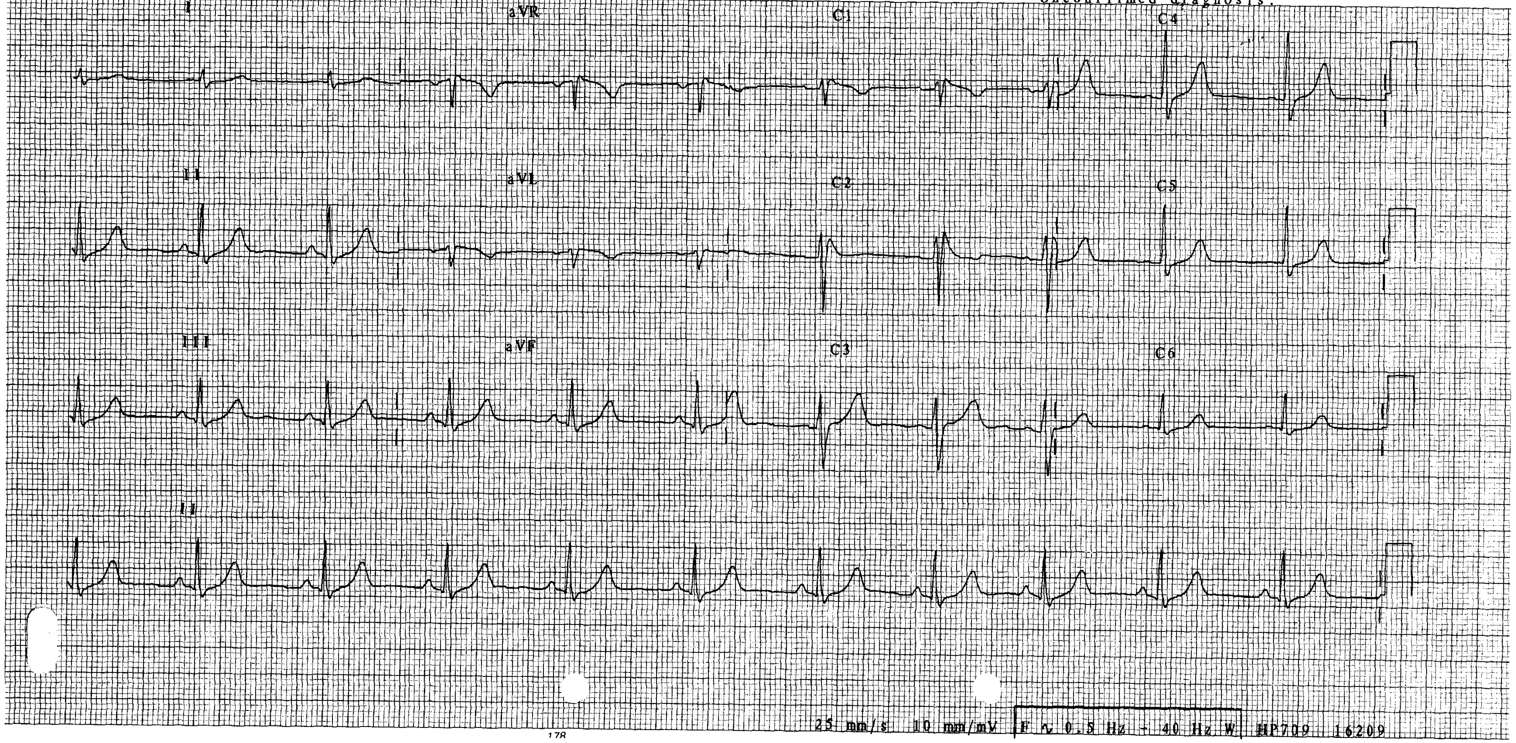

178

26222

30-06-2017 23:25:46

ID :
Name :
Age

HR	:	69	BPM
P Dur	:	118	ms
PR int	:	178	ms
QRS Dur	:	91	ms
QT/QTC int	:	393/424	ms
P/QRS/T axis	:	77/82/42	°
RV5/SV1 amp	:	1.138/0.617	mV
RV5+SV1 amp	:	1.755	mV
RV6/SV2 amp	:	0.964/0.952	mV

Unconfirmed Report.

Diagnosis Information:
800: Sinus Rhythm
671: Slight ST Elevation(V1)

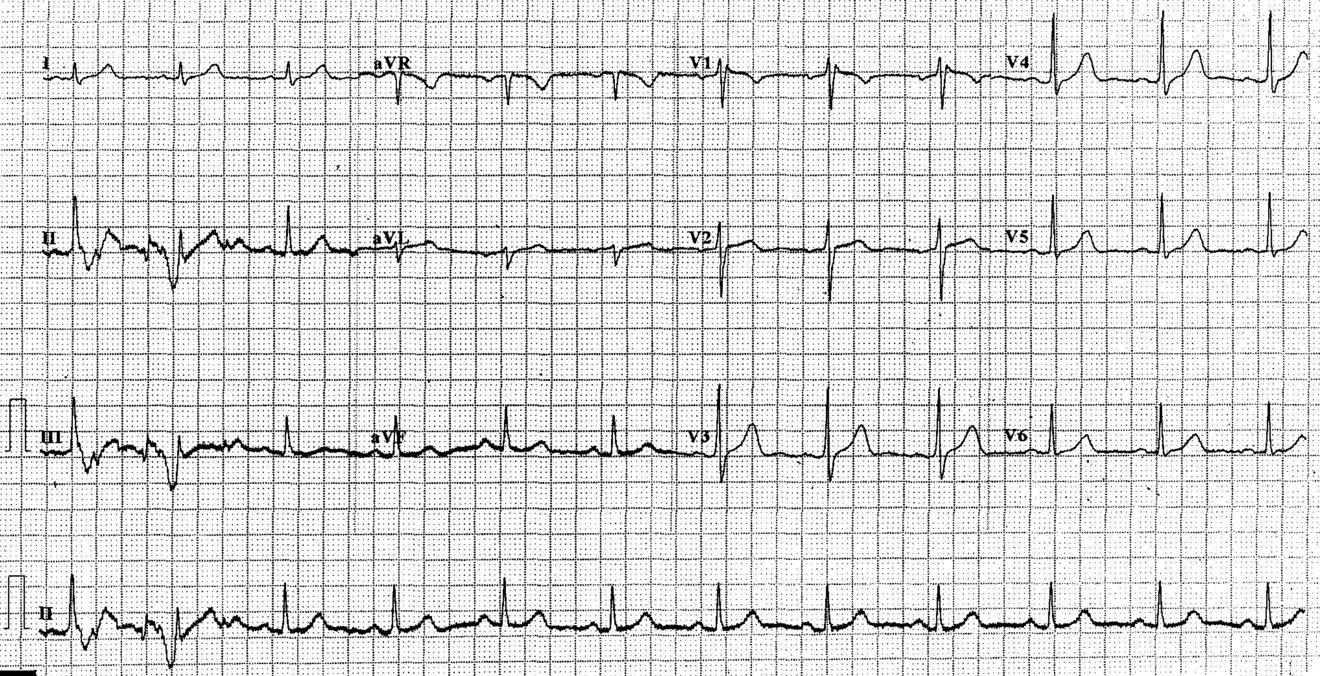

[redacted] 0.67-75Hz AC50 25mm/s 10mm/mV 4*2.5s+1r SE-1200Express V1.824 SEMIP V1.7

61872

[illegible] Jul-20-2015 7:19 AM [illegible] CE 0197

ID:
Name:
Sex: Birth Date: Years
cm kg mmHg
Medication:
Symptoms:
History:

1100 Sinus rhythm
9110 ** normal ECG **

Vent rate	70	bpm
PR int	154	ms
QRS dur	106	ms
QT/QTc int	388/ 408	ms
P/QRS/T axis	63/ 88/ 46	°
RV5/SV1 amp	0.935/ 0.515	mV
RV5+SV1 amp	1.450	mV

Unconfirmed Report
Reviewed by:

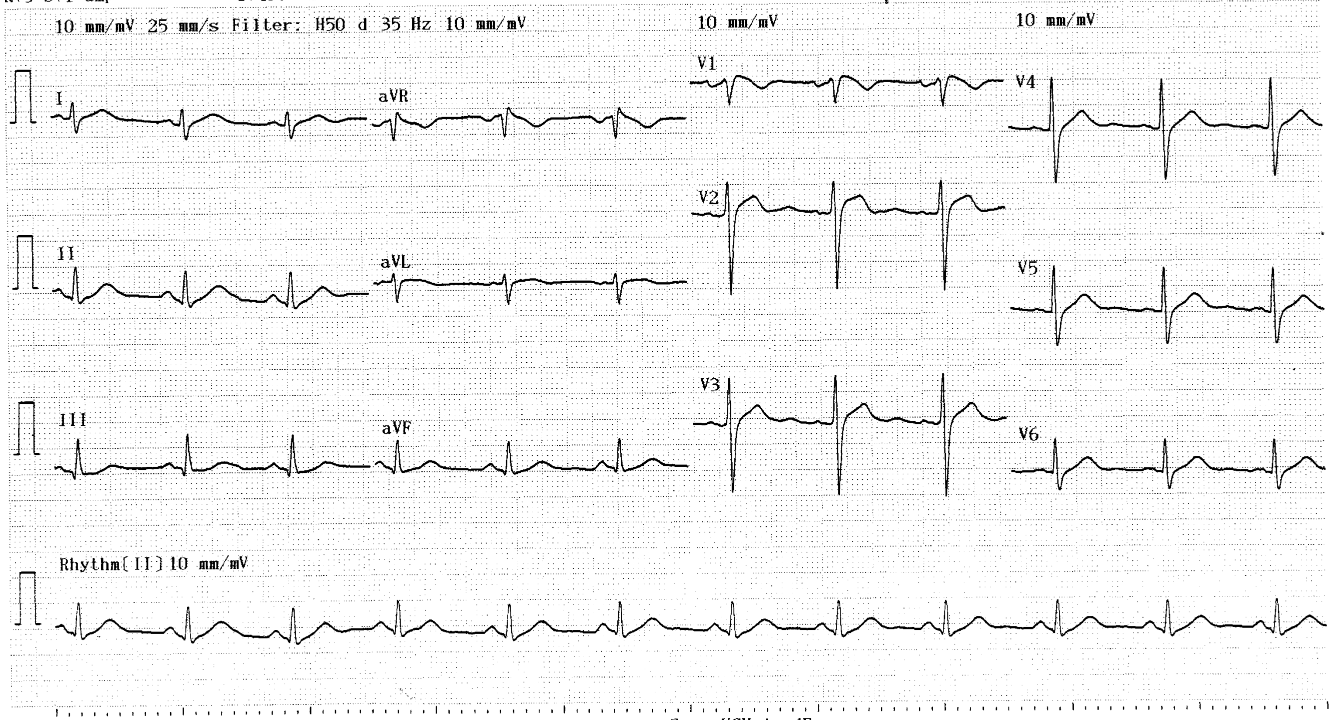

1350K 03-01 02-52 Dept.: Exam:UCH AandE

61872

HR	: 98	bpm
P	: 113	ms
PR	: 168	ms
QRS	: 112	ms
QT/QTc	: 349/447	ms
P/QRS/T	: 47/51/47	°
RV5/SV1	: 1.411/0.378	mV

Diagnosis Information:
Sinus Rhythm
Biphasic T Wave(V2)
Slight ST Elevation(V4)

Unconfirmed Report.

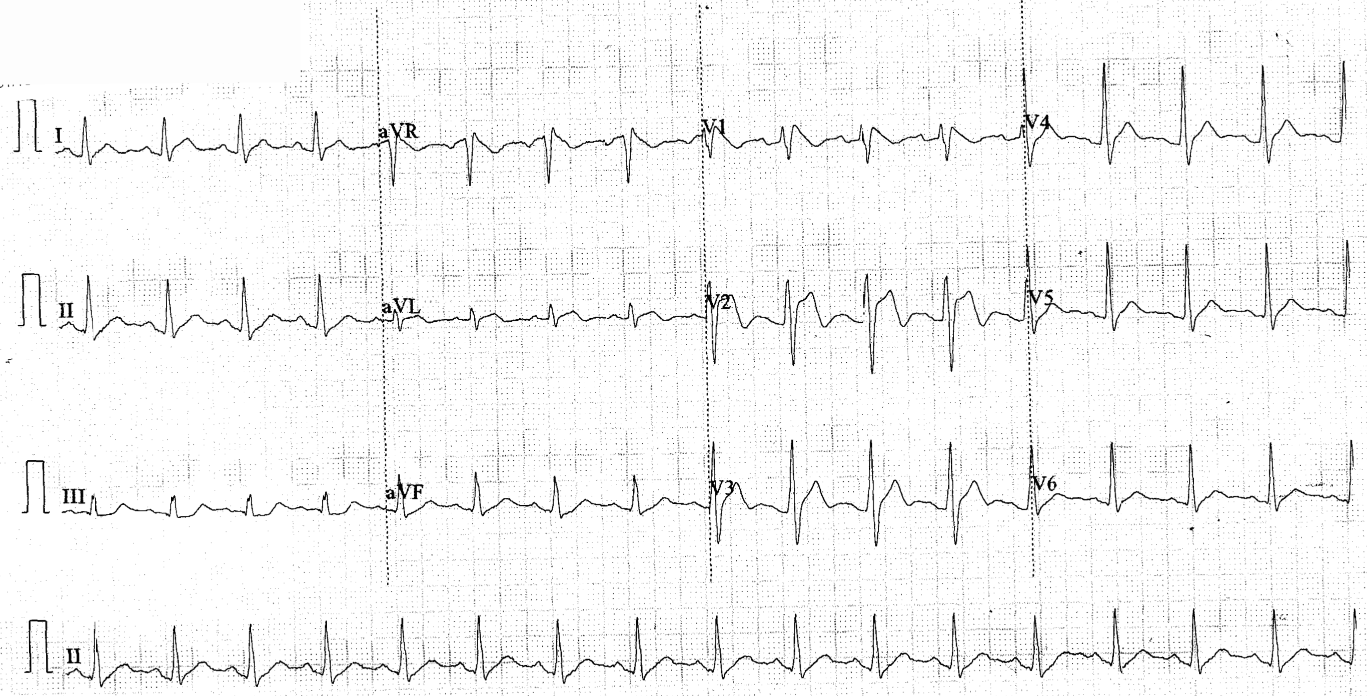

0.67~25Hz AC50 25mm/s 10mm/mV 4*2.5s+1r ♥98 SE-1200Express V2.01 SEMIP V1.7

30-Mar-2013 15:19:51

Rate	97	. AGE NOT ENTERED, ASSUMED TO BE 50 YEARS FOR PURPOSE OF ECG INTERPRETATION
PR	188	. NORMAL SINUS RHYTHM, RATE 97....................normal P axis, PR, rate & rhythm
QRSD	107	. BORDERLINE LEFT ATRIAL ABNORMALITY.............................P>30mS, <-.10mV V1
QT	341	. INCOMPLETE RIGHT BUNDLE BRANCH BLOCK...............QRS>100, terminal axis(90,270)
QTc	433	

--Axis--

P	62
QRS	69
T	20

- ABNORMAL ECG -

Unconfirmed diagnosis.

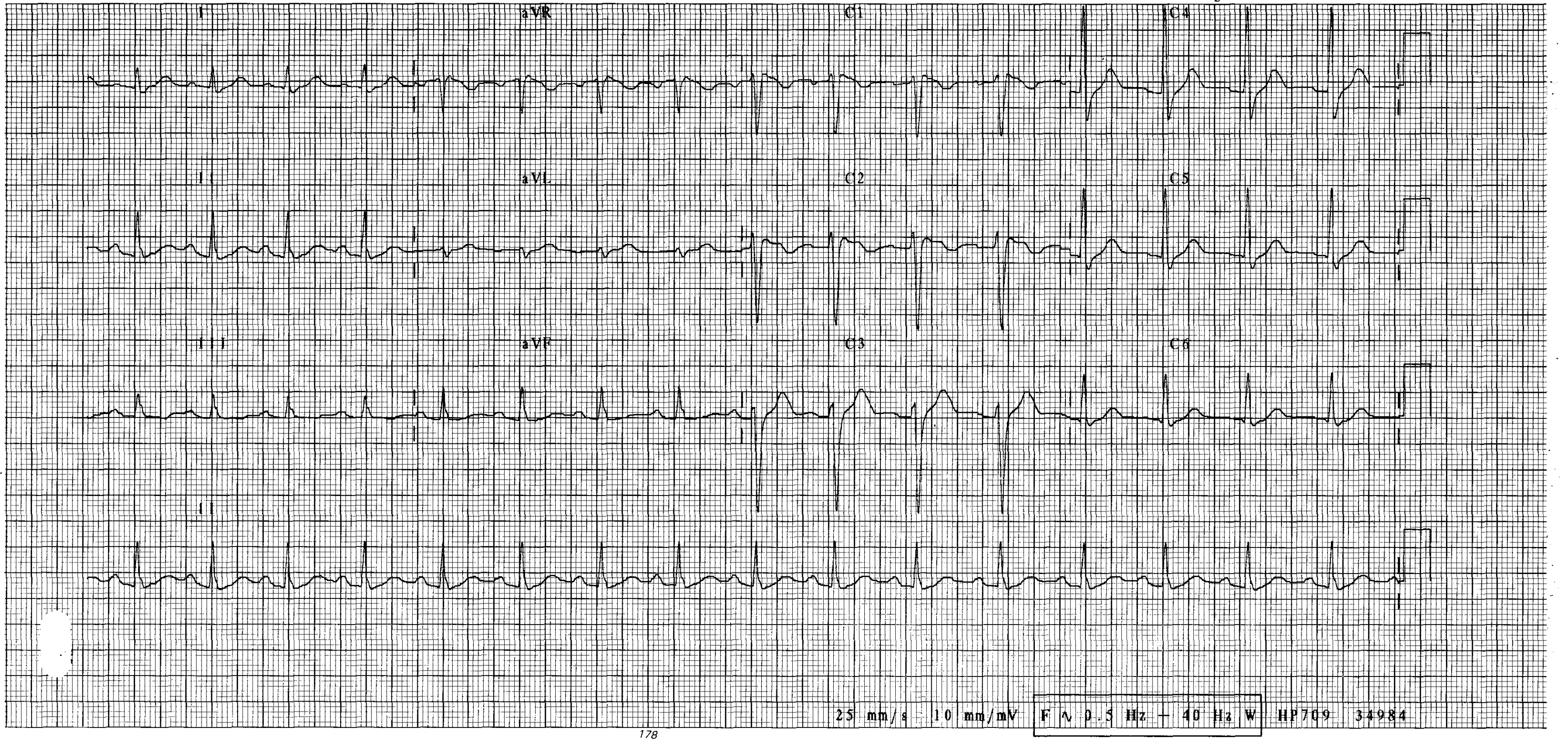

178

26222
