# Supplementary material for: Higher Dispersion Measures of Conduction and Repolarization in Type 1 Compared to Non-type 1 Brugada Syndrome Patients: An Electrocardiographic Study From a Single Center
Source: Front Cardiovasc Med. 2018 Oct 4;5:132. doi: 10.3389/fcvm.2018.00132 (PMC6180153; doi:10.3389/fcvm.2018.00132)
Supplement: Supplementary file 2 [file Data_Sheet_2.PDF]

h18597 09-Dec-2009 21:14:02 fug kam la

Rate	61	. AGE NOT ENTERED, ASSUMED TO BE 50 YEARS FOR PURPOSE OF ECG INTERPRETATION
PR	141	. NORMAL SINUS RHYTHM, RATE 61....................normal P axis, PR, rate & rhyth
QRSD	90	
QT	412	
QTc	415	

--Axis--

P	81
QRS	11
T	98

- NORMAL ECG - Unconfirmed diagnosis.

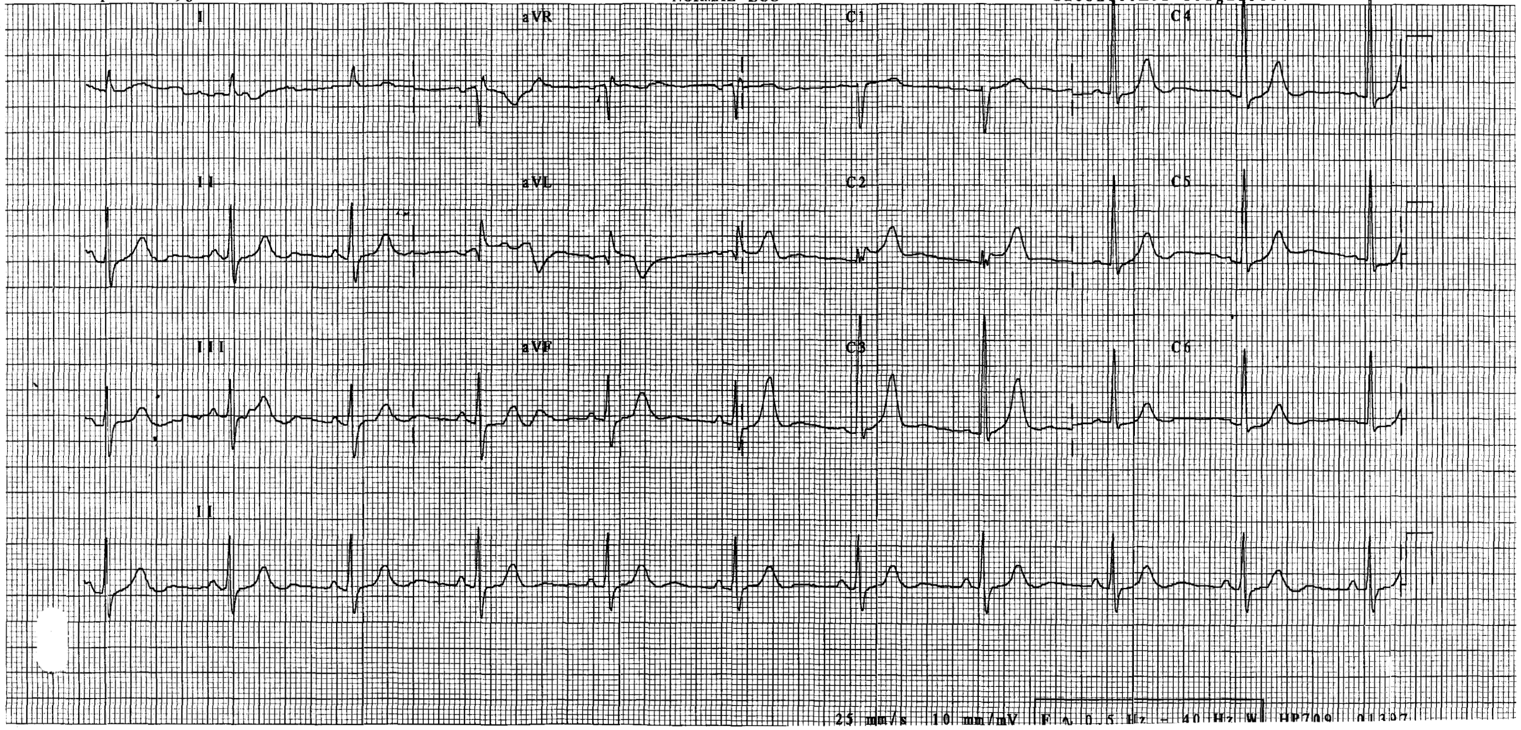

26222

2012/12/16 20:13:41

PWH A&E

Rate	82	. AGE IS NOT ENTERED, ASSUMED TO BE 50 YEARS OLD FOR PURPOSE OF ECG INTERPRETATION
PR	156	. SINUS RHYTHM.......................................normal P axis, V-rate 50- 99
QRSD	90	. EARLY PRECORDIAL R/S TRANSITION.........................QRS area positive in V2
QT	364	. ST ELEV, PROBABLE NORMAL EARLY REPOL PATTERN................ST elevation, age<55
QTc	425	

--AXIS--

P	77
QRS	64
T	52

- OTHERWISE NORMAL ECG -

Unconfirmed Diagnosis

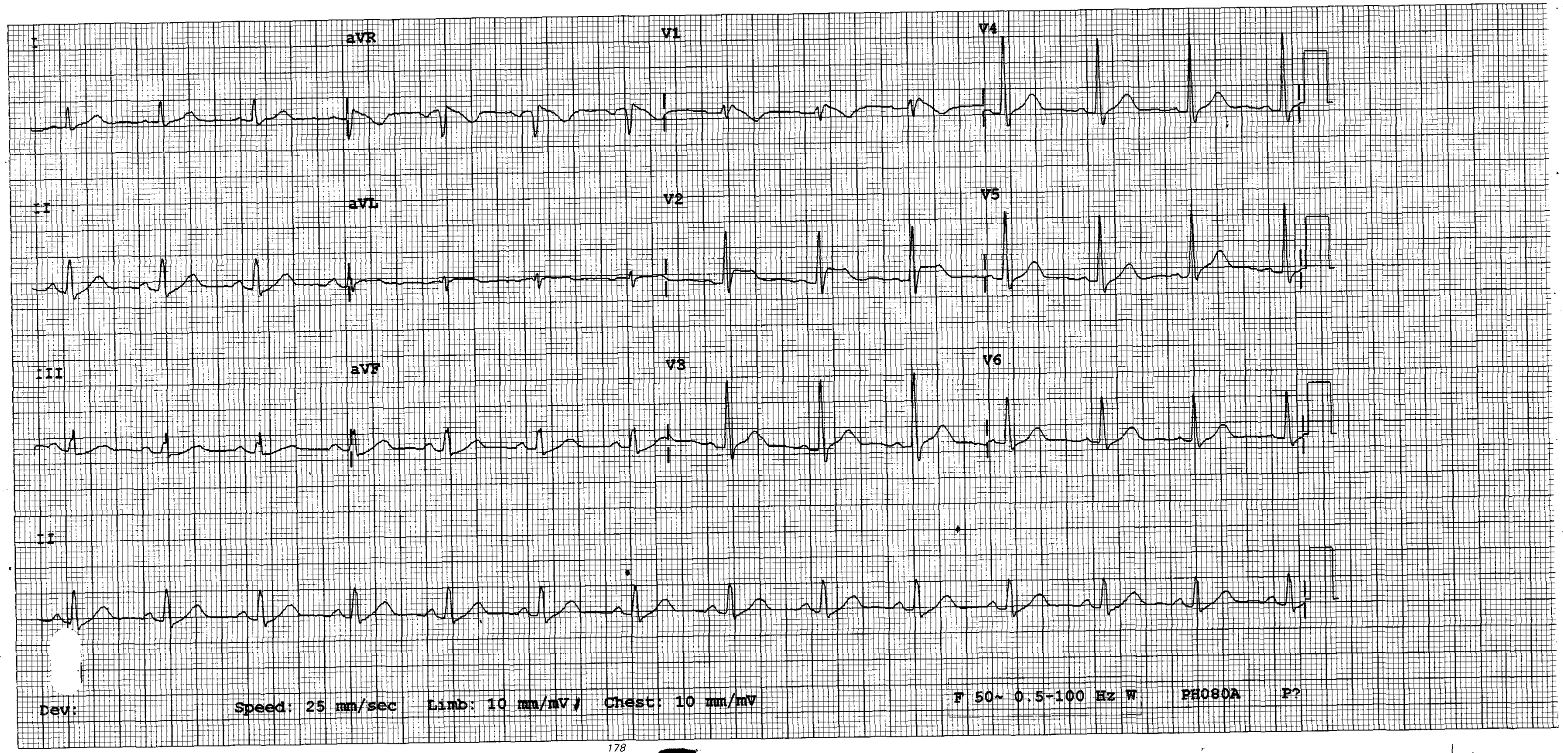

178

26222

2/16/2012 5:17:49 PM

Rate 47	. AGE IS NOT ENTERED, ASSUMED TO BE 50 YEARS OLD FOR PURPOSE OF ECG INTERPRETATION
PR 163	. SINUS BRADYCARDIA...V-rate< 50
QRSD 100	. LEFT VENTRICULAR HYPERTROPHY......................(SV1+RV5)>3.5/(RaVL+SV3)>2.80
QT 484	. ANTEROLATERAL INFARCT, RECENT..................Q >35mS, ST >0.07mV, T neg, V2-V6
QTc 428	

--AXIS--
P 80
QRS 65
T 73

(2)

- ABNORMAL ECG -

Unconfirmed Diagnosis

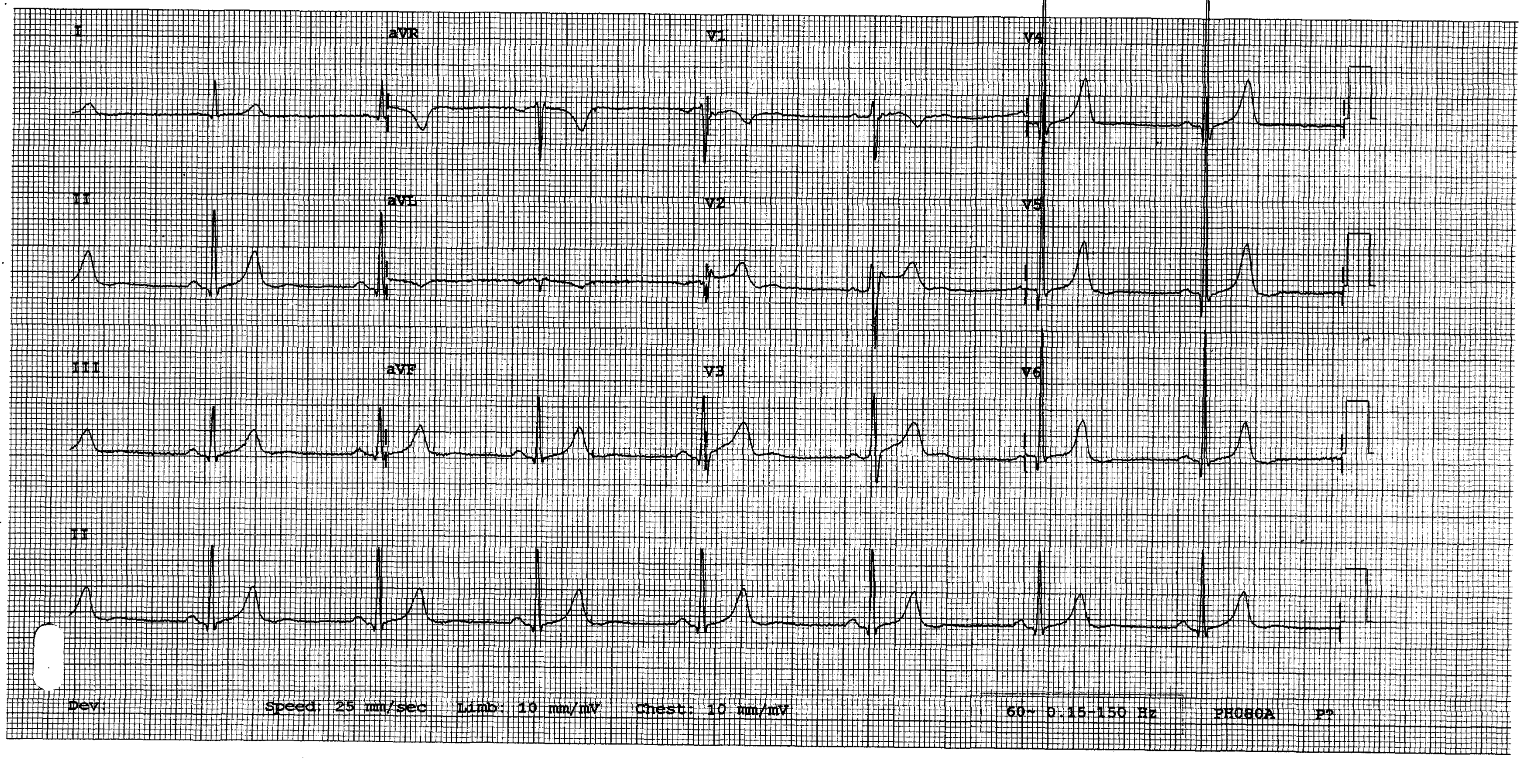

28222

06-06-2015 18:43:49 For Use On EDAN 01.57.32462 CE 0217565

ID :
Name :
Age : Years
Medication :

HR	: 116	BPM
P Dur	: 125	ms
PR int	: 197	ms
QRS Dur	: 101	ms
QT/QTC int	: 341/475	ms
P/QRS/T axis	: 39/82/13	°
RV5/SV1 amp	: 1.631/0.455	mV
RV5+SV1 amp	: 2.086	mV
RV6/SV2 amp	: 1.052/1.031	mV

Diagnosis Information:
812: Sinus Tachycardia
734: Suspect Anteroseptal Myocardial Infarction? (V1,V2)
621: Inverted T Wave(V5,V6)
661: Slight ST Depression(V4,V5,V6)

Unconfirmed Report.

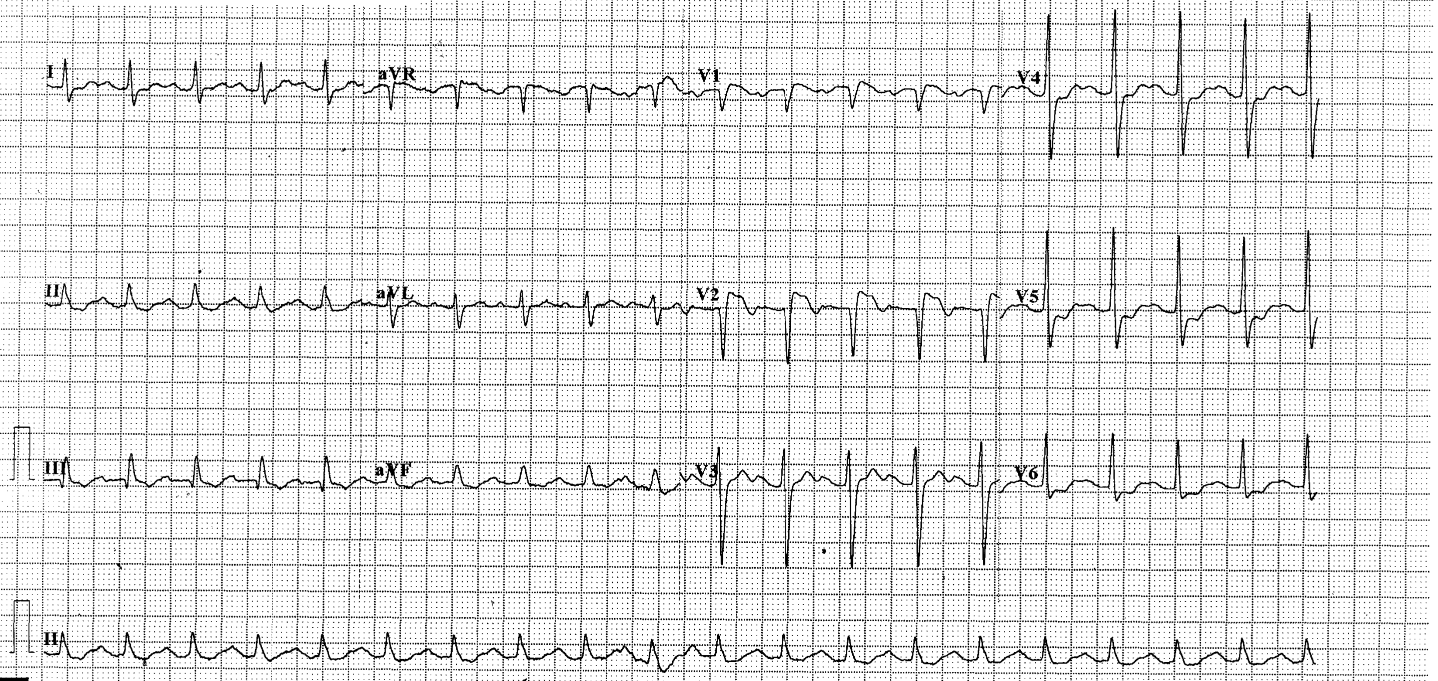

0.67~25Hz AC50 25mm/s 10mm/mV 4*2.5s+1r SE-1200Express V1.823 SEMIP V1.7

06-09-2017 11:53:32 032
ID:

Years

HR	: 70	bpm
P	: 109	ms
PR	: 142	ms
QRS	: 95	ms
QT/QTc	: 401/435	ms
P/QRS/T	: 90/81/69	°
RV5/SV1	: 2.721/0.450	mV

Diagnosis Information:
Sinus Rhythm
Middle ST Elevation(V2)

Unconfirmed Report.

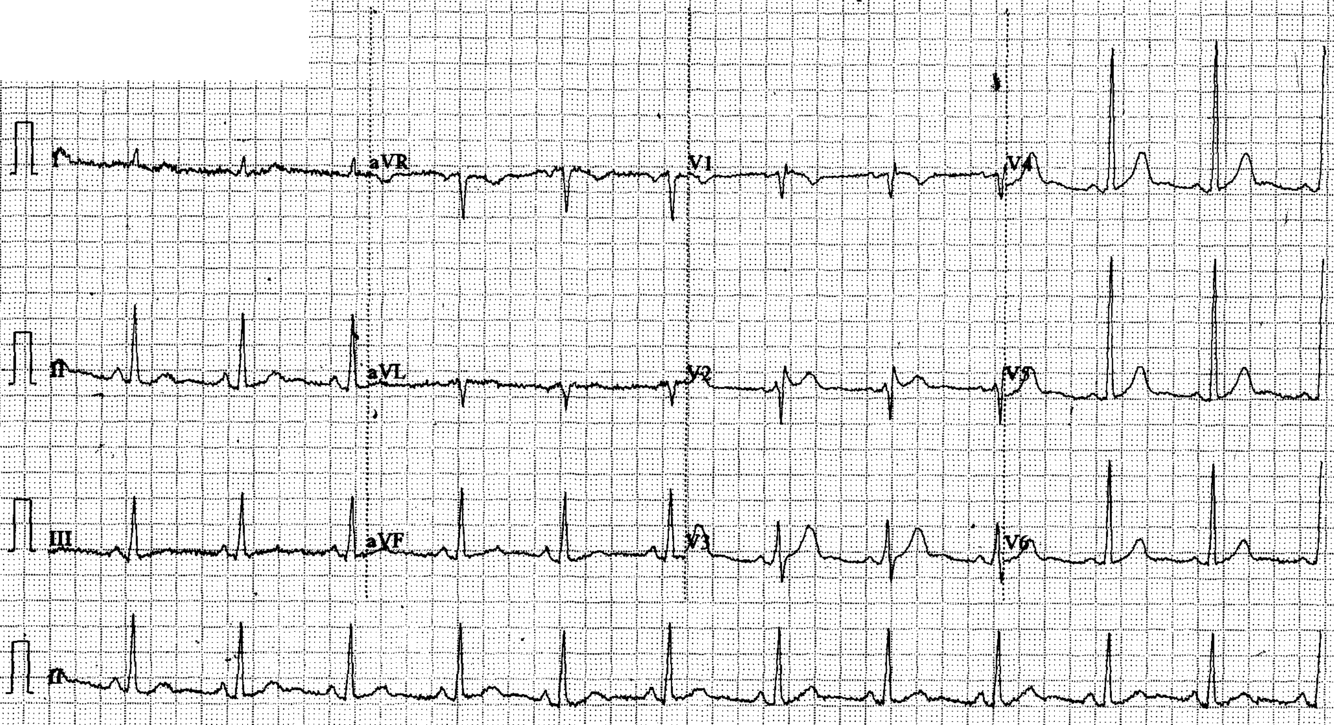

0.67-45Hz AC50 25mm/s 10mm/mV 4*2.5s+1r ♥70 SE-1200Express V2.01 SEMIP V1.7

26222

24-07-2016 18:14:42

0069299

ID: 564

HR	: 97	bpm
P	: 106	ms
PR	: 161	ms
QRS	: 84	ms
QT/QTc	: 368/470	ms
P/QRS/T	: 84/−18/80	°
RV5/SV1	: 0.604/0.581	mV

Diagnosis Information:

Sinus Rhythm

Poor R Wave Progression(V4)

Runs of Premature Ventricular Contraction

Low Voltage(Limb Leads)

Report Confirmed by:

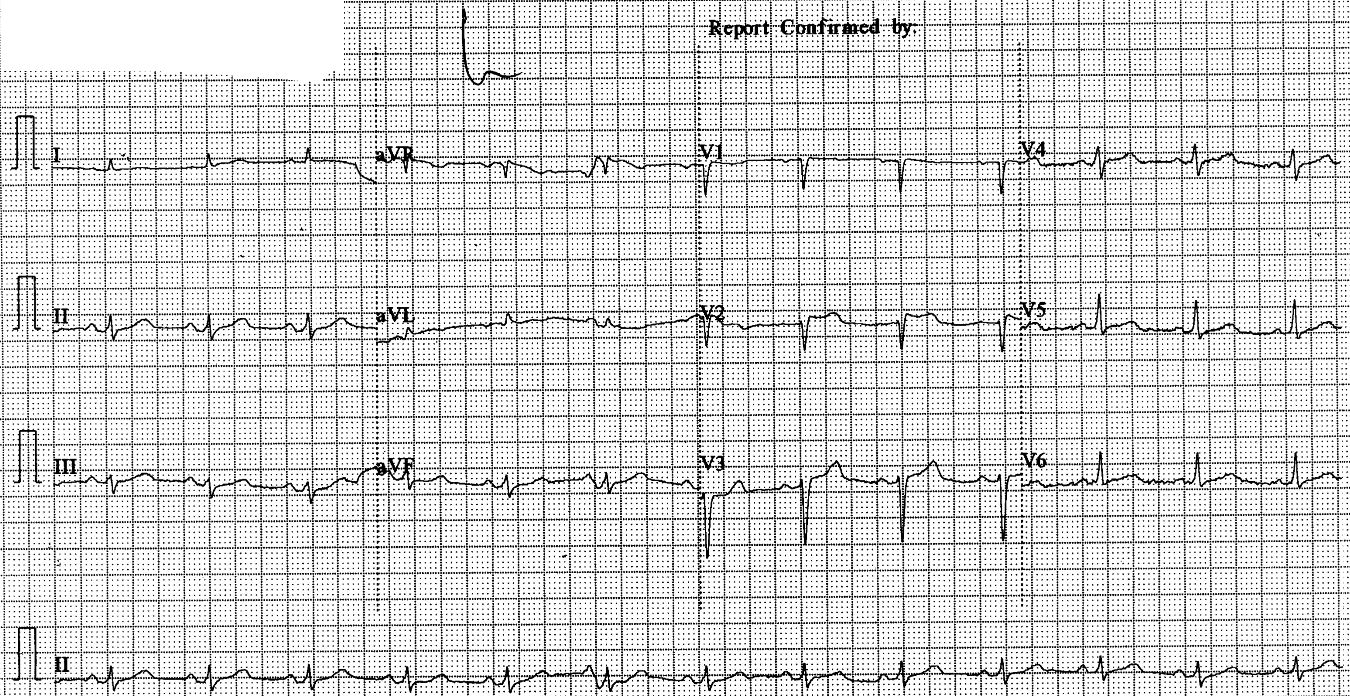

0.25~25Hz AC50 25mm/s 10mm/mV 4*2.5s+1r ♥97 SE-1200Express V2.01 SEMIP V1.7

2014-09-06 14:43:38 EDAN 01.57.32462

0105717

ID :
Name :
Age : Years

HR	:	60	BPM
P Dur	:	122	ms
PR int	:	160	ms
QRS Dur	:	95	ms
QT/QTC int	:	423/425	ms
P/QRS/T axis	:	83/68/75	°
RV5/SV1 amp	:	1.942/0.666	mV
RV5+SV1 amp	:	2.608	mV
RV6/SV2 amp	:	1.315/0.469	mV

Unconfirmed Report.

Diagnosis Information:
800: Sinus Rhythm
672: Middle ST Elevation(V2)

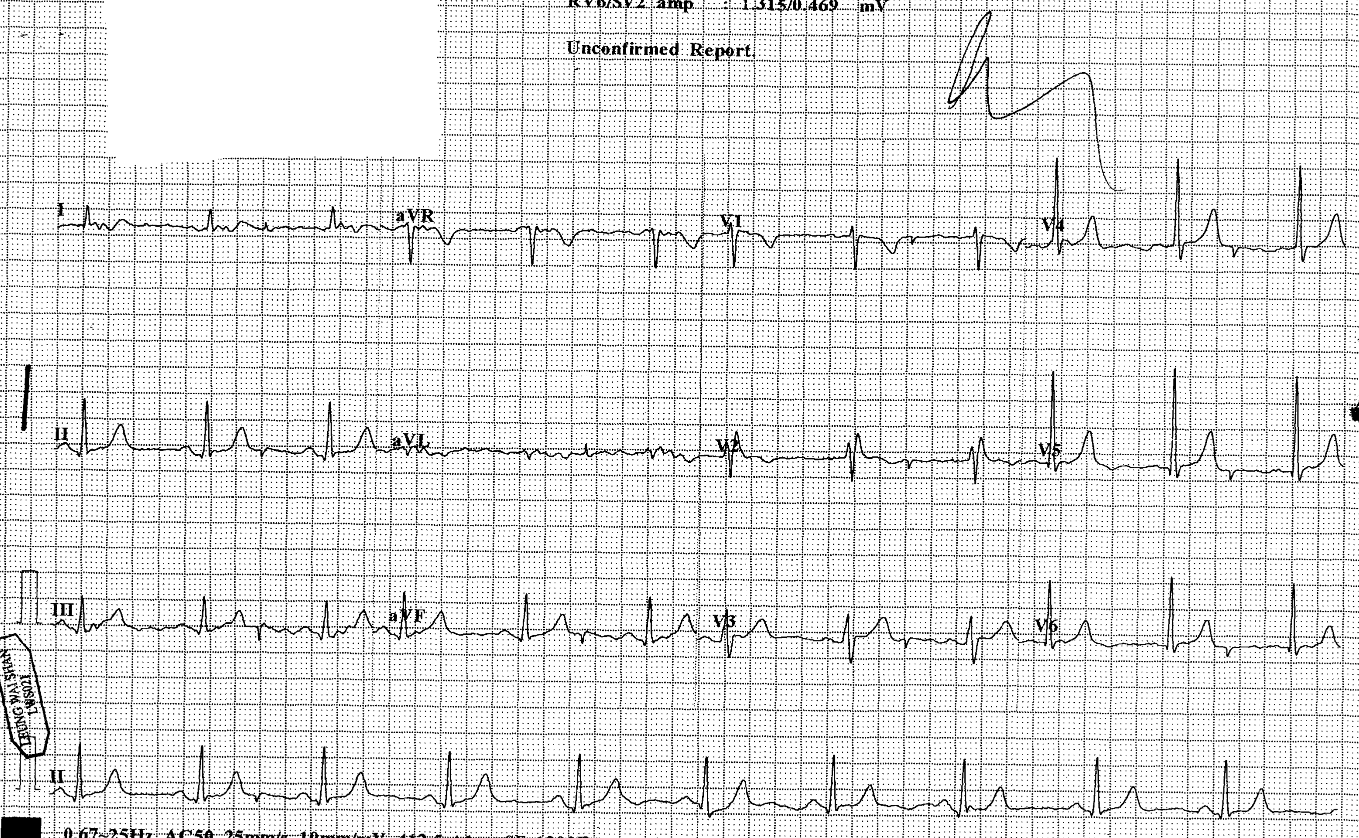

0.67~25Hz AC50 25mm/s 10mm/mV 4*2.5s+1r SE-1200Express V1.823 SEMIP V1.7

35003

20-01-2017 03:21:36
ID:
Years

HR	: 87	bpm
P	: 115	ms
PR	: 164	ms
QRS	: 97	ms
QT/QTc	: 338/408	ms
P/QRS/T	: 66/34/35	°
RV5/SV1	: 1.101/0.068	mV

Diagnosis Information:
Sinus Rhythm
Middle ST Elevation(V2)

known Brugada-type
Tx CCT

Unconfirmed Report.

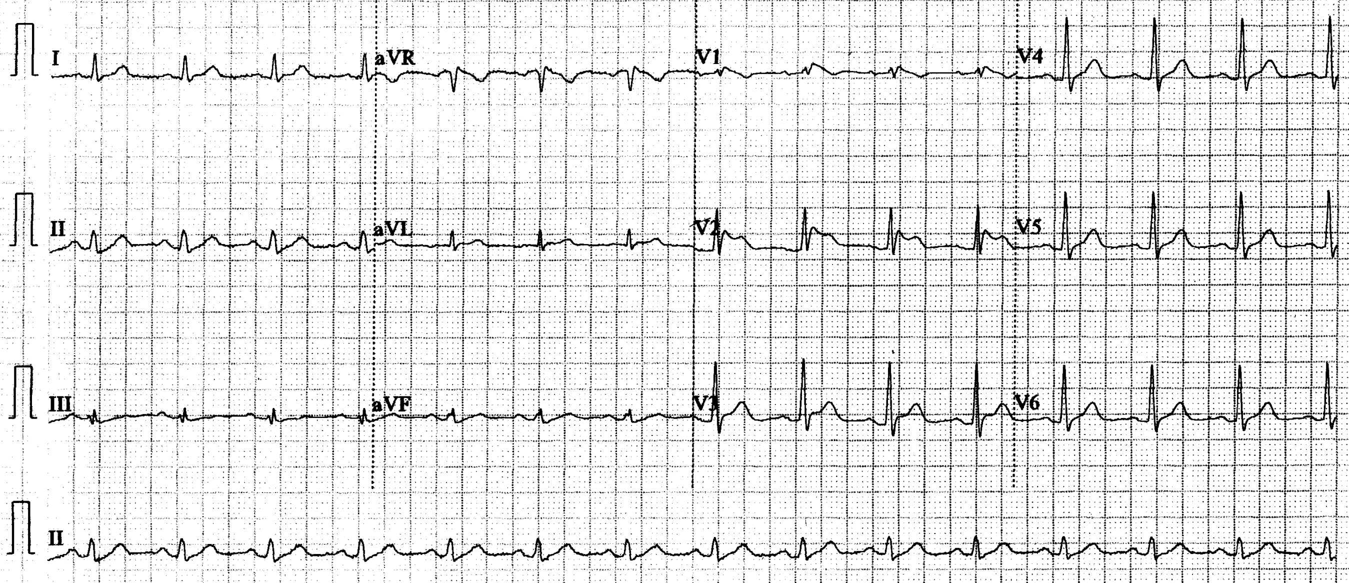

0.67~25Hz AC50 25mm/s 10mm/mV 4*2.5s+1r ♥87 SE-1200Express V2.01 SEMIP V1.7

15-Jun-2011 13:13:56

Rate	93	. AGE NOT ENTERED, ASSUMED TO BE 50 YEARS FOR PURPOSE OF ECG INTERPRETATION
PR	170	. NORMAL SINUS RHYTHM, RATE 93....................normal P axis, PR, rate & rhythm
QRSD	97	. BORDERLINE RIGHT AXIS DEVIATION..............................age-specific ranges
QT	333	. PROBABLE LEFT VENTRICULAR HYPERTROPHY.................LVH voltage with LAA or LAD
QTc	414	

--Axis--

P	72
QRS	82
T	50

- ABNORMAL ECG -

Unconfirmed diagnosis.

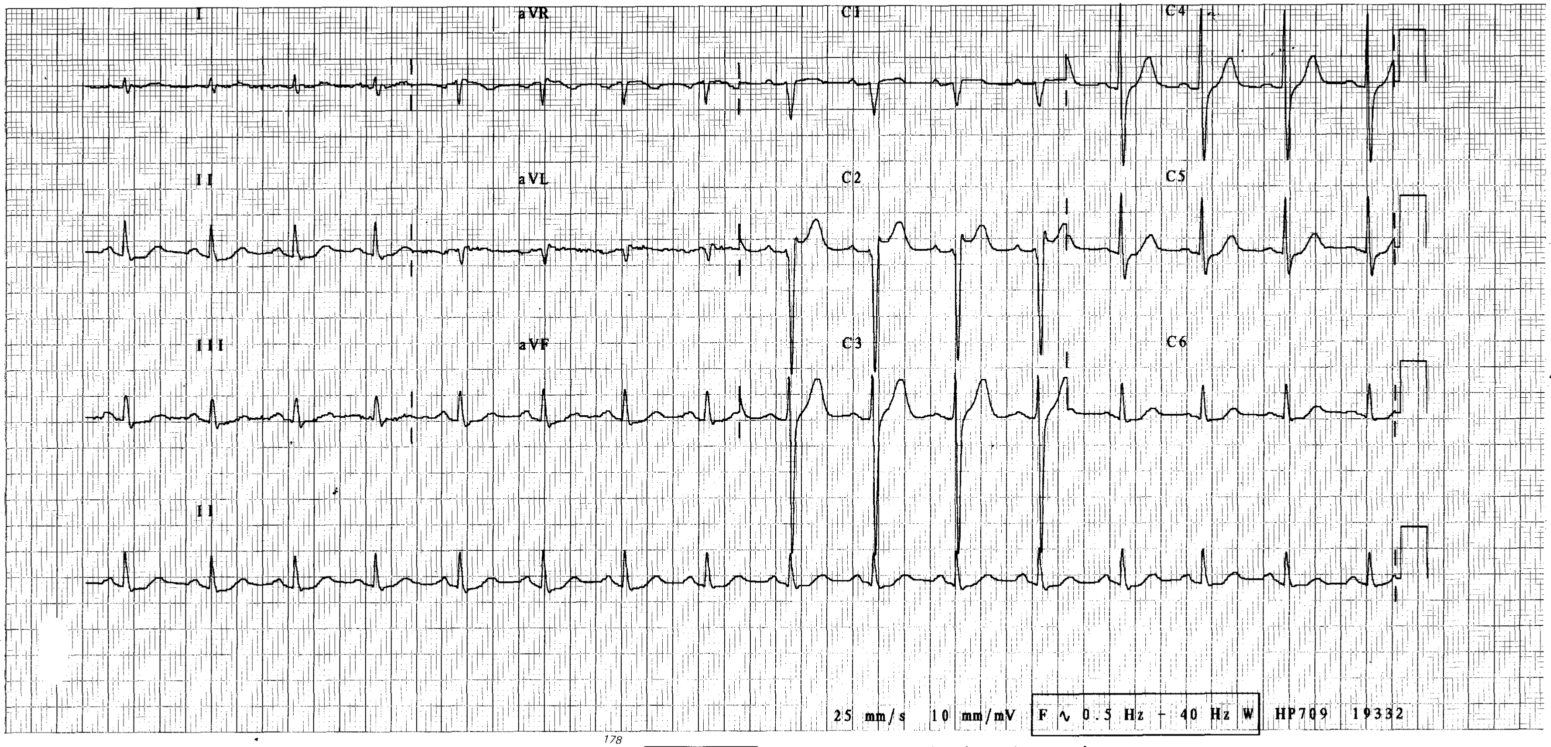

178

26222

24-May-2012 16:48:03

Rate	59	. AGE NOT ENTERED, ASSUMED TO BE 50 YEARS FOR PURPOSE OF ECG INTERPRETATION
PR	165	. NORMAL SINUS RHYTHM, RATE 59....................normal P axis, PR, rate & rhythm
QRSD	92	. EARLY TRANSITION WITH RSR' IN V1 OR V2..............QRS area positive & R' V1/V2
QT	387	
QTc	383	

--Axis--

P	60
QRS	45
T	44

- OTHERWISE NORMAL ECG - Unconfirmed diagnosis.

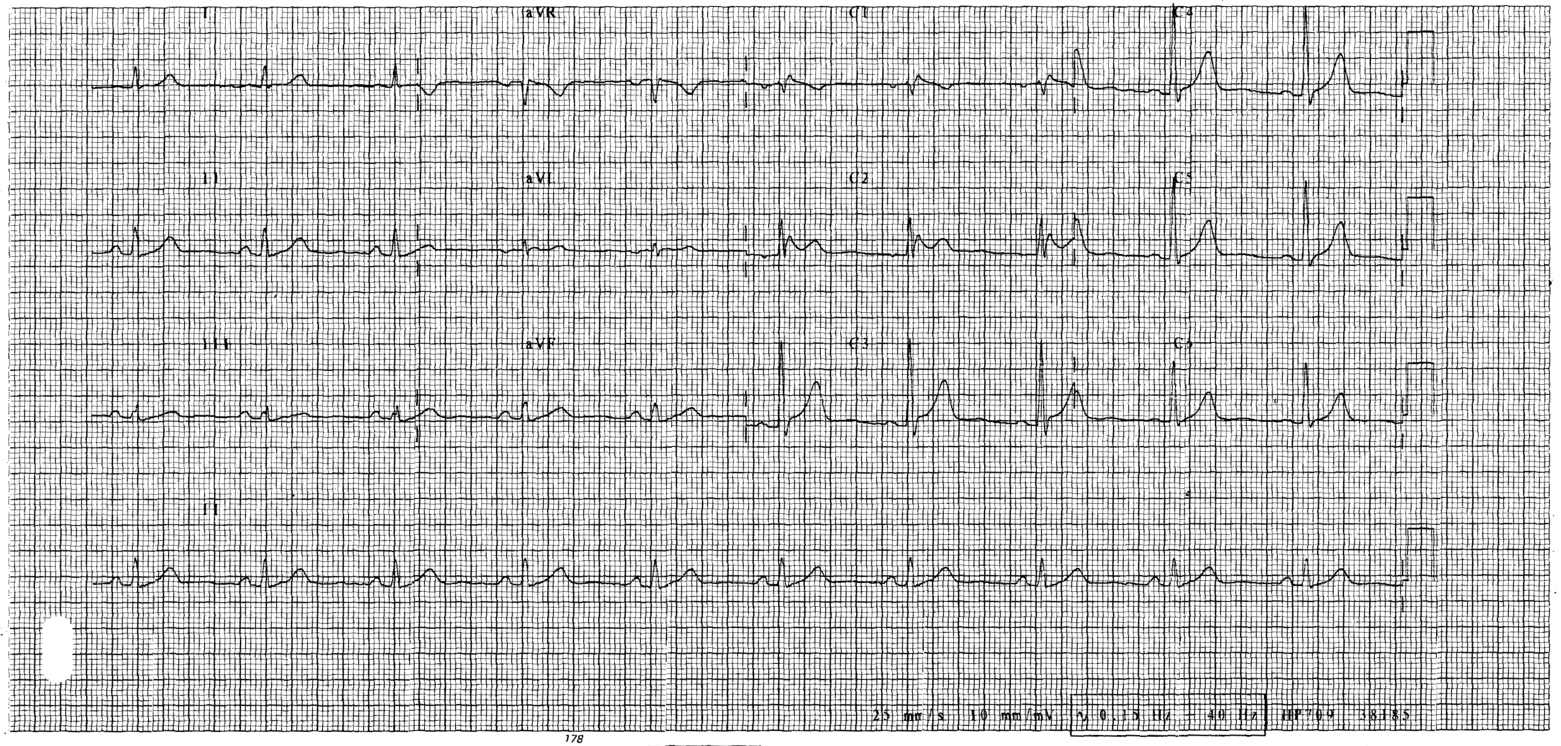

178

26222

24-May-2012 16:48:03

Rate	59	. AGE NOT ENTERED, ASSUMED TO BE 50 YEARS FOR PURPOSE OF ECG INTERPRETATION
PR	165	. NORMAL SINUS RHYTHM, RATE 59.....................normal P axis, PR, rate & rhythm
QRSD	92	. EARLY TRANSITION WITH RSR' IN V1 OR V2...............QRS area positive & R' V1/V2
QT	387	
QTc	383	

--Axis--

P	60
QRS	45
T	44

- OTHERWISE NORMAL ECG -

Unconfirmed diagnosis.

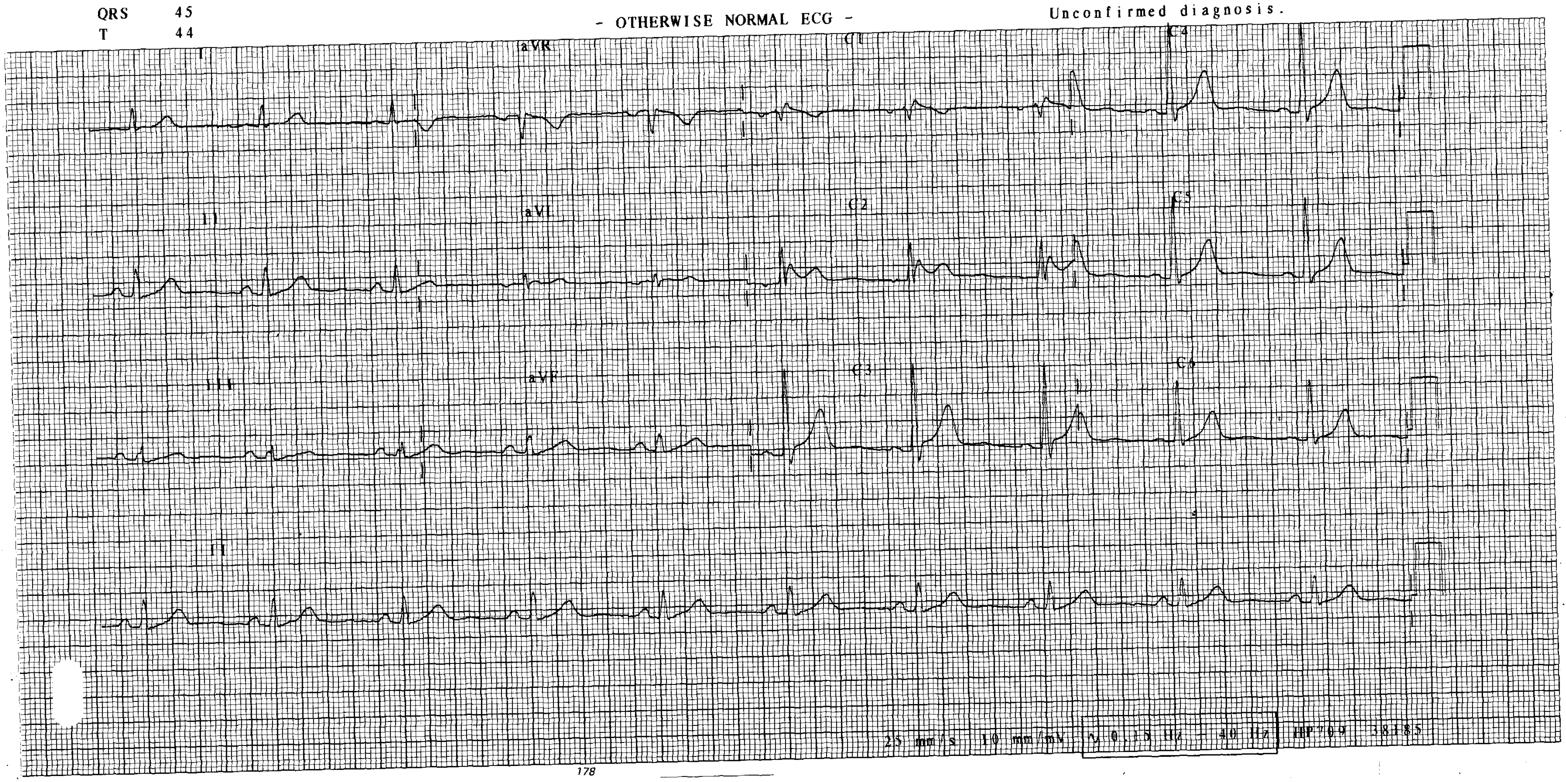

26222

22-02-2016 10:12:58
ID :
Name :
Age : Years

HR	: 72	BPM
P Dur	: 121	ms
PR int	: 174	ms
QRS Dur	: 86	ms
QT/QTC int	: 388/425	ms
P/QRS/T axis	: 52/22/31	°
RV5/SV1 amp	: 1.165/0.098	mV
RV5+SV1 amp	: 1.263	mV
RV6/SV2 amp	: 0.949/0.111	mV

Unconfirmed Report.

Diagnosis Information:
800: Sinus Rhythm
Normal ECG

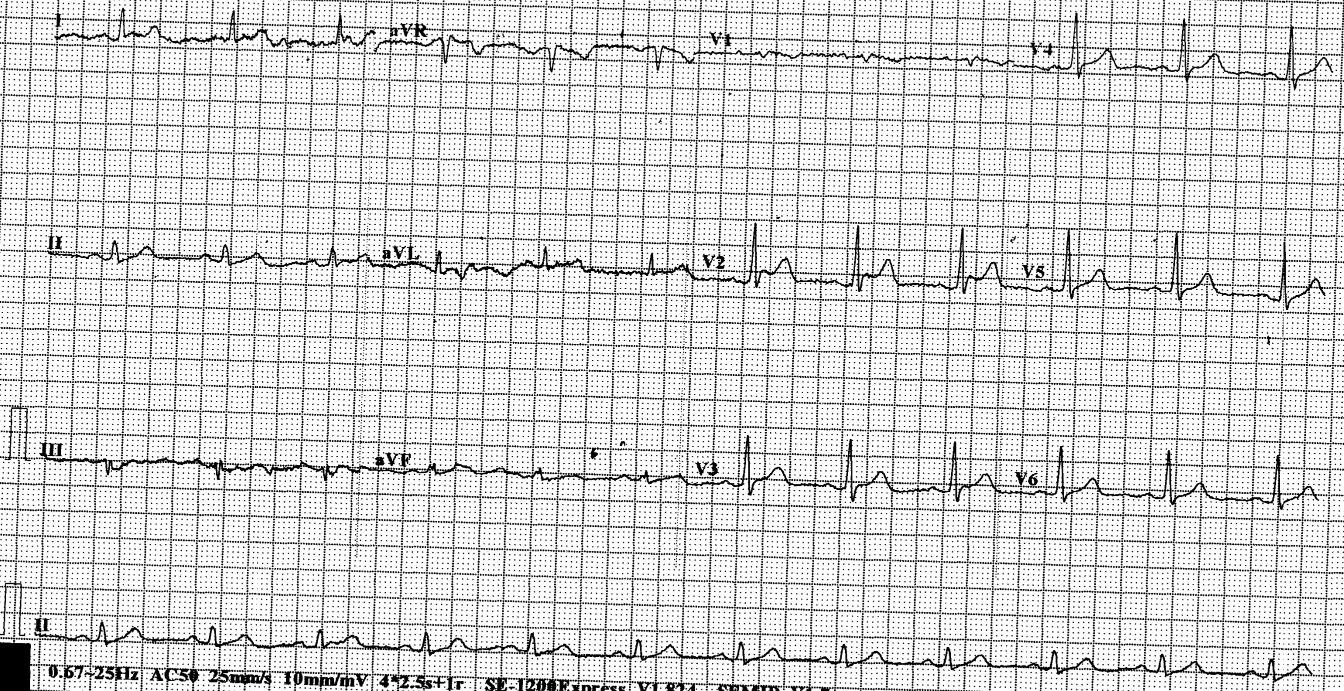

0.67~25Hz AC50 25mm/s 10mm/mV 4*2.5s+1r SE-1200Express V1.824 SEMIP V1.7

25-09-2016 20:51:34 For Use On EDAN 01.57.32462 CE

ID:

0023263

Years

HR	: 78	bpm
P	: 126	ms
PR	: 184	ms
QRS	: 91	ms
QT/QTc	: 363/415	ms
P/QRS/T	: 58/41/67	°
RV5/SV1	: 0.720/0.406	mV

Diagnosis Information:

Sinus Rhythm

Normal ECG

Unconfirmed Report.

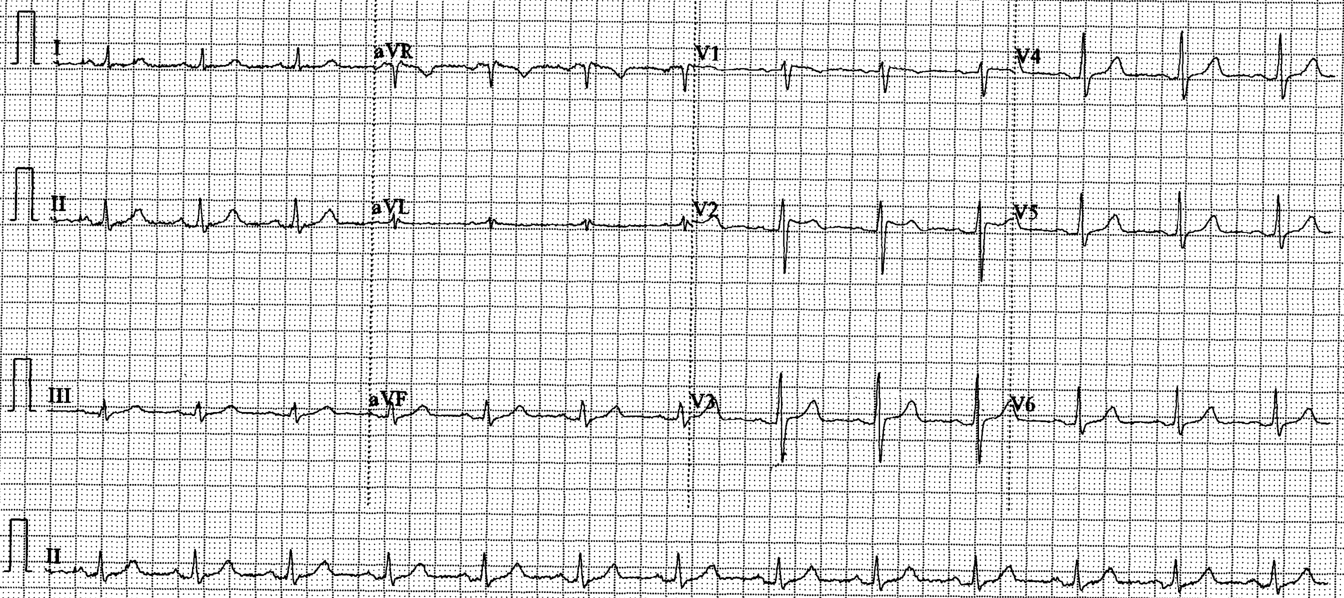

0.67~45Hz AC50 25mm/s 10mm/mV 4*2.5s+1r ♥78 SE-1200Express V2.01 SEMIP V1.7

28222

13-06-2018 14:41:17

ID
Nam
Age

HR	:	66	BPM
P Dur	:	120	ms
PR int	:	177	ms
QRS Dur	:	98	ms
QT/QTC int	:	404/423	ms
P/QRS/T axis	:	77/-28/60	°
RV5/SV1 amp	:	1.846/0.311	mV
RV5+SV1 amp	:	2.157	mV
RV6/SV2 amp	:	1.589/0.000	mV

Diagnosis Information:
800: Sinus Rhythm
671: Slight ST Elevation(V2)

Unconfirmed Report.

(2)

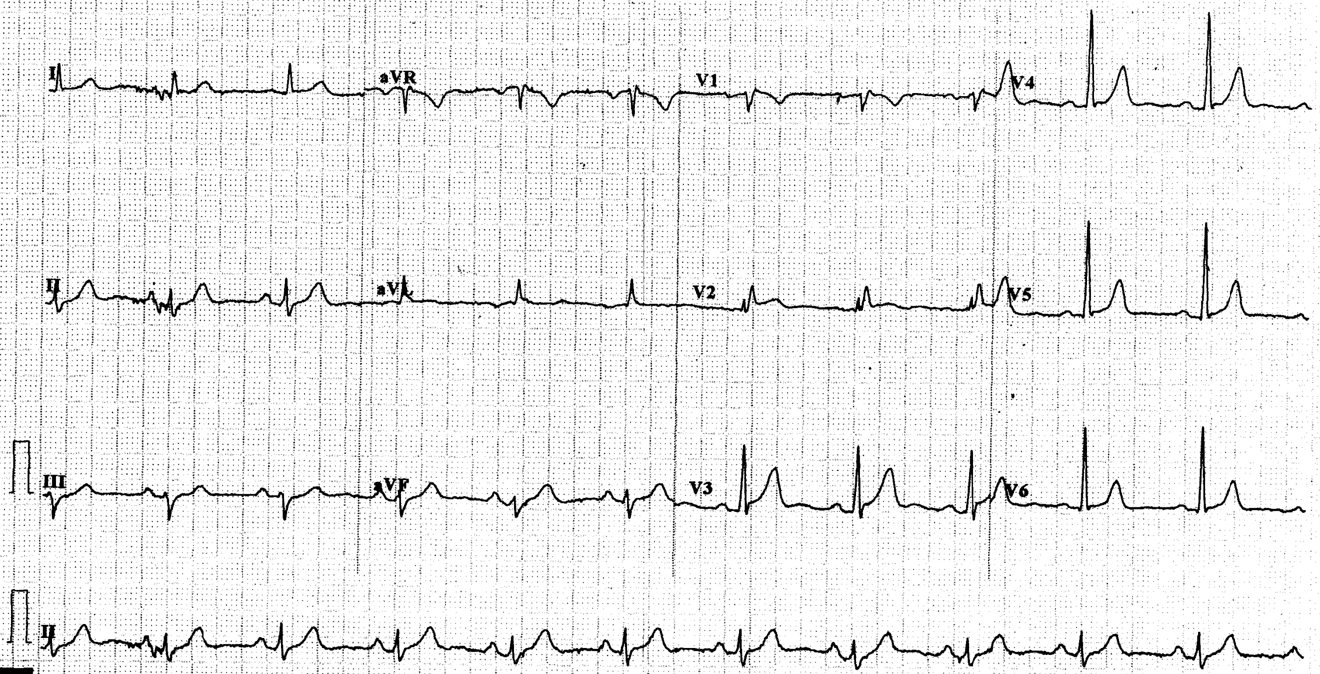

0.67~25Hz AC50 25mm/s 10mm/mV 4*2.5s+1r SE-1200Express V1.824 SEMIP V1.7

61872

ID:
Name:
Sex: Birth Date: Years
cm kg mmHg
Medication:
Symptoms:
History:

Feb- 5-2010 2:41 PM

1100 Sinus rhythm
2320 Nonspecific intraventricular conduction delay
9130 ** borderline ECG **

Vent rate	65	bpm
PR int	160	ms
QRS dur	116	ms
QT/QTc int	412/ 424	ms
P/QRS/T axis	50/ 38/ 41	°
RV5/SV1 amp	1.885/ 0.600	mV
RV5+SV1 amp	2.485	mV

Unconfirmed Report
Reviewed by:

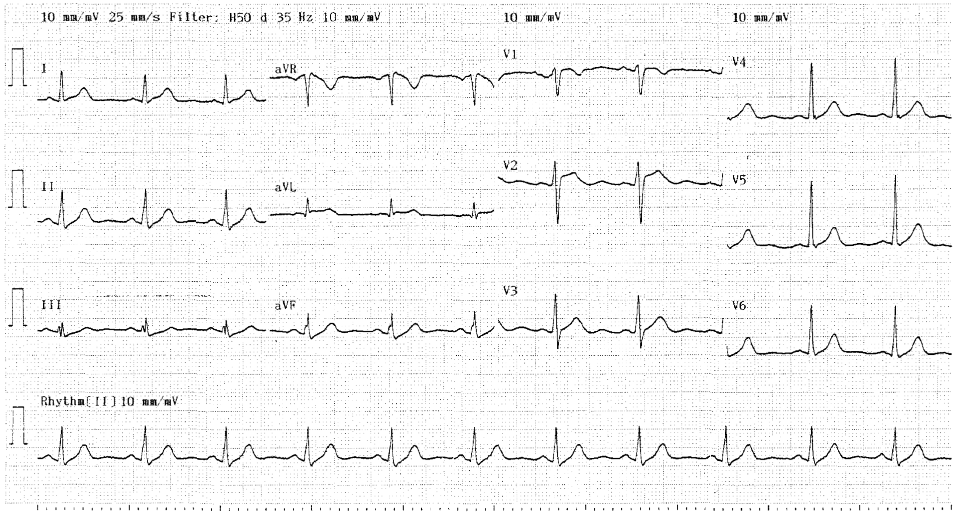

1350K 03-01 02-52 Dept.:
Exam:UCH AED

ID:
Name:
Sex: Birth Date: Years
cm kg mmHg
Medication:
Symptoms:
History:

Feb- 5-2010 1:42 PM

1100 Sinus rhythm
2320 Nonspecific intraventricular conduction delay
4436 Possible septal injury or acute infarct
9150 ** abnormal ECG **

Vent rate	70	bpm
PR int	158	ms
QRS dur	120	ms
QT/QTc int	410/ 431	ms
P/QRS/T axis	61/ 51/ 52	°
RV5/SV1 amp	2.285/ 0.805	mV
RV5+SV1 amp	3.090	mV

Unconfirmed Report
Reviewed by:

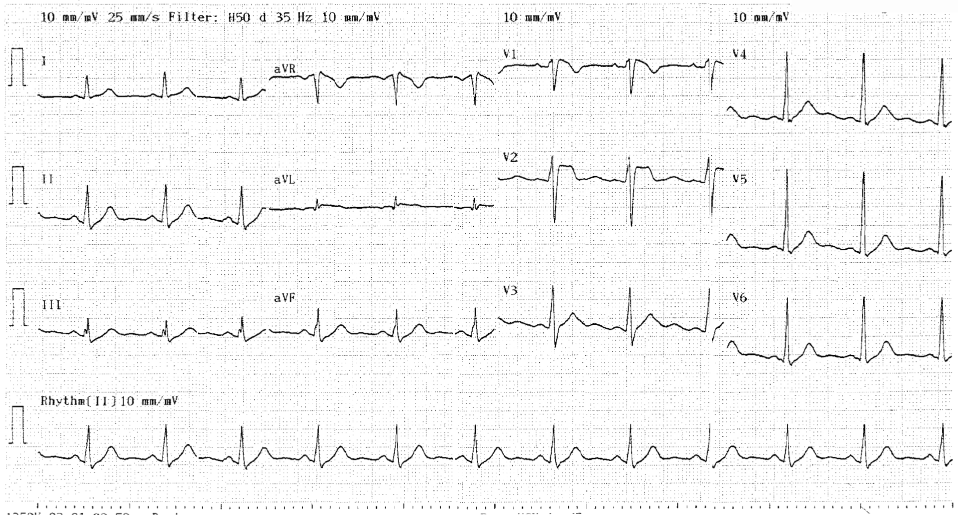

61872

25005

10-Sep-2010 23:43:04

Rate	123	. AGE NOT ENTERED, ASSUMED TO BE 50 YEARS FOR PURPOSE OF ECG INTERPRETATION
PR	147	. SINUS TACHYCARDIA, RATE 123..............................normal P axis, rate>=100
QRSD	89	
QT	28[illegible]	
QTc	410	

--Axis--

P	64
QRS	64
T	48

- OTHERWISE NORMAL ECG -

Unconfirmed diagnosis.

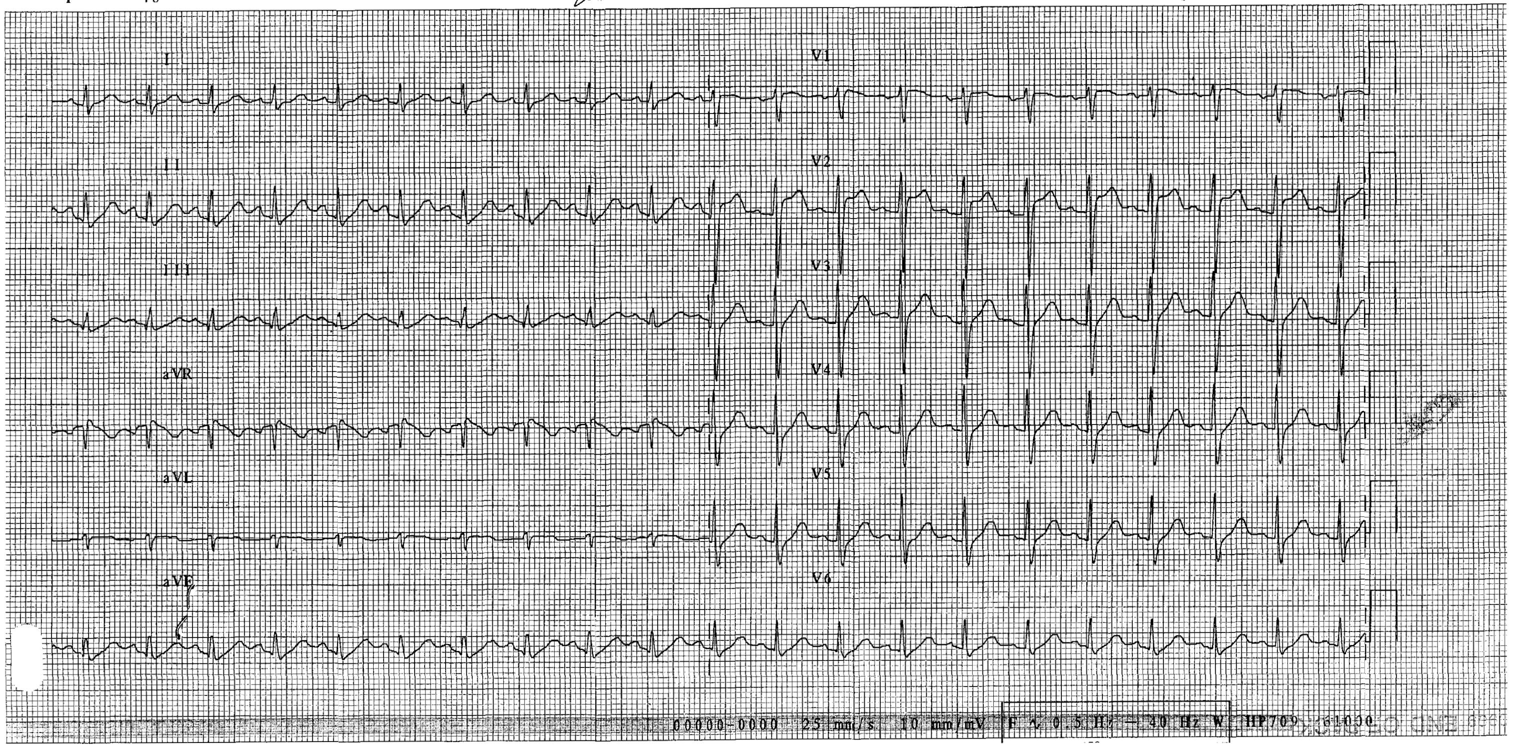

00000-0000 25 mm/s 10 mm/mV F ~ 0.5 H[illegible] – 40 Hz W HP709 [illegible]1000

04-06-2017 10:20:05
ID:

Years

HR	: 67	bpm
P	: 119	ms
PR	: 171	ms
QRS	: 95	ms
QT/QTc	: 387/409	ms
P/QRS/T	: 70/45/46	°
RV5/SV1	: 0.738/0.109	mV

Diagnosis Information:

Sinus Rhythm
Slight ST Elevation(V2)
Low Voltage(Limb Leads)

Unconfirmed Report.

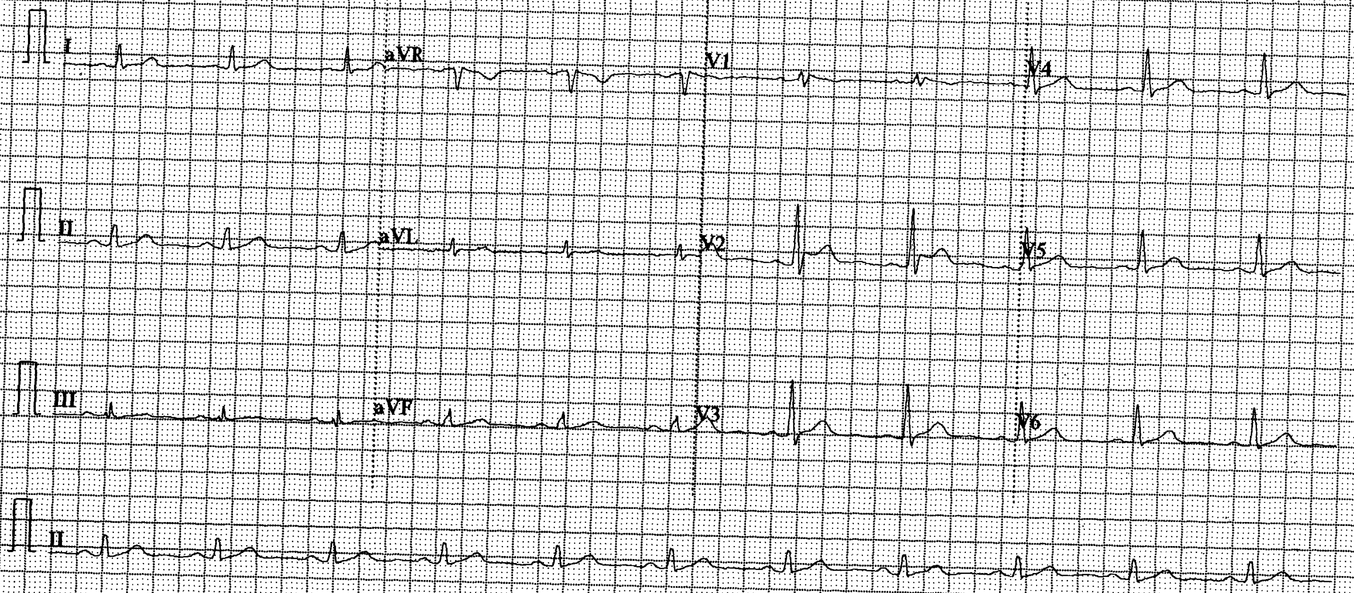

0.67-25Hz AC50 25mm/s 10mm/mV 4*2.5s+1r ♥67 SE-1200Express V2.01 SEMIP V1.7

21-01-2014 17:03:0[illegible]

ID :

Name :

Age : Years

Medication :

HR	: 155	BPM
P Dur	: 116	ms
PR int	: 150	ms
QRS Dur	: 99	ms
QT/QTC int	: 304/488	ms
P/QRS/T axis	: 145/37/42	
RV5/SV1 amp	: 0.883/0.493	mV
RV5+SV1 amp	: 1.376	mV
RV6/SV2 amp	: 0.736/1.572	mV

Unconfirmed Report.

Diagnosis Information:

812: Sinus Tachycardia

701: Poor R Wave Progression(V3)

631: Biphasic T Wave(V2,V6)

672: Middle ST Elevation(V2)

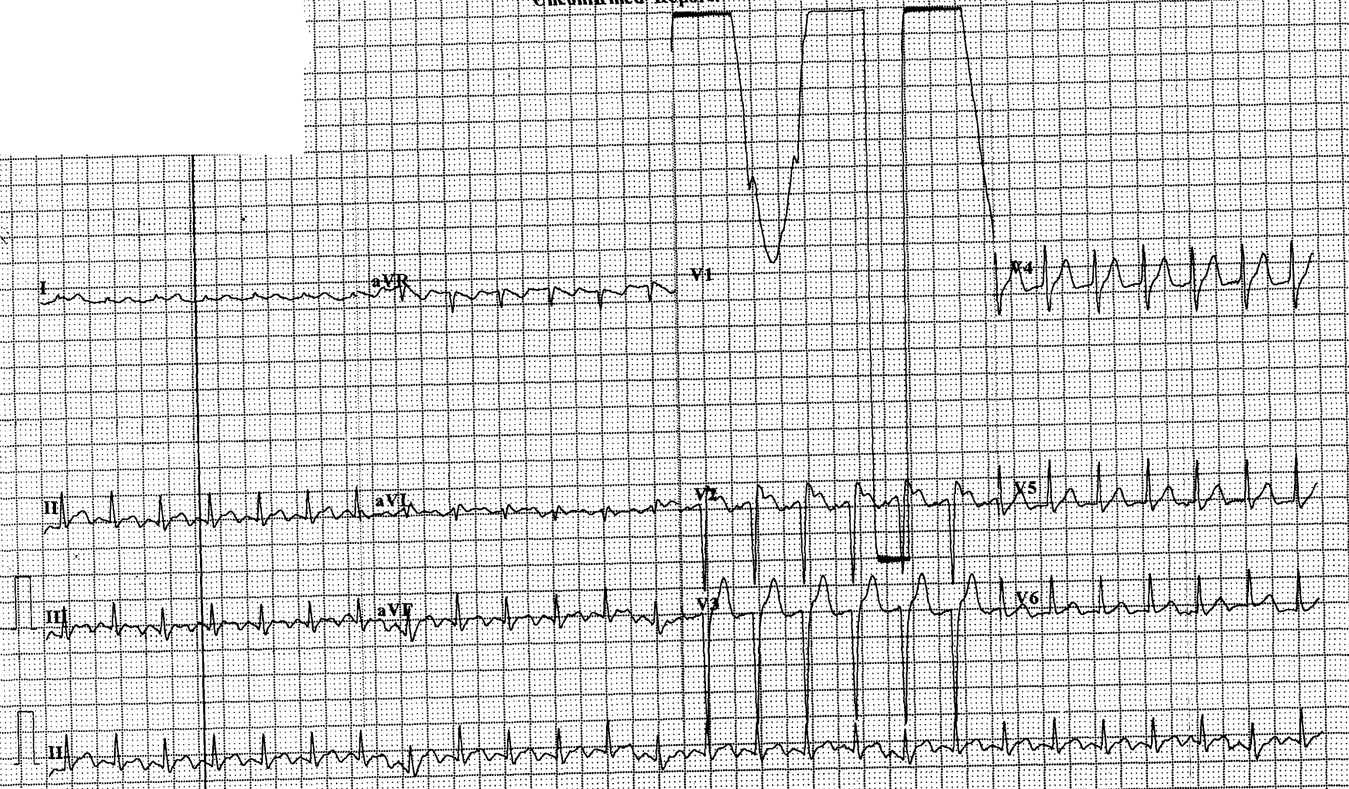

0.67~25Hz AC50 25mm/s 10mm/mV 4*2.5s+1r SE-1200Express V1.823 SEMIP V1.7

14-07-2015 11:29:56 AM

ID:

0053831

Years

HR	: 86	bpm
P	: 122	ms
PR	: 164	ms
QRS	: 108	ms
QT/QTc	: 352/423	ms
P/QRS/T	: 74/82/70	°
RV5/SV1	: 1.574/0.050	mV

Diagnosis Information:
Sinus Rhythm
Normal ECG

Unconfirmed Report.

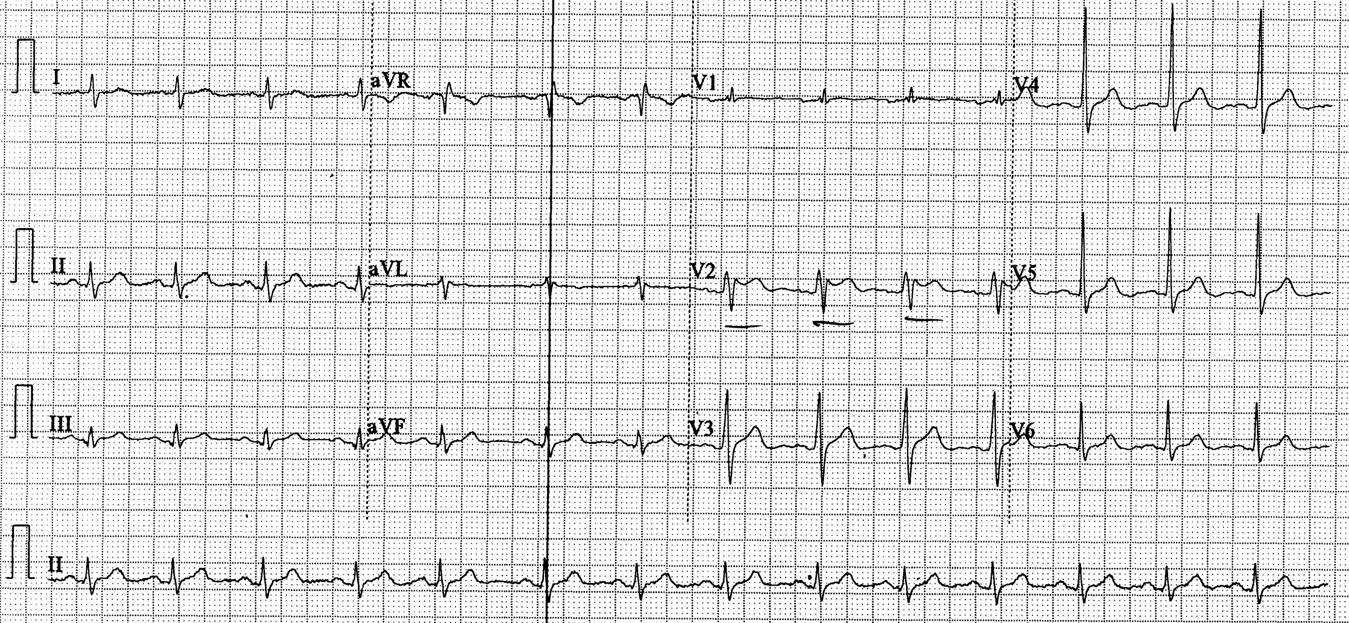

[redacted] 0.67~25Hz AC50 25mm/s 10mm/mV 4*2.5s+1r ♥86 SE-1200Express V2.01 SEMIP V1.7

24-Oct-2011 03:42:26

Rate	52	. AGE NOT ENTERED, ASSUMED TO BE 50 YEARS FOR PURPOSE OF ECG INTERPRETATION
PR	0	. ACCELERATED JUNCTIONAL ESCAPE RH'M, RATE 52...........absent P waves, rate 40-70
QRSD	89	. ATRIAL PREMATURE COMPLEX..........................short R-R interval, normal QRSD
QT	439	. RSR' IN V1 OR V2...small R' only
QTc	408	

--Axis--
P
QRS 64
T 51

(2)

- ABNORMAL ECG - Unconfirmed diagnosis.

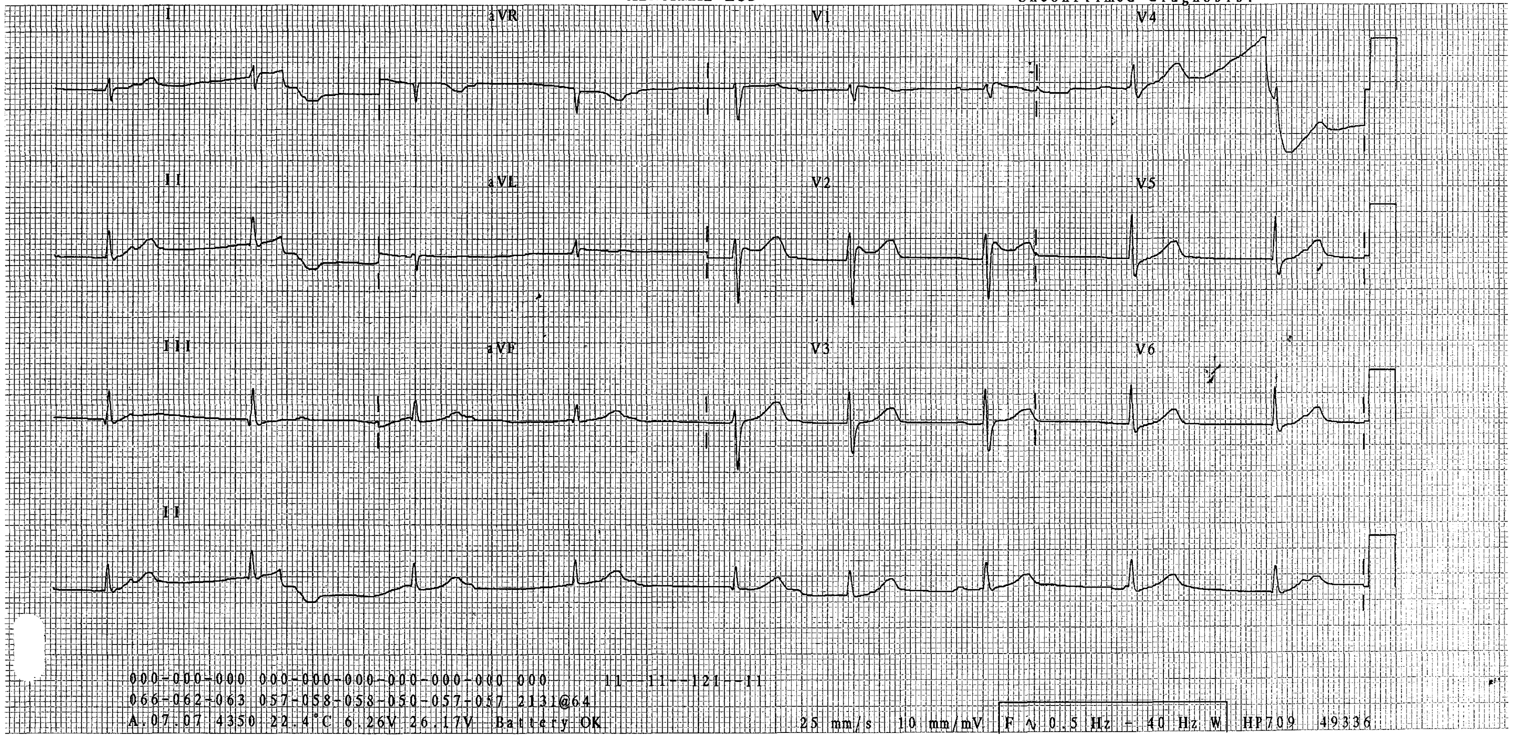

000-000-000 000-000-000-000-000-000 000 11--11--121--11
066-062-063 057-058-058-050-057-057 2131@64
A.07.07 4350 22.4°C 6.26V 26.17V Battery OK 25 mm/s 10 mm/mV F ∿ 0.5 Hz - 40 Hz W HP709 49336

12011

02-12-2017 10:05:27
ID:

HR	: 68	bpm
P	: 117	ms
PR	: 169	ms
QRS	: 97	ms
QT/QTc	: 380/406	ms
P/QRS/T	: 77/33/34	°
RV5/SV1	: 1.459/0.113	mV

Diagnosis Information:
Sinus Rhythm
Low Voltage(Limb Leads)

Unconfirmed Report.

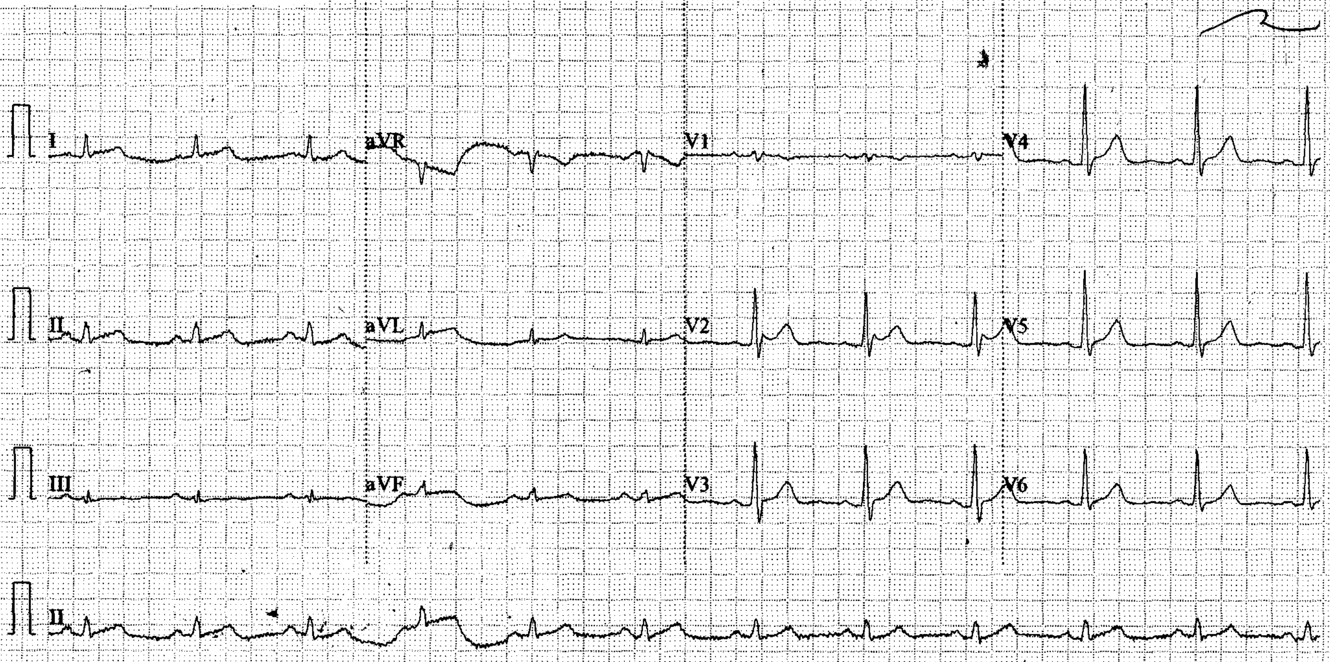

0.67~45Hz AC50 25mm/s 10mm/mV 4*2.5s+1r ♥68 SE-1200Express V2.01 SEMIP V1.7

26222

ID: **#STAT#140224165231** 02/24/2014 16:52:33

*** CONSIDER ACUTE STEMI ***
Sinus rhythm.
Right bundle branch block
Anterolateral ST elevation, CONSIDER ACUTE INFARCT

Abnormal ECG

* Unconfirmed Analysis *

D.O.B.:	
Meds:	
Class:	
Dr:	
Tech:	

Vent. Rate:	74 bpm
RR Interval:	808 ms
PR Interval:	178 ms
QRS Duration:	158 ms
QT Interval:	380 ms
QTc Interval:	404 ms
QT Dispersion:	48 ms
P Axis:	54 deg
QRS Axis:	56 deg
T Axis:	34 deg

Comment:

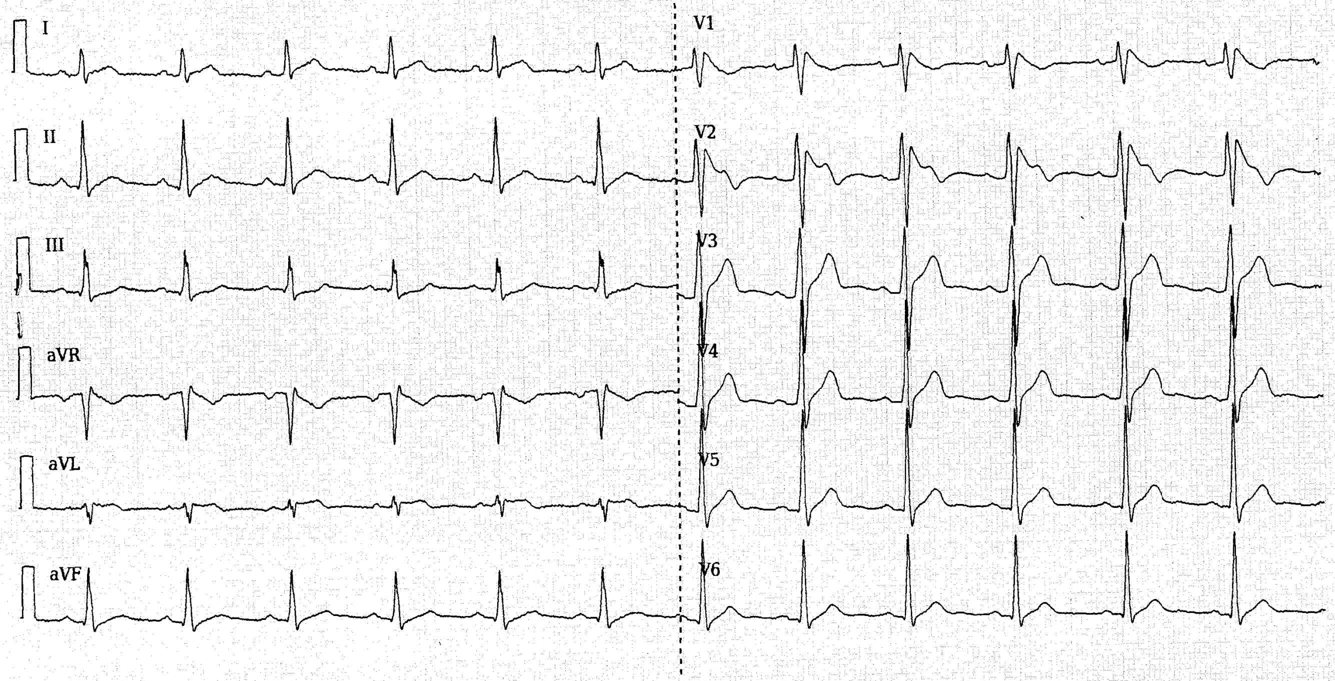

L: 10 mm/mV
C: 10 mm/mV
QTc: Hodges
KWH A&E3
Serial #:E8500-002337
25 mm/s

25005

ID: 11-01-2018 23:28:05 10

Male Years

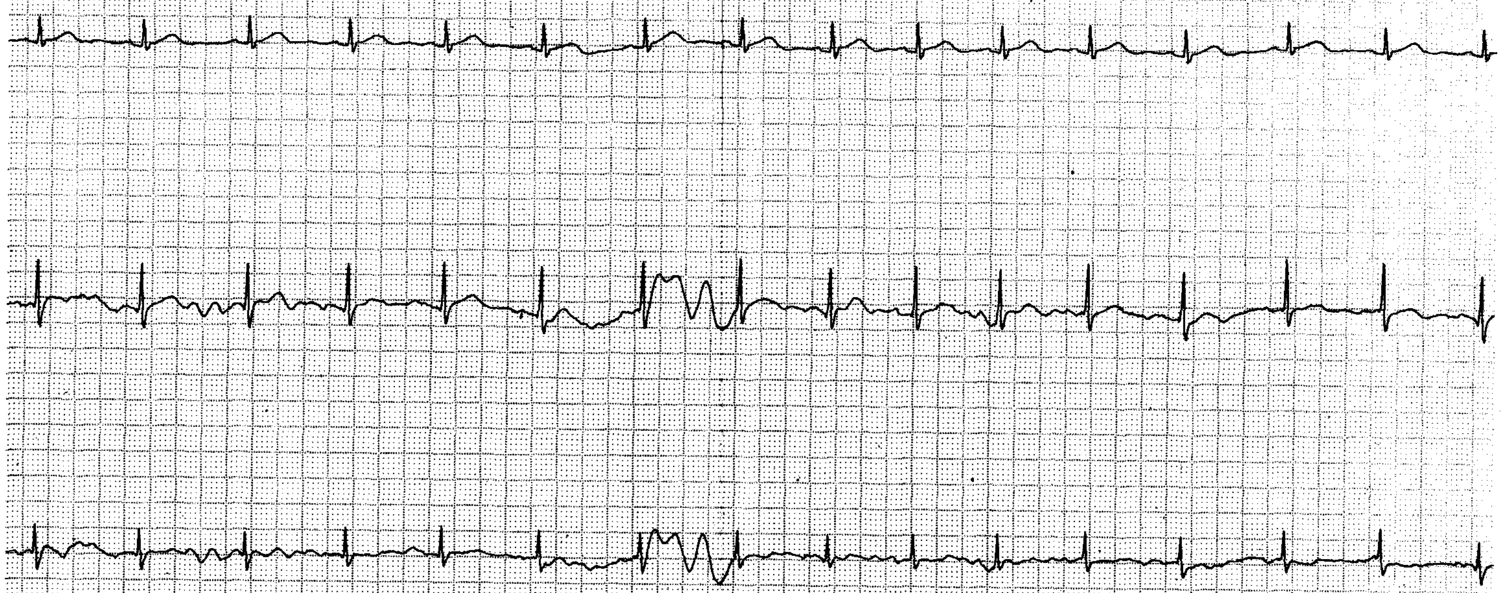

0.67–45Hz AC50 25mm/s 10mm/mV ♥83 SE-1200Express V2.2 SEMIP V1.81

13-01-2017 13:41:04
ID :
Name :
Age : Years

036

HR	: 79	BPM
P Dur	: 98	ms
PR int	: 130	ms
QRS Dur	: 96	ms
QT/QTC int	: 364/418	ms
P/QRS/T axis	: 67/89/50	°
RV5/SV1 amp	: 1.418/0.479	mV
RV5+SV1 amp	: 1.897	mV
RV6/SV2 amp	: 0.977/0.688	mV

Unconfirmed Report.

Diagnosis Information:
800: Sinus Rhythm
Normal ECG

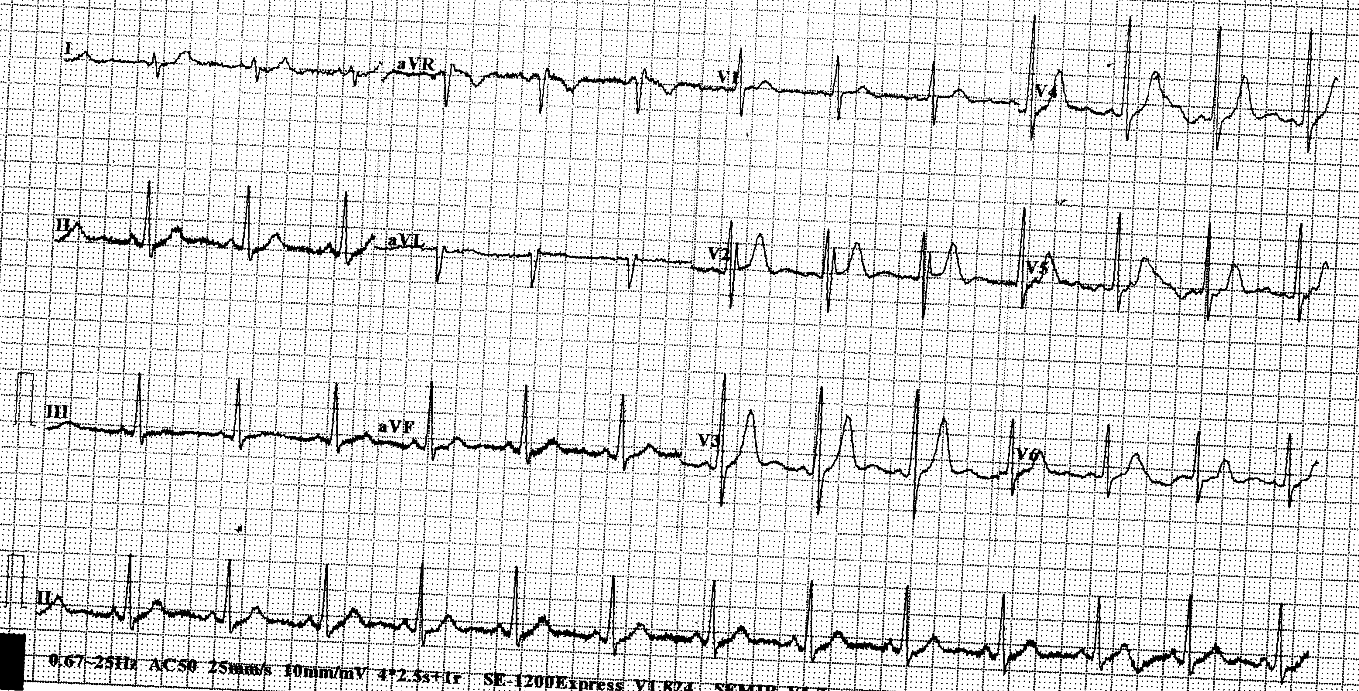

0.67~25Hz AC50 25mm/s 10mm/mV 4*2.5s+1r SE-1200Express V1.824 SEMIP V1.7

2012/12/16 20:13:41

Rate	82	. AGE IS NOT ENTERED, ASSUMED TO BE 50 YEARS OLD FOR PURPOSE OF ECG INTERPRETATION
PR	156	. SINUS RHYTHM.......................................normal P axis, V-rate 50- 99
QRSD	90	. EARLY PRECORDIAL R/S TRANSITION.........................QRS area positive in V2
QT	364	. ST ELEV, PROBABLE NORMAL EARLY REPOL PATTERN................ST elevation, age<55
QTc	425	

--AXIS--

P	77
QRS	64
T	52

- OTHERWISE NORMAL ECG -

Unconfirmed Diagnosis

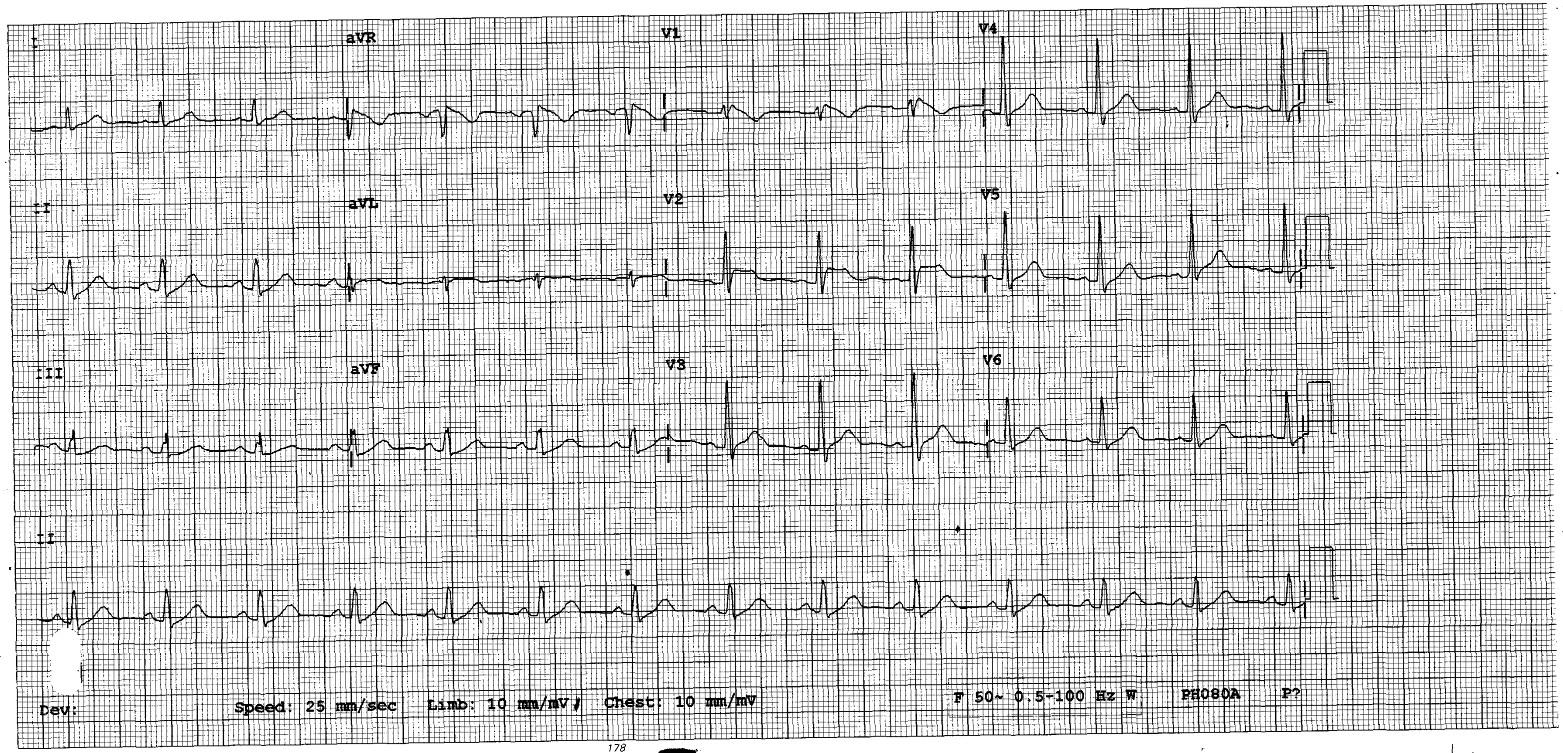

26222

23-03-2014 07:18:26 For Use On EDAN 01.57.32462 CE

267528

ID	:	Z3174884
Name	:	
Age	:	32 Years
Gender	:	Male

HR	:	72	BPM
P Dur	:	118	ms
PR int	:	187	ms
QRS Dur	:	101	ms
QT/QTC int	:	410/452	ms
P/QRS/T axis	:	79/91/68	°
RV5/SV1 amp	:	2.044/0.725	mV
RV5+SV1 amp	:	2.769	mV
RV6/SV2 amp	:	1.438/1.380	mV

Diagnosis Information:
800: Sinus Rhythm
203: Right Axis Deviation

Report Confirmed by:

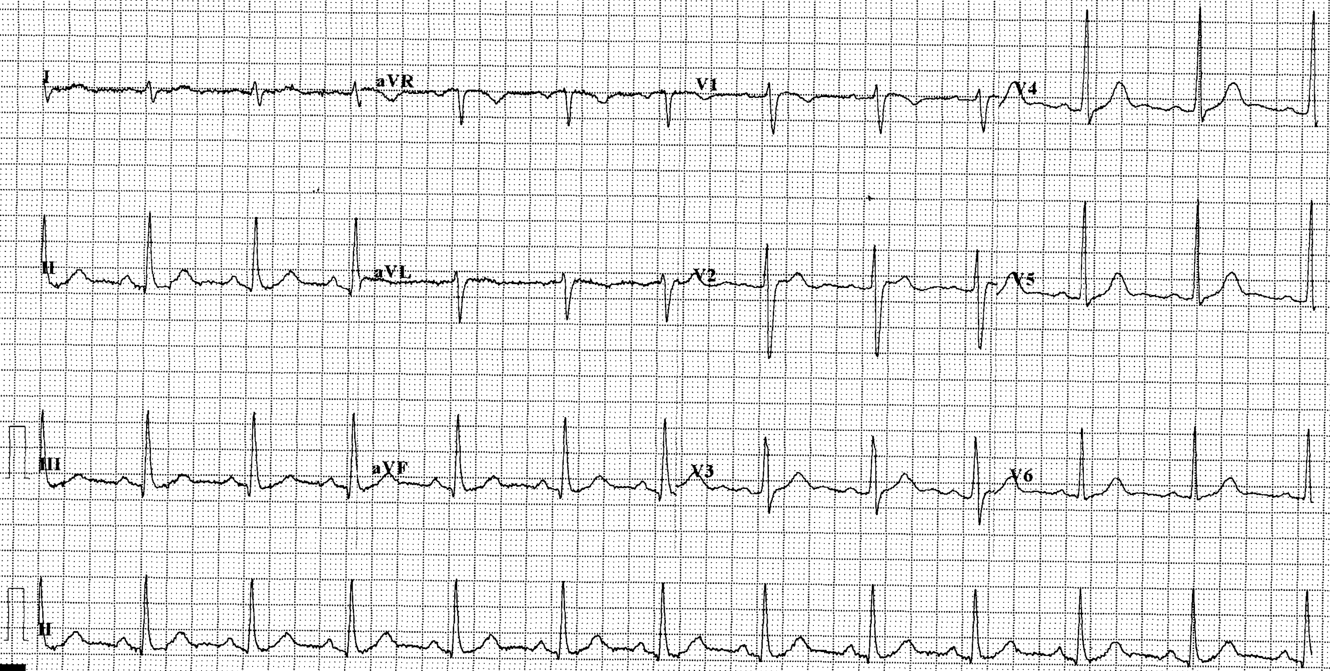

0.67~25Hz AC50 25mm/s 10mm/mV 4*2.5s+1r SE-1200Express V1.823 SEMIP V1.7

64006

30-12-2016 23:17:38
ID:

Years

HR	: 72	bpm
P	: 88	ms
PR	: 124	ms
QRS	: 101	ms
QT/QTc	: 374/412	ms
P/QRS/T	: 60/92/74	°
RV5/SV1	: 1.829/0.255	mV

Diagnosis Information:
Sinus Rhythm
Right Axis Deviation

054

Unconfirmed Report.

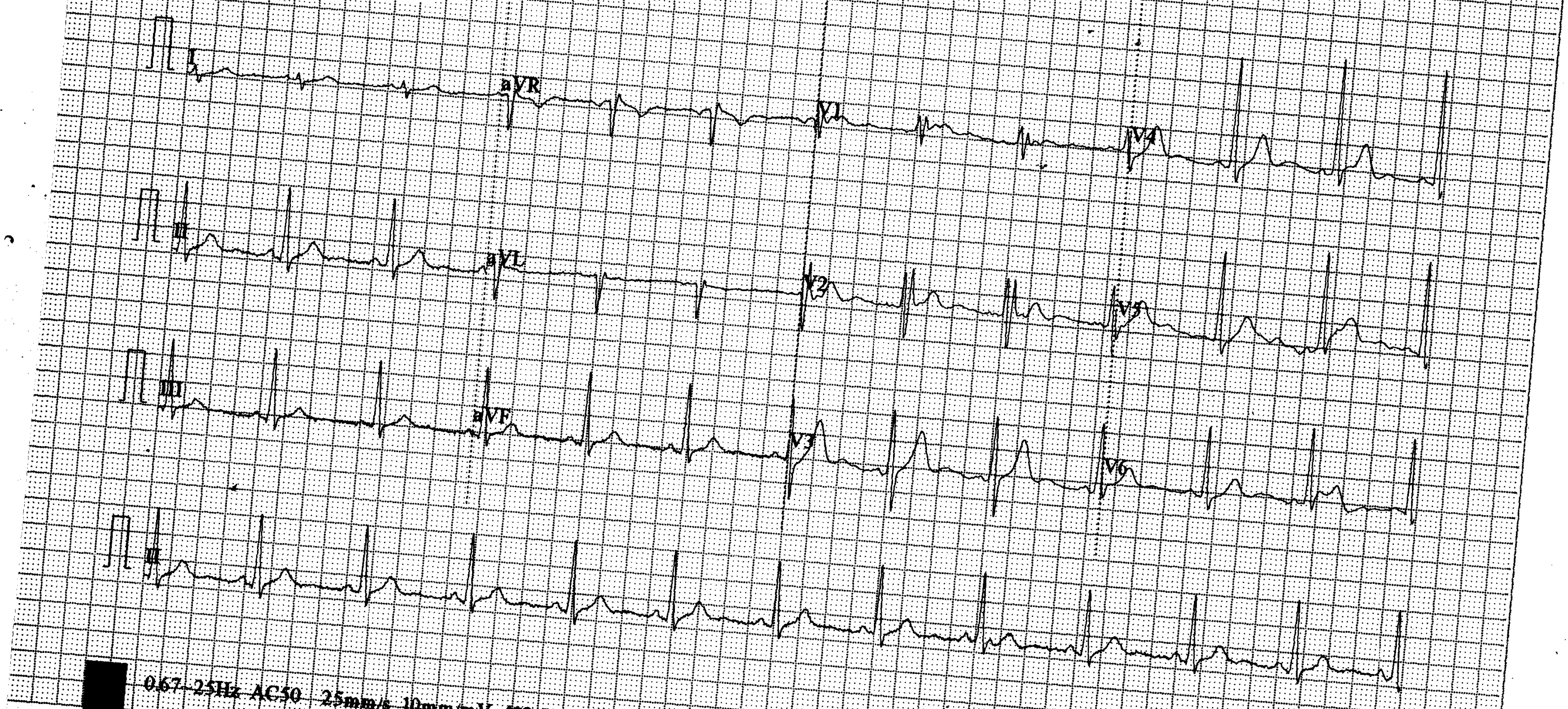

0.67-25Hz AC50 25mm/s 10mm/mV 4*2.5s+1r ♥72 SE-1200Express V2.01 SEMIP V1.7

28222

01/05/2013 01:42:28
Unknown

Rate	70	. AGE IS NOT ENTERED, ASSUMED TO BE 50 YEARS OLD FOR PURPOSE OF ECG INTERPRETATION
PR	140	. SINUS RHYTHM......................................normal P axis, V-rate 50- 99
QRSD	105	. RSR' IN V1 OR V2, RIGHT VCD OR RVH.................QRS area positive & R' V1/V2
QT	388	. ST ELEVATION SUGGESTS PERICARDITIS......................ST >0.10mV, ant/lat/inf
QTc	419	

--AXIS--

P	72
QRS	0
T	66

- ABNORMAL ECG -

Unconfirmed Diagnosis

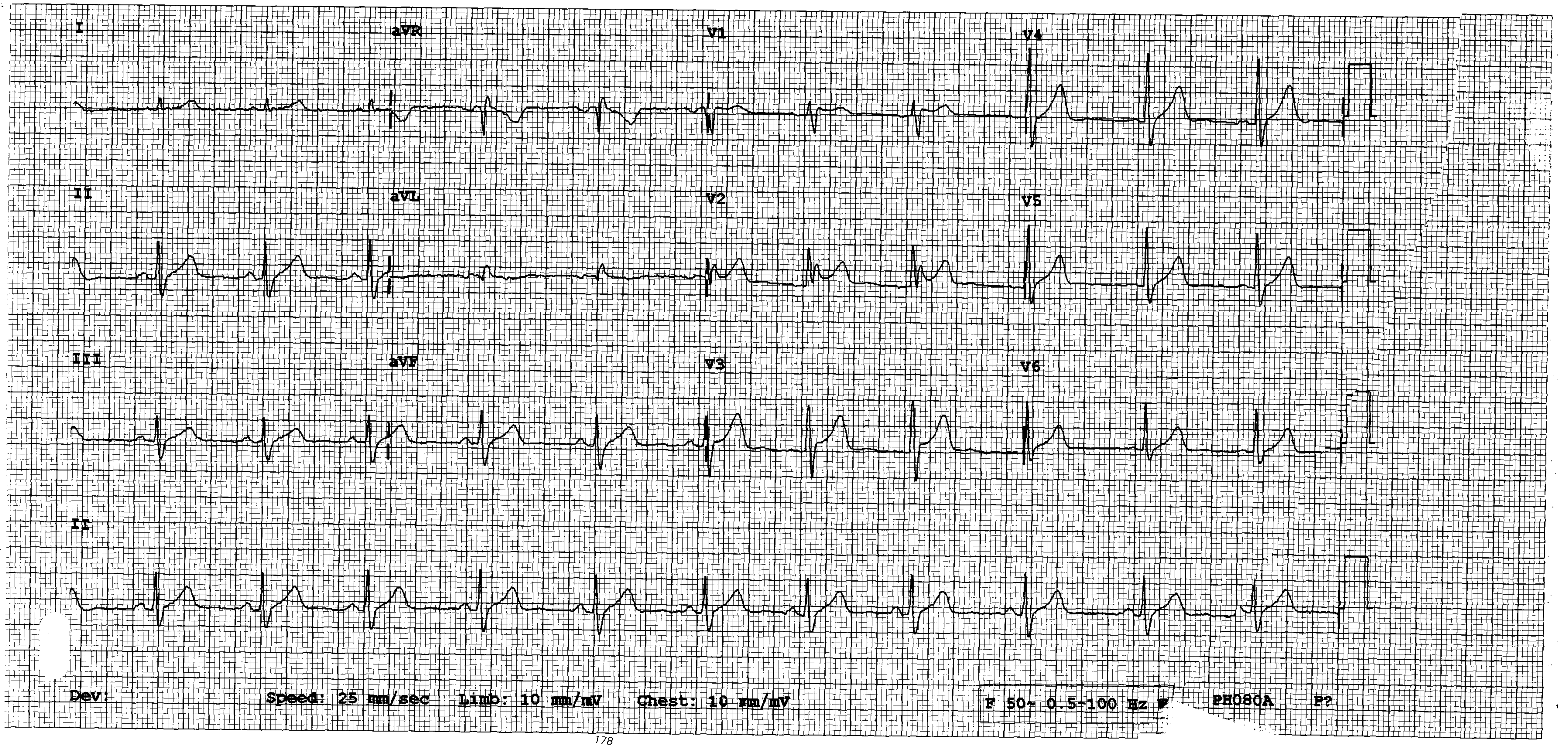

178

26222

ID: 3601 For Use On EDAN 01.57.32462 10-12-2016 20:58:36 0143359

Years (/ /)

HR	: 79	bpm
P	: 96	ms
PR	: 125	ms
QRS	: 95	ms
QT/QTc	: 366/420	ms
P/QRS/T	: 61/89/69	°
RV5/SV1	: 1.740/0.514	mV

Diagnosis Information:
Sinus Rhythm
Normal ECG

Report Confirmed by:

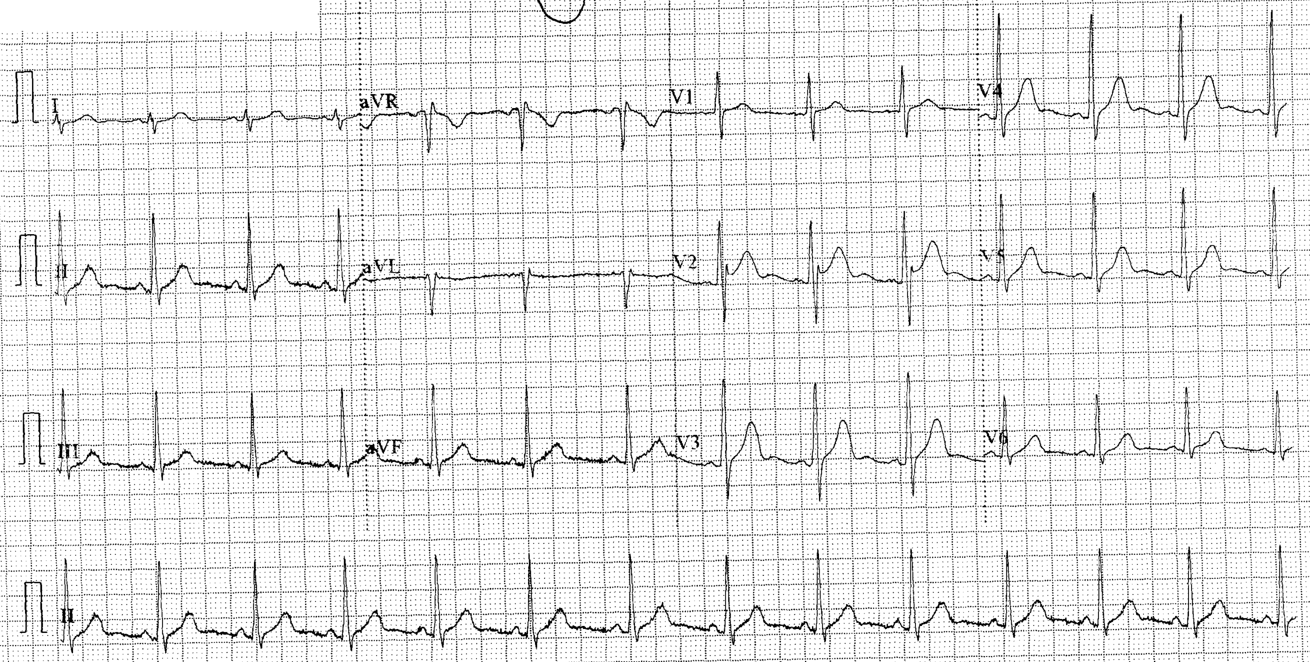

0.67~45Hz AC50 25mm/s 10mm/mV 4*2.5s+1r ♥79 Spacelabs Healthcare SL12 V1.2

39581
